# scIBD: a self-supervised iterative-optimizing model for boosting the detection of heterotypic doublets in single-cell chromatin accessibility data

**DOI:** 10.1186/s13059-023-03072-y

**Published:** 2023-10-09

**Authors:** Wenhao Zhang, Rui Jiang, Shengquan Chen, Ying Wang

**Affiliations:** 1 Department of Automation, Xiamen University, Xiamen, 361000 Fujian China; 2 National Institute for Data Science in Health and Medicine, Xiamen University, Xiamen, 361000 Fujian China; 3 Ministry of Education Key Laboratory of Bioinformatics, Research Department of Bioinformatics at the Beijing National Research Center for Information Science and Technology, Center for Synthetic and Systems Biology, Department of Automation, Tsinghua University, Beijing, 100084 China; 4 School of Mathematical Sciences and LPMC, Nankai University, Tianjin, 300071 China; 5 Xiamen Key Laboratory of Big Data Intelligent Analysis and Decision, Xiamen, 361005 Fujian China

**Keywords:** Single-cell, Chromatin accessibility, Doublets, Detection

## Abstract

Application of the widely used droplet-based microfluidic technologies in single-cell sequencing often yields doublets, introducing bias to downstream analyses. Especially, doublet-detection methods for single-cell chromatin accessibility sequencing (scCAS) data have multiple assay-specific challenges. Therefore, we propose scIBD, a self-supervised iterative-optimizing model for boosting heterotypic doublet detection in scCAS data. scIBD introduces an adaptive strategy to simulate high-confident heterotypic doublets and self-supervise for doublet-detection in an iteratively optimizing manner. Comprehensive benchmarking on various simulated and real datasets demonstrates the outperformance and robustness of scIBD. Moreover, the downstream biological analyses suggest the efficacy of doublet-removal by scIBD.

**Supplementary Information:**

The online version contains supplementary material available at 10.1186/s13059-023-03072-y.

## Background

Recent innovations in single-cell sequencing technologies have enabled the interrogation of genomic, epigenomic, transcriptomic, and proteomic heterogeneity at the unprecedented resolution of cellular level [1]. Droplet microfluidics, which allows for capturing and processing large numbers of individual cells in a massively parallel strategy with minimal reagent cost [2, 3], is one of the most widely used high-throughput single-cell sequencing technologies. Basically, only one cell/nucleus is supposed to be captured by one droplet when sequencing. Nevertheless, due to the technical restriction of droplet microfluidics, two or more cells/nuclei are frequently captured in one droplet and bound with the same oligonucleotide barcode sequence [4–6], creating a so-called doublet/multiplet that is disguised as one single cell. The presence of doublets can confound downstream analyses by, for example, constituting spurious cell clusters, interfering with the analysis of differential patterns, and obscuring functional enrichment analysis [7]. Therefore, detecting and removing doublets from single-cell sequencing data is an essential step to improve the accuracy of downstream analyses and thus reveal biological implications.

Single-cell RNA sequencing (scRNA-seq), which improves the understanding of the functional states of individual cells based on gene expression levels [8, 9], has chronically suffered from the doublet issue [7]. In vitro methods were initially proposed to detect doublets in scRNA-seq data. For example, Stoeckius et al. introduced Cell Hashing, where oligo-tagged antibodies that can uniquely label the samples are sequenced together with the cells, and the cross-sample doublets can be identified by assigning the cell to its original sample based on the sequencing results of such oligo-derived hashtags [10]. However, in addition to the extra cost when sequencing, Cell Hashing can only identify the sample-crossed doublets, failing to identify those intra-sample doublets originating from diverse cell types when the samples are too small or fragile to be split and recombined.

In silico methods were then proposed to improve the efficacy to detect doublets in scRNA-seq data. Demuxlet harnesses known natural genetic variation to identify the doublets [4]. However, the high droplet heterogeneity often requires extra bulk-sequencing to provide accurate single-nucleotide polymorphism (SNP) information as the reference for doublet-detection, making Demuxlet time- and cost-consuming. Thereby, a number of computational methods that are free of extra-biological implements have been proposed. The majority of existing methods are simulation-based and tackle the doublet-detection task by training a binary classifier using the original droplets as “singlets” and the simulated artificial doublets as “doublets” [7].

Similar to scRNA-seq data, single-cell chromatin accessibility sequencing (scCAS) data, which enables the investigation of epigenomic landscape in individual cells [11], is also confounded by doublets, especially for the prevalent application of droplet microfluidics. However, doublet-detection in scCAS data is more challenging than in scRNA-seq data since the assay-specific challenges of scCAS data, including their low capture rate, close-to-binary nature, extreme sparsity, and tens of times higher dimensions than scRNA-seq data [12–14]. Several doublet-detection methods have been developed specifically for scCAS data. These methods can be divided into two main categories: (1) simulation-based approaches, such as SnapATAC [15] and ArchR [6], which are similar to the major methods tailored for scRNA-seq data, and (2) read-based approaches, such as AMULET [16], which are based on the principle that the expected number of uniquely aligned reads covering any region in the genome will not exceed two for diploid. We note that both SnapATAC and ArchR are widely used pipelines for scCAS data analysis, and SnapATAC directly integrates Scrublet (a method for scRNA-seq data) [5] to detect doublets, while ArchR constructs a cell-by-bin matrix at a resolution of 500 bp, performs latent semantic indexing, and trains a classic *K*-nearest neighbors (KNN) classifier to detect doublets.

In this study, we focus on the more challenging task of doublet-detection in scCAS data. Besides, we focus on the detection of heterotypic doublets formed by cells of distinct types, lineages, or states, but not homotypic doublets formed by transcriptionally similar cells, since the gene expression profiles of homotypic doublets are similar to those of singlets of the same cell type and the existence of homotypic doublets does not much affect cell clustering [5, 7]. We also demonstrate that heterotypic doublets may confound the downstream analysis more seriously than homotypic doublets (Additional file 1: Fig. S1).

Although several methods have been proposed, there are still non-negligible limitations to be addressed. First, Xi and Li systematically benchmarked the performance of doublet-detection methods for scRNA-seq data and concluded that the methods exhibited a large variation in their performance, indicating that there is still room for methodology improvement even for scRNA-seq data [7]. Second, the simulation-based approaches ignore that not all original droplets are singlets; otherwise, we would not need doublet detection. Therefore, existing methods neglect the differences between original droplets and singlets and do not supply their classification algorithms with quality training data, resulting in biased classifiers [7, 17]. Third, the read-based approach, i.e., AMULET, although showing its advantage in detecting homotypic doublets, ignores the impact of cell division cycle and tends to detect the cells that are at interphase as false homotypic doublets. In addition, it often fails to detect the heterotypic doublets whose overlapped regions are rarely derived from distinct accessible profiles [16]. Fourth, the heterotypic doublet-detection performance has not been systematically benchmarked using synthetic and real scCAS datasets of diverse protocols, sizes, dimensions, qualities, and doublet rates.

To fill these gaps, we proposed scIBD, a *sc*CAS-specific self-supervised *i*terative-optimizing method to *b*oost the detection of heterotypic *d*oublets. As a simulation-based method, scIBD discards the routine random selection strategy that may yield excessive homotypic doublets in the simulation process. Instead, it uses an adaptive strategy to simulate high-confident heterotypic doublets and thus self-supervise for doublet-detection. Besides, scIBD adopts an iterative-optimizing strategy to detect the heterotypic doublets iteratively and finally outputs doublet scores based on an ensemble strategy. Extensive and comprehensive experimental results on 16 datasets, including fully-synthetic, semi-synthetic, and real scCAS data, demonstrate that scIBD can significantly outperform the current three state-of-the-art methods, including SnapATAC, ArchR, and AMULET, and provide the most robust performance. Furthermore, the downstream biological analyses, including cell clustering, differentially accessible region detecting, and functional enrichment analyses, show the realistic effectiveness of scIBD for scCAS data analysis. In addition, the extended application of scIBD on scRNA-seq data, demonstrate the robustness and versatility of scIBD.

## Results

### scIBD overview

**Fig. 1:**
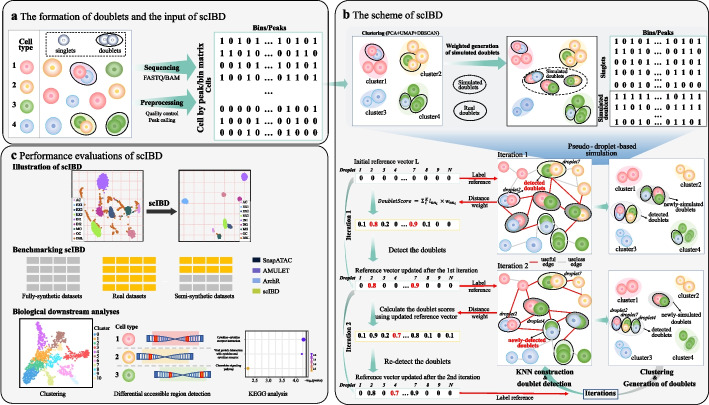
Overview of scIBD. **a** The formation of doublets in droplet-based scCAS. The input of scIBD is the cell by bin/peak matrix, which supports customized quality control and peak calling. **b** The scheme of scIBD. We present a pseudo-droplet simulation strategy where clustering is firstly performed, and then a bunch of artificial doublets are simulated, whose profiles are the union of the droplet profiles picked weighted by clusters. A reference vector of raw droplets is initialized with all values set to zero, indicating that all droplets have no contributions for detecting doublets primordially. In each iteration, scIBD computes doublet scores for all raw droplets based on their similarity to their nearest neighbors (KNN graph) and their previous scores (reference vector). The droplets with high doublet scores are detected as doublets, which no longer participate in the clustering in the following iterations. The artificial doublets are always re-created based on the newly clustering results in the current iteration. The reference vector is updated using the normalized doublet scores, which then influences the detection of doublets by participating in doublet score aggregating in the following iterations. **c** The performance evaluations of scIBD. We comprehensively benchmarked scIBD on three categories of datasets, including fully-synthetic, real, and semi-synthetic datasets derived from various scCAS data. Downstream biological analyses were conducted to further demonstrate the efficacy of scIBD

The overview of scIBD is shown in Fig. 1. The intrinsic formation of doublets is described, and the resulting count matrices processed from the BAM/fragment files are used as the input of scIBD (Fig. 1a). The detailed scheme of scIBD is shown in Fig. 1b. Different from the existing simulation-based methods that simulate doublets by mixing droplets randomly selected from the scCAS data, scIBD first performs droplet clustering and then simulates high-confident heterotypic doublets based on the clustering results. For the droplets and the simulated doublets, scIBD then constructs an adaptive KNN graph and detects the potential doublets in an iterative manner. In each iteration, the detected doublets no longer participate in clustering in the following iterations, and the subsequent clustering results can thus be improved without the noised doublets. Meanwhile, the better clustering results can then produce simulated heterotypic doublets with higher confidence, which in turn contribute to better doublet-detection performance in the following iterations. A reference doublet score list is used to record the doublet scores of the detected droplets and to participate in the KNN aggregation in the following iterations.

As shown in Fig. 1c, we extensively and comprehensively benchmarked scIBD with other three baseline methods, including SnapATAC [15], ArchR [6], and AMULET [16], on various datasets, including fully-synthetic, semi-synthetic, and real datasets derived from scCAS data (see the “Data collection and data processing” section). Details of the constructed 16 benchmark datasets are listed in Table 1, including the data size, doublet rate, median of sequencing depth, median of sparsity, number of annotated cell types, the valid read pair statistics of raw sequencing files, and the silhouette coefficient to measure the heterogeneity of the dataset. The fully-synthetic datasets were directly simulated based on the count matrices of the Forebrain dataset; thus, the read statistics of raw sequencing files are not available. Following the standard pipeline introduced in EpiScanpy [18], the UMAP plots of all semi-synthetic datasets are illustrated (Additional file 1: Fig. S2). In addition, we performed multiple biological downstream analyses, including clustering, differential accessible region detection, and KEGG analysis to further demonstrate the efficacy of scIBD. The baseline methods and the metrics used in benchmarking and downstream analyses are described in the “Methods” section.

**Table 1:** Benchmark datasets

Data source	Dataset identity	Dataset type	Number of droplets	Doublet rate	Med. of sequencing depth	Med. of sparsity	Number of peaks	Number of cell types	Complexity (silhouette coefficient)	Valid read pairs	Avg. valid read pairs per droplet	Med. valid read pairs per droplet
MF [19] (GSE100033)	SimATAC-balance	Fully-synthetic	4500	0.11	1172	0.9916	140,102	8	0.66	-	-	-
	SimATAC-imbalance		2100	0.2	1291	0.9908	140,102	8	0.56	-	-	-
HMC [6] (GSE162690)	High-loading	Real	20,663	0.29	1102	0.9941	187,075	10	0.31	175,950,050	8515	7510
	Low-loading		12,793	0.21	1004	0.9940	166,360	10	0.42	77,050,576	6022	4641
MF [19] (GSE100033)	Forebrain*	Semi-synthetic	1298	0.2	1930	0.9915	226,759	5	0.23	40,227,305	30,992	25,004
MCA [20] (GSE111586)	Bone marrow*		5244	0.2	2120	0.9917	254,413	18	-0.22	173,189,105	33,026	20,842
	Lung*		6146	0.2	1406	0.9918	171,002	22	-0.08	130,550,777	21,241	14,293
	Whole brain*		6494	0.2	2404	0.9873	189,933	21	0.01	255,248,909	39,305	21,679
	Cerebellum		2733	0.2	1465	0.9934	221,657	20	0.04	53,152,804	19,448	10,464
	Heart		9180	0.2	2029	0.9886	178,104	22	-0.30	236,918,887	25,808	15,143
	Kidney		7717	0.2	2702	0.9858	190,690	26	-0.24	215,602,830	27,938	19,557
	Prefrontal cortex		7150	0.2	4863	0.9765	206,546	22	-0.19	307,767,321	43,044	27,204
	Spleen		4824	0.2	2439	0.9845	157,363	15	-0.02	128,524,323	26,620	19,567
Islets [16] (GSE165212)	Islet1		5076	0.2	2494	0.9830	147,104	5	0.16	102,622,313	15,495	18,966
	Islet2		5145	0.2	2778	0.9804	138,549	5	0.13	101,595,484	17,755	12,137
10xPBMC [21, 22] (10xGenomics)	PBMC		9732	0.2	3112	0.9667	93,446	17	0.25	316,811,190	32,553	32,535

Asterisk (*) symbol implies that there are two sequencing replicates of this tissue: one is used to reserve singlets; the other one is used to generate doublets. The doublet rate values of semi-synthetic datasets indicate the simulated ratio based on the number of singlets, not the actual doublet rate of the dataset. The fully-synthetic datasets were directly simulated based on the count matrices, thus the read statistics are not available

### Illustration of scIBD on fully-synthetic datasets

We first used two fully-synthetic datasets as a proof of concept to demonstrate scIBD. The fully-synthetic datasets were constructed by simATAC [23] based on one of the two forebrain replicates of the MF dataset. For each one of the eight cell types in the forebrain replicates, we generated 500 singlets, and based on this, we then generated 500 doublets by randomly picking two singlets of different cell types and mixing their profiles. The concatenated matrix of all singlets and doublets is used as the balanced fully-synthetic dataset. For the imbalanced fully-synthetic dataset, we down-sampled the singlets from different cell types with random rates of 0.2, 0.4, 0.6, and 0.8, and then doublets are regenerated with the doublet rate of 0.2 and based on the sampled singlets.

**Fig. 2:**
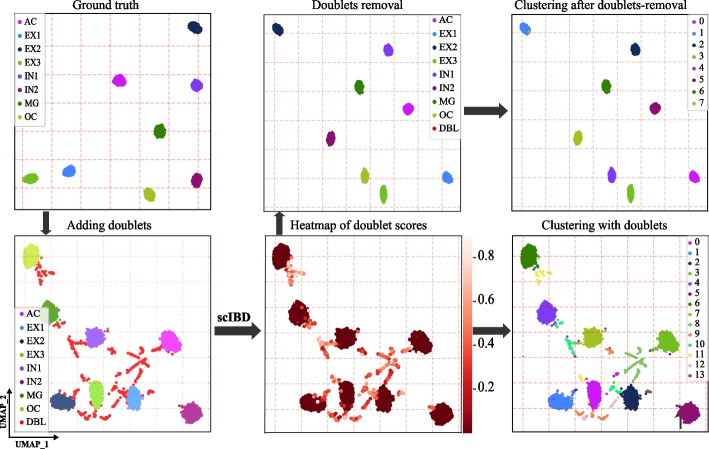
The overall illustration of scIBD on the fully-synthetic dataset. We visualized the fully-synthetic dataset in UMAP to illustrate the impact of doublets and the efficacy of doublet-removal by scIBD

We implemented scIBD on the two datasets to illustrate the effect of doublets and the efficacy of doublet removal by scIBD (Fig. 2, Additional file 1: Fig. S3). Therefore, in Fig. 2, we first visualized the simulated singlets, which show great heterogeneity among different cell types. After adding the simulated doublets, we can clearly observe the doublets scatter between the main cell types, indicating that the presence of doublets can complicate single-cell analysis by leading to the false appearance of distinct clusters or false connections between distinct cell types. We also present the heatmap of doublet scores provided by scIBD, where the droplets with high doublet scores are fairly consistent with the ground-truth doublets. To further illustrate the efficacy of scIBD for clustering, we removed the doublets called by scIBD using the ground-truth doublet rate. As clearly shown, the clusters reveal significant heterogeneity without the detected doublets, and the number of clusters obtained by default clustering resolution is exactly equal to that of ground-truth cell types. Besides, after doublet-removal, the performance of Louvain clustering with default resolution was significantly improved (ARI and AMI were improved by 22% and 17%, respectively). On the balanced dataset, the precision is 0.99 and the recall is 0.99. Although some doublets failed to be detected, the remained doublets have negligible impact on the clustering results (compared with the clustering results on pure singlets, the performance after doublet-removal by scIBD only decreased 1.5% and 2.7% for ARI and AMI, respectively). Similar results can be obtained on the imbalanced dataset where the precision and recall are both 0.97 when we call the doublets using the ground-truth doublet rate (Additional file 1: Fig. S3). Since the simATAC-simulated datasets are formed as count matrices, which do not apply to ArchR and AMULET, we provide more benchmark evaluations on the datasets with BAM files in the following sections.

### Real scCAS HMC datasets to benchmark scIBD

**Fig. 3:**
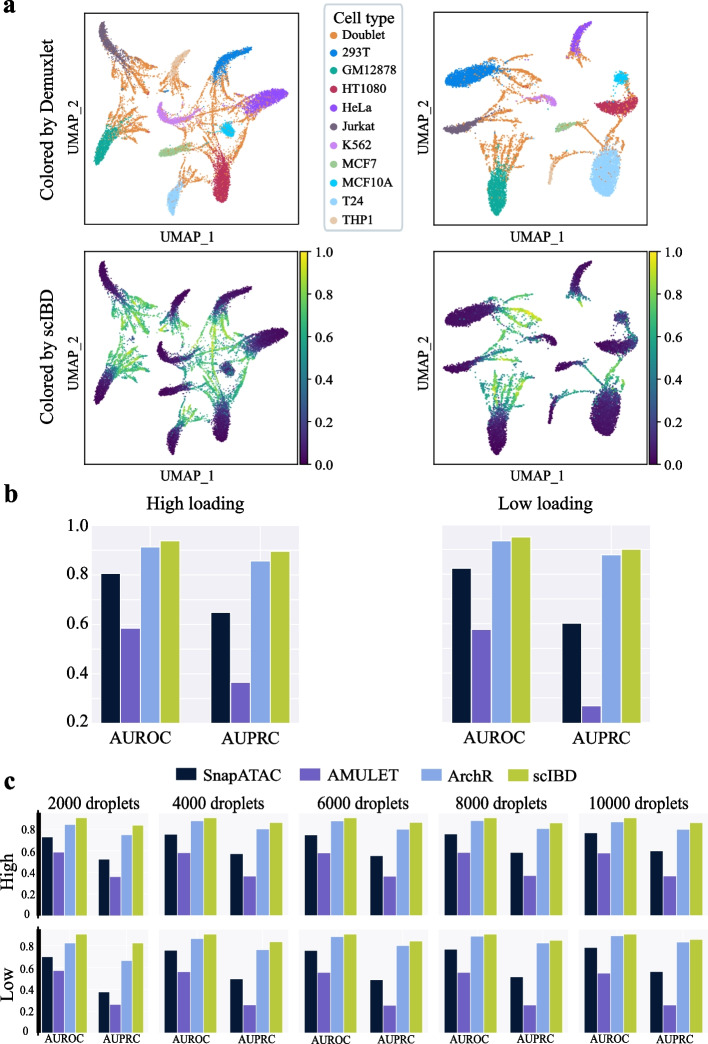
Performance evaluation on real HMC datasets. **a** The UMAP visualization of the two datasets where the droplets are colored by Demuxlet-annotated labels, and the doublet scores produced by scIBD, respectively. **b** The AUROC and AUPRC comparison with baseline methods. **c** The performance comparison on the datasets sub-sampled from the original sets

We further used the real HMC datasets to perform a ground-truth performance comparison between scIBD and baseline methods. The HMC datasets were generated by 10x Genomics scATAC-seq on a mixture of ten human cell lines loaded at high and low concentrations (high-loading and low-loading), respectively. The doublet annotations were provided by Demuxlet, which uses natural genotype variation information from extra bulk sequencing to detect the doublets. Generally, Demuxlet can only detect the doublets that were formed from different donors/samples with distinct genotype variations. Considering the generation of the HMC datasets that ten human cancer cell lines were mixed to be sequenced, the doublets annotated by Demuxlet are basically those doublets originating from different cell lines (donors), which can be regarded as the heterotypic doublets. As shown in Fig. 3a, we exclusively visualized the datasets colored by the cell annotations that Demuxlet produced and the doublet scores achieved by scIBD. The datasets revealed distinct cell heterogeneity according to the high relevance between clusters and cell type annotations. Although Demuxlet was supposed to detect the inter-cell-line doublets, it still regarded some droplets within the unique cell line as doublets (Fig. 3a upper panels). Unlike Demuxlet, scIBD tends to detect more heterotypic doublets that are located at the edge of the clusters or between them (Fig. 3a bottom panels).

HMC datasets produce fragment files, allowing us to benchmark scIBD with the baseline methods. As shown in Fig. 3b, scIBD outperforms the baseline methods both in AUROC and AUPRC when only considering the Demuxlet annotations. Specifically, even though the baseline method ArchR achieved good performance for detecting doublets in these datasets profiled in the study of ArchR (both AUROC and AUPRC were close to 0.9 on the two datasets), scIBD still improved the AUROC and AUPRC by nearly 3% on the high-loading dataset and 2% on the low-loading dataset. We note that all the methods often had AUPRC scores much lower than their AUROC scores on each dataset, an expected phenomenon given the imbalance between the number of singlets and doublets. Since AUROC is usually an overly optimistic measure of accuracy under such imbalanced scenarios [24], we will mainly focus on AUPRC in the following discussion. As expected, we also observed that AMULET performed poorly on the HMC datasets, since it fails to detect the doublets originating from distinct accessible profiles. In contrast, the simulation-based methods that study accessible patterns based on profile matrices showed their superiority on these real datasets.

To evaluate the performance on datasets of different sizes, based on these two HMC datasets, we further randomly down-sampled the droplets and obtained subsets with the droplet numbers of 2000, 4000, 6000, 8000, and 10000, respectively. As shown in Fig. 3c, scIBD consistently outperformed the baseline methods in all down-sampled subsets evaluated by both AUROC and AUPRC. The results indicate that scIBD is robust to the sample size of scCAS data. Specifically, scIBD can adaptively apply the PCA strategy in KNN graphing on the datasets where the cell type heterogeneity is significant.

### Semi-synthetic scCAS datasets to benchmark scIBD

The real HMC datasets originating from different human cell lines have distinct cell heterogeneity, making the epigenomic profiles of most heterotypic doublets disparate from those of singlets and making the doublets much easier to be detected. To further benchmark scIBD, we further conducted a comprehensive performance evaluation on semi-synthetic scCAS datasets. The datasets were built upon the Forebrain and MCA datasets, which were generated from different tissues of mice. The cell types in these datasets are more complex and indistinguishable; the bone marrow dataset has the differentiating blood cells that are on a continuous trajectory. To benchmark the performance of scIBD on the datasets with different doublet rates, we constructed the semi-synthetic datasets by simulating the doublets using different doublet rates ranging from 0.05 to 0.25 with an interval of 0.05, based on the number of droplets. Particularly noteworthy is that the PCoA strategy was automatically applied to the semi-synthetic datasets.

**Fig. 4:**
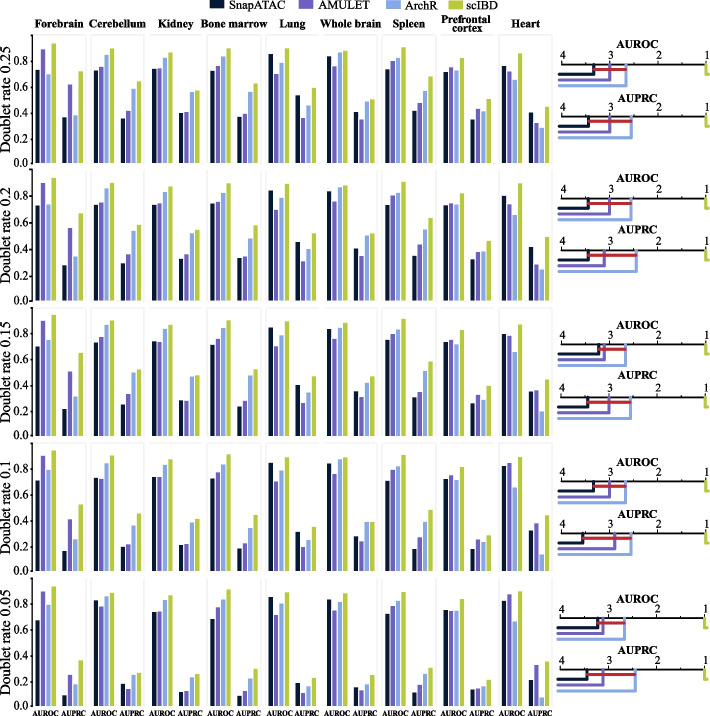
Performance evaluation on the semi-synthetic datasets with different doublet ratios. Different simulation ratios of doublets ranging from 0.05 to 0.25 with an interval of 0.05, are implemented on nine datasets. The histograms and the critical difference diagram over the datasets demonstrate the outperformance of scIBD

As depicted in Fig. 4, scIBD provided superior performance than all the baseline methods across all scCAS datasets with different doublet rates. Following Fawaz et al. [25], the critical difference diagrams are further depicted to illustrate the overall performance of the methods. In the critical different diagram, the horizontal axis indicates the mean ranking of each method across all datasets, and the insignificant difference between a group of methods in terms of performance is marked by a red horizontal line based on a Wilcoxon signed-rank test with Holm’s alpha (5% two-sided) correction. The critical difference diagrams under different doublet rates further demonstrated the significant advantages of scIBD over baseline methods. We note that the average depth across all *loci* in the Forebrain dataset is 7.4 and is nearly triple that of the MCA datasets (2.45), and AMULET can thus find enough overlapped regions in the Forebrain dataset to identify doublets based on the reads, yielding better performance on this dataset, while the simulation-based ArchR and SnapATAC, as expected, surpassed AMULET on the MCA datasets. Importantly, scIBD outperforms all the baseline methods on datasets with different depths and different doublet rates, again suggesting the advantages of scIBD.

To further understand the performance of scIBD and the baseline methods, apart from AUROC and AUPRC, we present more metrics, including precision, recall, and F1, to evaluate the results on the semi-synthetic datasets that were simulated with the 20% doublet rate based on the number of singlets (Table 1). The doublet-detection performance is usually evaluated by selecting a reasonable cutoff, i.e., the expected doublet rate, while this parameter is unknown in realistic scenes. Therefore, we enumerated the cutoff, from 0.05 to 0.25 at an interval of 0.05, to calculate the performance metrics of the methods. Specifically, we ranked the droplets by their scores from the methods. Then, we set a cutoff based on the specific calling rate. Any droplet with a score above the cutoff was considered a doublet.

scIBD outperforms the baseline methods in terms of precision, recall, and F1 at most doublet calling rates across different datasets (Additional file 2: Table S1). In particular, compared with the baseline methods, scIBD can achieve higher recall with the increase of calling rate, showcasing its superiority in removing more doublets when the real doublet rate is unknown. SnapATAC (Scrublet) shows fluctuant performance across different datasets for it was initially proposed for scRNA-seq data, while the rest three methods show relatively robust performance, among which scIBD achieves the overall best performance. In addition, the corresponding AUROC and AUPRC plots of the semi-synthetic datasets are illustrated (Additional file 1: Fig. S4).

### scIBD has robust performance on detecting doublets with different numbers of captured reads

Under the assumption that doublets can obtain a significantly greater number of sequenced reads than singlets, the doublets in the above semi-synthetic datasets are simulated by directly mixing reads of two singlets. The statistical results based on the HMC datasets whose doublets are labeled by Demuxlet also support such presupposition. However, the specific extent of the read number fold-change between doublets and singlets in real scCAS data is uncertain. To demonstrate the robustness of scIBD to detect doublets with different numbers of captured reads, we down-sampled the reads of doublets using the rate ranging from 0.1 to 0.4 with an interval of 0.05 based on the semi-synthetic datasets with the doublet rate of 20%.

**Fig. 5:**
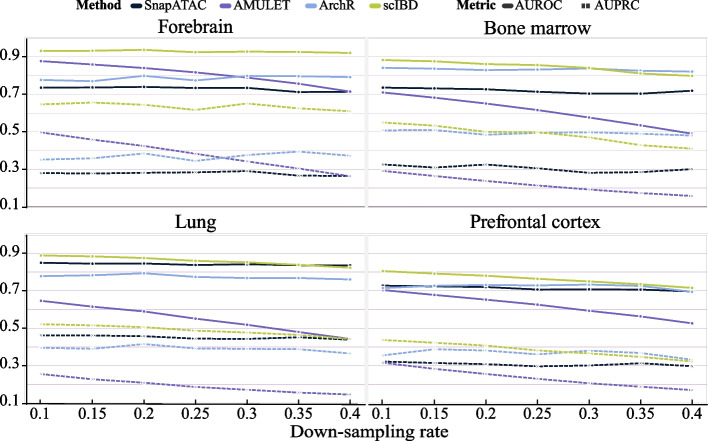
Performance evaluation on the semi-synthetic datasets where doublets have different numbers of captured reads. Based on the semi-synthetic datasets, the reads used to form artificial doublets are down-sampled with a ratio ranging from 0.1 to 0.4 with an interval of 0.05. The AUROC (solid lines) and the AUPRC (dotted lines) show the trend of performance with the reads decrease of doublets

scIBD overall outperformed the baseline methods when the down-sampling rate was below 0.3 (Fig. 5, Additional file 1: Fig. S5). When the down-sampling ratio increased over 0.3, the ranking test results according to the critical difference diagrams still showed that scIBD achieved the best performance in most cases. However, the insignificant difference between the performance of scIBD and the baseline methods (marked by thick horizontal lines) shown in the critical difference diagrams which are depicted based on a Wilcoxon signed-rank test with Holm’s alpha (5% two-sided) correction also indicates that the dissimilarity between doublets and singlets is negligible when the reads enrichment in doublets is low, making the doublets hard to detect. Additionally, the dramatic decline in the performance of AMULET demonstrates the sensitivity of read-based methods, while the simulation-based methods leveraging profile patterns show better robustness to the number of captured reads.

### Benchmarking on the rigorously quality-controlled semi-synthetic datasets

Considering that the benchmarking experiments on the previous semi-synthetic datasets (MF and MCA) still lack the evaluation of the singlets, i.e., the droplets annotated with singlets may still contain doublets, which yields biased evaluation results. Therefore, we further benchmarked scIBD on three additional datasets, Islet1, Iselt2, and peripheral blood mononuclear cells (PBMC), where the singlets were more strictly selected. Specifically, apart from conserving the droplets with clear cell type annotations, we also filtered out the possible doublets annotated by AMULET and ArchR. On the Islets datasets, the doublet annotations by ArchR and AMULET have been reported by Thibodeau et al. [16], and we directly removed the reported doublets. On the PBMC dataset, AMULET was performed with the default parameters, and AcrhR was performed with the expected doublet of 10% (default is 5%) in advance to remove more possible doublets. The remaining droplets were used as high-confidence singlets to establish such rigorously quality-controlled benchmark datasets, and the ground-truth doublets were constructed following the pipeline in the “Construction of benchmark datasets” section.

**Fig. 6:**
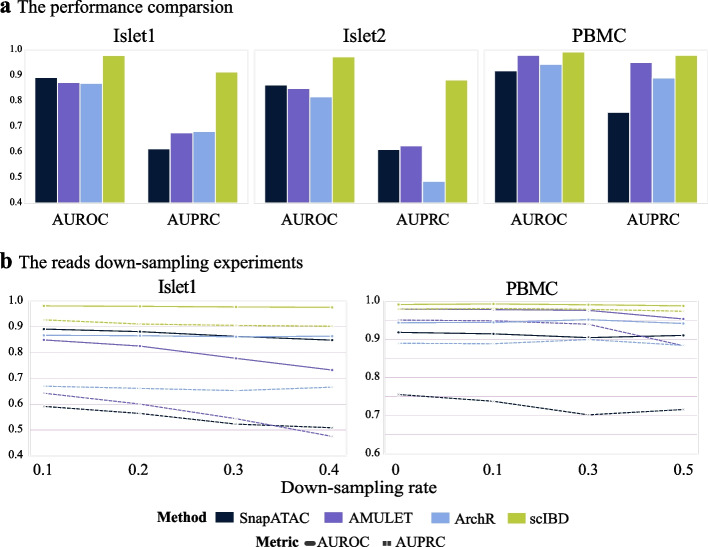
Performance evaluation on the rigorously quality-controlled semi-synthetic datasets of Islets and PBMC. **a** The AUROC and AUPRC comparison of scIBD and the baseline methods on the rigorously quality-controlled datasets of Islet1, Islet2, and PBMC, where the ground-truth singlets were strictly selected. **b** The performance evaluation on the read-down-sampled Islet1 and PBMC datasets. The reads of Islet1 dataset were down-sampled with a ratio ranging from 0.1 to 0.4 with an interval of 0.1; and the reads of PBMC dataset were down-sampled with a ratio ranging from 0.1 to 0.5 with an interval of 0.2. The AUROC (solid lines) and the AUPRC (dotted lines) show the trend of performance with the decrease in sequencing depth

scIBD significantly outperforms the baseline methods on the rigorously quality-controlled benchmark datasets (Fig. 6a, Additional file 2: Table S2). On the Islet1 dataset, scIBD achieves the AUROC 0.978, which is 10% higher than that (0.891) of the second-best method (SnapATAC), and achieves the AUPRC 0.913, which is 34% higher than that (0.680) of the second-best method (ArchR). On the Islet2 dataset, scIBD achieves the AUROC 0.973, which is 13% higher than that (0.862) of the second-best method (SnapATAC), and achieves the AUPRC 0.882, which is 41% higher than that (0.624) of the second-best method (AMULET). On the PBMC dataset, which has deeper sequencing depth (about 30k median valid read pairs per droplet, 15k of Islets), AMULET performs much better than on the Islets datasets and significantly outperforms SnapATAC and ArchR, but scIBD still outperforms AMULET both on AUROC and AUPRC. The detailed metrics of precision, recall, and F1 are also presented (Additional file 2: Table S1).

We further conducted the down-sampling experiments on Islet1 dataset and PBMC dataset to investigate the effect of sequencing depth on the performance of scIBD and the baseline methods. On the Islet1 dataset that has lower sequencing depth (about 15k valid read pairs per droplet), we down-sampled the reads of all droplets using the rate ranging from 0.1 to 0.4 with an interval of 0.1; on the PBMC dataset that has deeper sequencing depth (about 30k valid read pairs per droplet), we down-sampled the reads more drastically from 0.1 to 0.5 with an interval of 0.2.

As shown in Fig. 6b, scIBD achieves the best performance no matter in terms of AUROC and AUPRC on the datasets with different sequencing depths. On the Islet1 dataset, scIBD outperforms the secondary method by about 10% on the mean of AUROC and even about 35% on the mean of AUPRC. On the PBMC dataset, AMULET achieves the secondary-best performance for the high sequencing depth. However, its performance decreases drastically when the down-sampling rate increases up to 0.5 (the sequencing depth has decreased to 15k median read pairs per droplet). After all, the down-sampling experiments demonstrate that AMULET can achieve better performance (secondary to scIBD) on the deeper-sequenced data, especially when the sequencing depth is above 15k median valid read pairs per droplet. However, the simulation-based methods show the relatively high robustness when handling data with different sequencing depths, among which scIBD achieves the overall best performance.

Taken together, scIBD has robust performance on scCAS data no matter how significant the heterogeneity is, how big the sample size is, how high the doublet rate is, or even how variable the sequencing depth is. Specifically, by applying the specific PCoA strategy in KNN graphing, scIBD shows its superiority over the baseline methods on the complex scCAS datasets in which the heterogeneity of cells is insignificant, making it more realistically applicable. We will demonstrate its practical applications in downstream analyses in the following section.

### Biological downstream applications of scIBD

Heterotypic doublets mostly hinder clustering by creating spurious profiles, misleading the subsequent marker peak/region identification, and downstream functional enrichment analyses. To further evaluate the impact on the downstream analyses by the doublet-detection methods, we hereby conduct comprehensive downstream analyses based on the semi-synthetic Forebrain dataset that was simulated with the doublet rate of 20%.

### Improvement of clustering

We performed cell clustering according to the widely used scCAS analysis pipeline [12–14, 20, 26]. Specifically, we performed TF-IDF for data normalization, PCA for cell embedding, and Louvain for clustering. We followed this pipeline to get the clustering results on the dataset only containing singlets, the dataset containing doublets, and the doublet-removed datasets implemented with ArchR, AMULET, SnapATAC, and scIBD, respectively. To obtain the desired number of clusters for benchmarking, we adopted a widely used binary search strategy to tune the resolution parameter in Louvain clustering to make the number of clusters and the number of ground-truth cell types as close as possible [12, 13, 18, 27].

Most doublet-detection methods provide the pre-set parameter, i.e., the expected doublet rate, to control the number of doublets to be detected, and so does scIBD. scIBD outperforms the baseline methods at all calling rates except for 0.05 on the Forebrain dataset (Additional file 2: Table S1). Specifically, when calling doublets at the rate of 5%, we ranked the doublet scores given by the methods and obtained the 5% quantile threshold to select droplets with scores higher than the threshold as doublets. However, the doublet scores, obtained by AMULET, assigned over 8% droplets the score of one (definitely identified as doublets) for the Forebrain dataset was deeply sequenced (about 25k median read pairs per droplet). Therefore, more droplets (8%) are detected as doublets at the 5% calling rate using AMULET, yielding better performance at this specific calling rate. The results also demonstrate the superiority of AMULET to detect a conservative rate of doublets when the real doublet rate remains unknown, while scIBD shows its superiority when aiming at detecting more doublets for its higher recall.

**Fig. 7:**
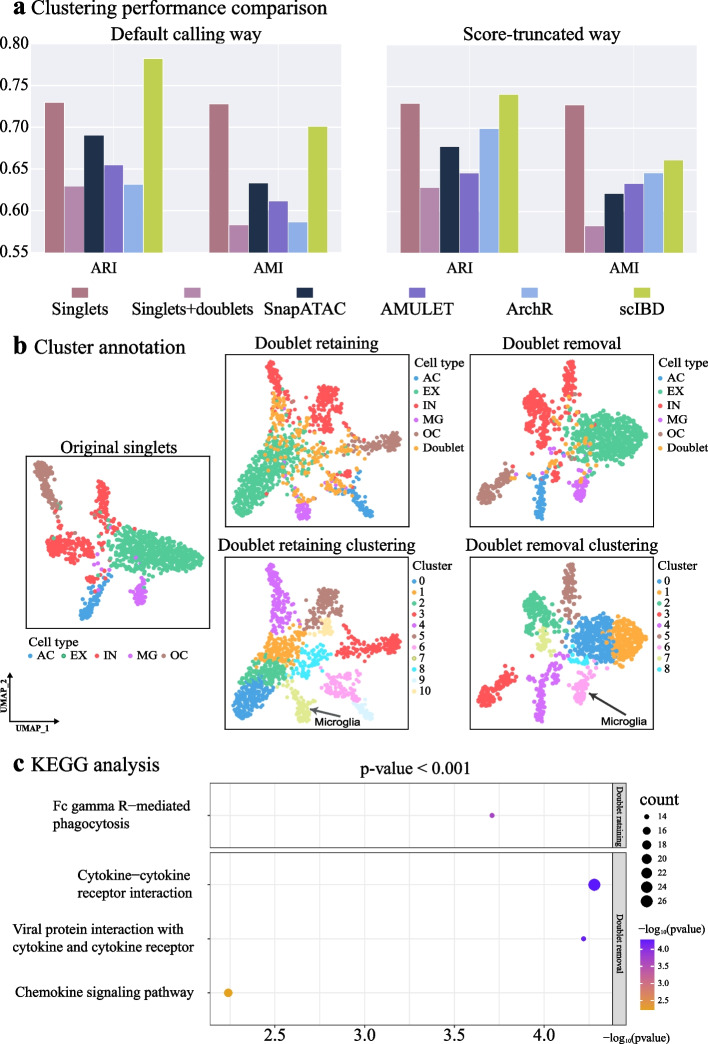
Downstream biological analyses. **a** The performance comparison on clustering picked by the removal of doublets. **b** Using ground-truth labels (upper) as the reference to annotate the microglia cluster (lower), and a series of downstream biological analyses were performed. **c** The KEGG enrichment results using the differential accessible regions detected on the doublet-retaining dataset and the doublet-removal dataset, respectively

To further evaluate the doublet-removal efficacy by scIBD and the baseline methods, we calculated ARI and AMI based on the cell type labels and clustering results. As shown in Fig. 7a, the confounding effect of doublets can be shown by the significant decrease presented in metrics, and scIBD significantly outperformed the baseline methods on all metrics using different doublet calling ways. Specifically, in the default calling way, namely, detecting doublets by giving a pre-specified doublet rate for the methods, scIBD can improve the ARI and AMI scores by 15% and 11% compared to the second-best method, respectively. By removing the doublets, scIBD nearly achieved the clustering results approximate to those obtained on the dataset only containing singlets. In the score-truncated way, namely, detecting doublets by a strictly post-specified number of doublets according to the doublet scores, scIBD also improved both metrics by about 5% compared to the second-best method. All these results demonstrate that doublets seriously hinder cell clustering, and detecting and removing doublets by scIBD can effectively benefit scCAS analysis.

### Improvement of identifying differentially accessible regions

We further conducted a case study on the Forebrain dataset to show that scIBD can assist in identifying the differentially accessible regions. Microglia (MG), the resident macrophage of the central nervous system [28], plays critical roles in brain development, and physiology during life and aging [29], and was used in the case study. We identified the differentially accessible regions of the cluster, which is annotated as MG according to the ground-truth labels (Fig. 7b), versus the rest clusters, on the datasets of ground-truth singlets (GT), doublets retaining (RE), and doublets removal (RM) by scIBD and the baseline methods, respectively. The ground-truth singlets dataset only contains the cells with definite cell type annotations, and the results performed based on which can act as a baseline. After removing the doublets, we compared the overlapped differential accessible regions detected between different RM datasets and GT (Additional file 2: Table S3). Notably, scIBD outperformed the baseline methods in recovering the differential accessible regions by achieving the most overlaps between GT.

To further demonstrate scIBD, we analyzed the top twenty differential accessible regions on the RM dataset by scIBD. RE and RM have ten overlapped differential regions with GT (marked in bold) among the top twenty differential regions (Additional file 2: Table S4). On the one hand, the overlaps with GT reveal that most differential accessible regions are obtained when the doublets account for a small portion of this cluster and the characteristic of MG can be identified. On the other hand, the difference also demonstrates the confounding effect of doublets. Importantly, the overlapped differential regions with GT of RM mostly rank at the top of the list, indicating the high efficacy of scIBD to remove the doublets that interfere with the differential region detection. For the nine peaks (marked with *) that were identified after doublets removal compared to doublets retaining, using NCBI Genome Data Viewer [30], we mapped the peak regions to the relevant genes, among which three (*Tmem135*, *Asb2*, and *Heatr1*) are encoding genes. The mutation of *Tmem135* has been reported to be relevant to the function of microglia, inducing its transformation in morphology and migration in mouse retina [31, 32]. *Asb2* has been identified as significantly differentially expressed in microglia in many assays [33, 34]. Song et al. also reported that *Asb2* is the core gene involved in the upregulated modules by performing WGCNA on microglia, indicating the key role of microglia-neuron interaction in the process of Purkinje cell degeneration [35]. As a glioma-associated antigen, *Heatr1* can induce functional cytotoxic T lymphocytes [36], and as immune cells, the microglia-T cell interaction and their reciprocal signaling effect have been studied and reported in many researches [37, 38]. Taken together, scIBD can effectively eliminate the impediment of doublets and provide more accurate candidates in identifying differentially accessible regions.

### Improvement of KEGG analyses

To further evaluate the efficacy of scIBD, we performed the KEGG enrichment analyses subsequently. The number of differentially accessible regions to be used is expanded to 200 so that the mapped relevant genes are enough to be enriched to find critical KEGG pathways terms. We performed KEGG pathway enrichment analysis using the web server tool g: Profiler [39]. To obtain the most-confident enriched pathways and illustrate the difference, we set a strict threshold of $p$-value as 0.001 (two-sided). Based on the differentially accessible regions from RE, only one KEGG pathway term can be obtained when $p$-value $<0.001$, while three terms are enriched on the RM using the same criteria, as shown in Fig. 7c. We note that cytokines, a group of small proteins that are usually expressed by microglia under inflammatory conditions, are essential signaling modules for microglia [40]. Coincidently, among the newly found terms after removing doublets, “cytokine-cytokine receptor interaction” and “viral protein interaction with cytokine and cytokine receptor” are cytokines-related and have been reported to be associated with microglia [41–43]. For instance, Khayer et al. find that “cytokine-cytokine receptor interaction” is a significantly enriched pathway that participates in microglia activation in triggering neurodegenerative diseases [41]. “Chemokine signaling pathway” is a union of pathways including the fractalkine signaling pathway, and fractalkine is mostly expressed by neurons and its unique receptor *CX3CR1* is exclusively expressed by microglia in the central nervous system [44, 45].

The above results demonstrate that scIBD can accurately detect the doublets that hamper the downstream biological analyses and thus improve the biological downstream analyses effectively.

### Extended application of scIBD on scRNA-seq data

scIBD is a specially designed doublet detection tool for scCAS data. To discover the generalizability of scIBD, we also investigate the application of scIBD on scRNA-seq data. Following the review paper by Xi et al. [7], we selected two datasets, referred to as hm-12k and nuc-MULTI, to further benchmark scIBD. Specifically, the hm-12k dataset is a mixture of human HEK293T and mouse NIH3T3 cells with 12,000 droplets, where the doublets were annotated if the barcodes were associated with both human and mouse, while the nuc-MULTI dataset is a mixture of purified nuclei from human HEK293Ts, Jurkats, and mouse embryonic fibroblasts, where the doublets were annotated by MULTI-seq [46].

To facilitate the benchmarking, we directly obtained the processed count matrices and binarized the transcript count, based on which scIBD was performed to detect doublets. scIBD can perform well on the binarized scRNA-seq data compared with the methods that were benchmarked by the review, including doubletCells [47], Scrublet [5], cxds [48], bcds [48], hybrid [48], Solo [49], DoubletDetection [50], and DoubletFinder [51] (Additional file 2: Table S5). On the hm-12k dataset with high cell heterogeneity, scRNA-seq doublet detection methods have fairly performed well, where cxds and Solo achieved the AUROC values with 1, cxds achieved the AUPRC value with 0.998, while scIBD also achieved a close performance (AUROC = 0.998 and AUORC = 0.995), surpassing the rest six methods. Importantly, on the nuc-MULTI dataset that is more complex, analog to the semi-synthetic datasets, scIBD outperformed the scRNA-seq-specific baseline methods, improved the AUROC by 2% (0.790) and AUPRC by 10% (0.487) compared with DoubletFinder that was the best-performed (AUROC = 0.775 and AUPRC = 0.441) among the baseline methods. These results suggest that scIBD is a robust and versatile tool for doublet detection across different single-cell sequencing platforms.

## Discussions

Detecting and removing the doublets in scCAS data is an essential issue in genome biology research, which facilitates and improves the downstream analyses. Challenged by the assay-specific characteristics of scCAS data, in this work, we proposed scIBD, a self-supervised iterative-optimizing method to boost the detection of heterotypic doublets in scCAS data. scIBD is a simulation-based method, where heterotypic doublets with high confidence are simulated as the reference using the pseudo-droplet-based simulation strategy. Meanwhile, by applying an iterative strategy, scIBD can iteratively and progressively detect the real heterotypic doublets based on the adaptive KNN graph. The ablation experiments that demonstrate the contributions of the iteration process and the specific doublet simulation strategy are illustrated (Additional file 3: Content S1, Additional file 1: Fig. S6). In addition, the efficacy comparisons of scIBD with the baseline methods are presented (Additional file 3: Content S2, Additional file 2: Table S6).

When benchmarking scIBD on the semi-synthetic datasets, we utilized a weighting criterion to simulate the ground-truth doublets (the “Construction of benchmark datasets” section). The main purpose is to reflect the true occurrence of heterotypic doublets in sequencing, where the probability of a cell type forming doublets depends on its proportion in the tissue [16]. To evaluate the performance on the semi-synthetic datasets without the weighting criterion, we additionally constructed two Islets datasets where the doublets are simulated by randomly picking two cell types. Except for the weighted simulation, all other simulation steps remain the same as the rigorously quality-controlled Islets datasets with the weighting criterion in Table 1, where the same potential doublets in raw data were removed in advance using ArchR and AMULET to establish high-quality singlets. The performance of the simulation-based methods (scIBD, SnapATAC, and AcrhR) all decrease on the datasets where the ground-truth doublets were simulated without the weighting criterion (Additional file 2: Table S7). However, the performance of AMULET, which is read-based, achieves nearly identical results on the datasets for that it only performs statistical analyses on the overlapped reads, regardless of the different cell type origins, also suggesting the superiority in detecting homotypic doublets. The drastic decrease in performance of the simulation-based methods may be due to the bias between the simulated doublets and ground-truth doublets, demonstrating the rationale of the weighting criterion when simulating ground-truth doublets for that ArchR and SnapATAC utilize the totally random strategy in their simulation steps. Importantly, although scIBD also achieves decreased performance on the unweighted-datasets, it still outperforms the baseline methods.

We also describe several avenues for improving and extending scIBD. First, we can use bulk and/or additional single-cell chromatin accessibility data as a reference to better characterize biological variations [13, 52, 53]. Second, we can incorporate the batch information of scCAS data when modeling to facilitate the batch effect. Third, heuristic strategies for estimating the doublet rate of a scCAS dataset are in pressing need. Finally, we can further consider the barcode multiplet problem, which is another issue in droplet-based single-cell sequencing [54]. Unlike the doublet issue discussed in this study, the barcode multiplet is referred to as one cell’s nucleic acids captured by more than one oligonucleotide barcode sequence, which also yields confusing single-cell profiles. This is also a challenging task since the barcode oligonucleotides are complex and heterogeneous.

## Conclusions

Extensive and comprehensive evaluation results on multiple datasets, including fully-synthetic, semi-synthetic, and real scCAS data, demonstrate that scIBD significantly outperforms the baseline methods, including SnapATAC, ArchR, and AMULET. Besides, more comprehensive evaluations on different scenarios show that scIBD has superior robustness to cell heterogeneity, sample size, sequencing depth, and doublet rate. The specifically designed PCoA KNN graphing strategy shows better performance on the datasets with more complex cell heterogeneity. Moreover, the downstream biological analyses, including cell clustering, differential accessible region finding, and KEGG enrichment analyses, further show the practical efficacy of doublet-removal by scIBD. At last, the extended application of scIBD on scRNA-seq data, demonstrates the robustness and versatility of scIBD for doublet detection. We confirmedly believe that scIBD will enable better quality control on removing doublets and expand the applicability of scCAS by yielding better downstream analysis results that enable a deeper understanding of cellular-level epigenomic heterogeneity and functions. scIBD is an easy-to-use open-source tool available at multiple sources and can be seamlessly integrated into existing scCAS analysis workflows.

## Methods

### Data collection and data processing

#### Data source

The scCAS data used in this study were derived from three main sources. The dataset of human mixed cell lines (HMC) was generated from a mixture of ten human cell lines using single-nucleus ATAC-seq [6]. The dataset of mouse forebrain (MF) was generated from the forebrain tissue of an 8-week-old adult mouse by single-nucleus ATAC-seq [19]. The dataset of mouse cell atlas (MCA) contains 17 samples generated from 13 different tissues of 8-week-old mice using single-cell combinatorial indexing ATAC-seq [20]. We adopted the samples in which the predominant cell type accounts for no more than 80%. The selected 11 samples were derived from eight tissues, including bone marrow, lung, whole brain, cerebellum, heart, kidney, prefrontal cortex, and spleen, among which bone marrow has differentiating blood cells that are on a continuous trajectory, and the others have distinct cell types. The two Islets datasets were generated from the primary human tissue islets of two donors, which were independently captured and sequenced using the 10x Genomics Chromium platform [16, 55]. The peripheral blood mononuclear cell (PBMC) dataset was derived from cryopreserved human peripheral blood mononuclear cells of a healthy female donor aged 25 obtained by 10x Genomics, where the cell annotations are provided by Cao et al. [21].

#### Construction of benchmark datasets

Since doublet-detection in scCAS data is a challenging task, most scCAS datasets, including the datasets of MF and MCA, do not come with annotations of doublets, highlighting the importance of convincing construction of datasets with ground-truth singlet and doublet labels to benchmark the performance of different methods. We constructed three categories of benchmark datasets: (1) fully-synthetic datasets where both singlets and ground-truth doublets are artificial; (2) real datasets where the singlets and doublets were annotated by Demuxlet [4]; (3) semi-synthetic datasets where singlets are real droplets while doublets are artificial.

For the first category, we generated singlets of different cell types by simATAC [23] based on the count matrices of corresponding cell types in the MF dataset. Then, doublets were obtained by mixing the profiles of simulated singlets. The fully-synthetic datasets were used as a proof of concept to demonstrate scIBD.

For the second category, since the HMC dataset comes with labels of cell types and doublets annotated by Demuxlet, we directly used the original datasets for benchmarking. The real datasets, though containing distinct and straightforward cell types, can be served as simple datasets with ground-truth doublet labels. However, the doublets annotated by Demuxlet are not exactly accurate, which demands more comprehensive benchmark works.

For the third category, we built datasets by manually constructing the artificial doublets using BAM/fragment files of the MF, MCA, Islets, and PBMC datasets. Importantly, we aim to stress the heterotypic doublets which significantly disturb the downstream analyses. Meanwhile, according to Thibodeau et al., the likelihood of a cell type to form multiplets is associated with its frequency within the tissue [16]. Therefore, we adopted the weighting criterion to generate ground-truth doublets for comprehensive benchmarking on semi-synthetic datasets. We first selected two cell types with the probability proportional to the cell type proportions in the dataset. For each one of the selected cell types, we then randomly picked one droplet among the cell type. According to the read statistics based on the HMC real datasets (Additional file 1: Fig. S7), the sequencing depth of doublets is significantly higher than that of singlets. We thus directly mixed the total reads of the two picked droplets to form a doublet. Note that for the MF dataset and the three tissues (bone marrow, lung and whole brain) in the MCA dataset that have two replicates, we used the droplets in one replicate to construct doublets and used the droplets in another replicate to form singlets. For the remaining five tissues in the MCA dataset that have only one replicate, we directly mixed the droplets to construct doublets. The detailed pipeline for constructing doublets in semi-synthetic datasets is illustrated in Additional file 1: Fig. S8. Hereafter, we use the tissue identities to indicate the semi-synthetic datasets.

#### Data preprocessing

The input of scIBD is a scCAS count matrix. Following the pipeline introduced by Preissl et al. [19], we performed integral peak calling using MACS3 [56] with default parameters directly based on the BAM file of a dataset and subsequently constructed the count matrix with the called peaks for the droplets. To reduce the noise level, we selected peaks that are open in at least 1% of the droplets in the count matrix [13, 14] and obtained a droplet-by-peak count matrix $\mathbf{X}\in {\mathbb{R}}^{N\times P}$. The term frequency-inverse document frequency (TF-IDF) transformation was then performed to normalize the count matrix [13, 14], as described as follows:

1 $${X}_{i,j}^{\mathrm{^{\prime}}}= \frac{{X}_{i,j}}{{\sum }_{p=0}^{P}{X}_{i, p}}\times log\frac{|1+P|}{{\sum }_{n=0}^{N}{X}_{n, j}},$$

### Self-supervised iterative-optimizing KNN in scIBD

#### Simulation of high-confident artificial heterotypic doublets

Existing simulation-based methods take the routine that generally picks two random droplets from all droplets in the scCAS dataset to simulate a doublet, yielding a bunch of homotypic artificial doublets. Besides, existing methods only simulate artificial doublets once, ignoring that not all original droplets are singlets. The above drawbacks result in low-quality training data for the binary classifier. Therefore, we aim to simulate more heterotypic doublets in a way that resembles their natural formation, which is called pseudo-droplet-based simulation. Current state-of-the-art clustering methods for single-cell data like Louvain and Leiden tend to split a predominant cell type into several subtypes to find more communities (Additional file 1: Fig. S9). We hereby adopted a clustering strategy that reserves the predominant cell types in clustering results, based on which artificial heterotypic doublets of high confidence can be subsequently simulated, avoiding too many homotypic ones that may obscure detecting heterotypic doublets. The clustering process can be described as follows:

2 $$\mathbf{C}=\mathrm{DBSCAN}\left(\mathrm{UMAP }\left(\mathrm{PCA }\left(\mathbf{X}\mathrm{^{\prime}}\right)\right)\right),$$

where $\mathbf{X}\mathrm{^{\prime}}\in {\mathbb{R}}^{N\times P}$ is the TF-IDF transformed matrix, principal component analysis (PCA) is canonically performed to reduce dimensionality, followed by uniform manifold approximation and projection (UMAP) [57, 58], and the density-based DBSCAN clustering method [59] is finally applied to obtain the clustering results $\mathbf{C}\in {\mathbb{R}}^{N\times 1}$ with the empirically adjusted parameters. Specifically, under the assumption that clusters are dense regions in space separated by regions of lower density, DBSCAN assigns the droplets that are “densely grouped” the same cluster label. Therefore, the hyper-parameters involved in this clustering pipeline to control the cluster results are “eps” and “min_samples,” representing the maximum distance between two droplets for one to be considered as in the neighborhood of the other, and the number of samples in a neighborhood for a droplet to be considered as a core droplet, respectively. In our specific clustering strategy, the parameter “eps” is set as 0.1 and the parameter “min_samples” is self-adaptively set as 0.5% of the number of input droplets, which are consistently used in all benchmarking experiments.

Weighted by the sizes of clusters, two clusters are randomly selected, namely, the larger size the cluster has, the more likely the cluster is selected. For each one of the selected clusters, scIBD randomly picks one droplet and then takes the union of their count profiles to form an artificial doublet. Artificial heterotypic doublets are generated independently via the above strategy. Apart from the specially designed doublets, scIBD also generates a portion of doublets by the canonical strategy that randomly picks two cells. For each iteration, the number of all generated artificial doublets equals the downward integer of 30% of the number of droplets that have not been detected as doublets, among which 70% are the specially designed artificial heterotypic doublets, and 30% are the randomly picked ones. scIBD can thus update the artificial doublets in each iteration and self-supervise for doublet-detection.

#### Construction of KNN graph

Based on the droplets and the generated artificial doublets, KNN graph is constructed to detect potential doublets in each iteration. We provide two strategies to construct the KNN graph, referred to as PCA-based and principal coordinates analysis (PCoA)-based graphing, respectively. In PCA-based graphing, the KNN graph is constructed based on the embedding obtained by PCA and UMAP, while in PCoA-based graphing, we calculate Jaccard distance based on the count matrix and then perform the PCoA analysis to obtain the embeddings. The KNN graph is then constructed using the ANNOY package (https://github.com/spotify/annoy), which is almost as fast as the fastest libraries to do nearest-neighbor search and is dedicated to minimizing memory footprint.

**Fig. 8:**
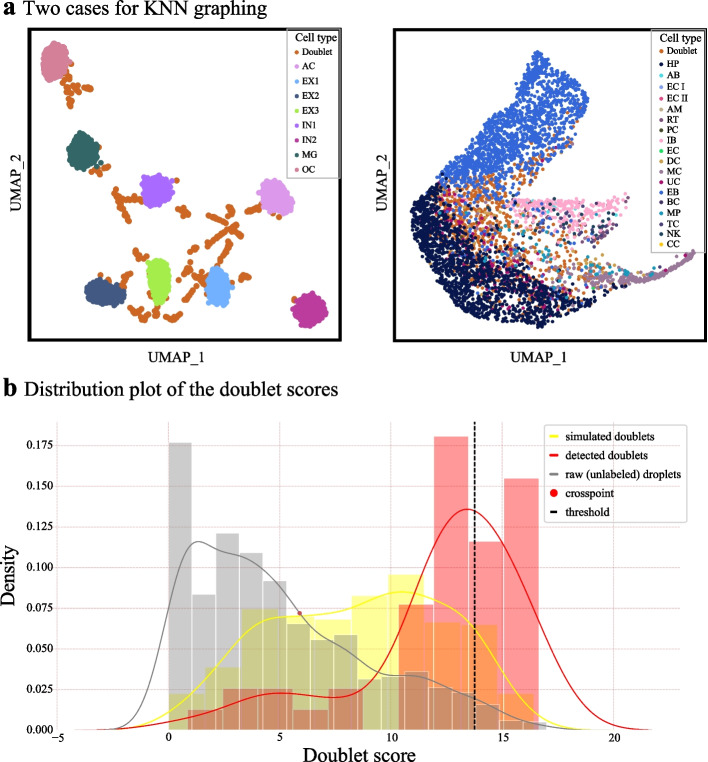
The specific strategies applied in scIBD. **a** Two cases that are suitable for different KNN-graphing strategies. Left panel illustrates the case where different cell types are distinguishable in UMAP based on PCA embeddings, and the doublets are also distinctly apart from the singlets, PCA-based graphing is applied. Right panel illustrates the case where the distinction of the cells is not clear by following PCA-based strategy, PCoA-based graphing is applied to further separate the doublets from singlets. **b** The distribution plot of the doublet scores during the iteration process. We separately show the doublet score distributions of three parts, the detected doublets in former iterations, the simulated doublets in each iteration, and the unlabeled droplets in raw sets. In each iteration, we aim at separating doublets from the unlabeled droplets. The doublet scores of the unlabeled droplets are modeled by the right side of a standard Gaussian. The scores of the simulated doublets (yellow) are used as the reference to obtain the threshold to determine the doublets in the unlabeled droplets. The scores of the doublets detected in former iterations (red) mostly locate at high intervals, showing their high confidence as the doublets

scIBD can adaptively opt a KNN graphing strategy that suits the input. As shown in Fig. 8a left panel, if the cell types are distinguishable, i.e., the cell types have remarkable patterns in accessible profiles, it is efficient to apply the PCA-based graphing since the principal components are obtained based on the variance difference. However, in such cases, PCoA, which is performed based on Jaccard distance matrix, may eliminate the distinction in accessible patterns. On the contrary, if cell types cannot be separated readily through PCA analysis (Fig. 8a right panel), PCoA has superiority in distinguishing doublets and singlets whose differences are not solid enough, since it tries to reserve the distance relationship between the droplets.

To automatically determine the choice of KNN graphing strategy, scIBD first performs DBSCAN clustering and then uses Calinski-Harabasz index (CHI) [60] to assess the ratio of the sum of between-cluster dispersion and of within-cluster dispersion. The calculation of CHI is defined as follows:

3 $$\mathrm{CHI}= \frac{\mathbf{B} \left(N-K\right)}{\mathbf{W} \left(K-1\right)},$$

where $N$ is the total number of droplets, $K$ is the number of clusters, $\mathbf{B}$ is the inter-class covariance across all clusters, indicating the overall dispersion between clusters, and $\mathbf{W}$ is the summed-up intra-class covariance within clusters, indicating the overall compactness of clusters. The higher the CHI is, the better the clustering performs. Based on the CHI scores ${\mathrm{CHI}}_{\mathrm{PCA}}$ and ${\mathrm{CHI}}_{\mathrm{PCoA}}$ of PCA-based and PCoA-based graphing and the understanding that if the cell types are easy to distinguish, the ratio of ${\mathrm{CHI}}_{\mathrm{PCoA}}$ and ${\mathrm{CHI}}_{\mathrm{PCA}}$ is supposed to be little, and vice versa, we set a ratio threshold of 1.5 which is empirically adjusted and scIBD will adaptively choose the PCA-based graphing when the ratio is less than the threshold and choose the PCoA-based graphing otherwise.

#### Calculation of doublet score

After constructing the KNN graph, we can obtain a matrix containing the distance between each of the $N$ droplets and its $K$ nearest neighbors, denoted as:

4 $$\mathbf{D}= \left[\begin{array}{ccc}{d}_{\left(1,{nn}_{1}\right)}& {d}_{\left(1,{nn}_{2}\right)}& {d}_{\left(1,{nn}_{K}\right)}\\ {d}_{\left(2,{nn}_{1}\right)}& \dots & {d}_{\left(2,{nn}_{K}\right)}\\ {d}_{\left(N,{nn}_{1}\right)}& {d}_{\left(2,{nn}_{2}\right)}& {d}_{\left(N,{nn}_{K}\right)}\end{array}\right] \in {\mathbb{R}}^{N\times K},$$

where ${d}_{\left(i,{nn}_{j}\right)}$ is the distance between droplet $i$ and its $j$-th nearest neighbor. We then perform a reversed min-max normalization to scale the distance matrix as the similarity matrix:

5 $$\mathbf{D}^{\prime}= \frac{max\left(\mathbf{D}\right) - \mathbf{D}}{ max\left(\mathbf{D}\right) - min\left(\mathbf{D}\right)} \in {\mathbb{R}}^{N\times K}.$$

Meanwhile, we can also obtain a reference doublet score matrix based on the neighbors and their referred doublets scores from the iteratively updated doublet score reference vector $\mathbf{L}$, denoted as:

6 $$\mathbf{R}=\left[\begin{array}{ccc}{l}_{\left(1,{nn}_{1}\right)}& {l}_{\left(1,{nn}_{2}\right)}& {l}_{\left(1,{nn}_{K}\right)}\\ {l}_{\left(2,{nn}_{1}\right)}& \dots & {l}_{\left(2,{nn}_{K}\right)}\\ {l}_{\left(N,{nn}_{1}\right)}& {l}_{\left(2,{nn}_{2}\right)}& {l}_{\left(N,{nn}_{K}\right)}\end{array}\right]\in {\mathbb{R}}^{N\times K},$$

where ${l}_{\left(i,{nn}_{j}\right)}$ is the doublet score of droplet $i$’s $j$-th nearest neighbor referred from $\mathbf{L}$. Specifically, in the first iteration, ${l}_{\left(i,{nn}_{j}\right)}=1$ only if the $j$-th nearest neighbor cell of droplet $i$ is the simulated doublet; otherwise, 0. ${l}_{\left(i,{nn}_{j}\right)}$ in $\mathbf{L}$ will be updated in each iteration via aggregating the reference doublet scores of KNN of each droplet weighted by their distance, and thus $\mathbf{R}$ will also be altered even if the KNN graph structure remains the same. Based on the KNN graph in each iteration, the doublet scores for all droplets (all the raw droplets and the simulated and detected doublets, not limited to the nearest neighbors, i.e., $\mathbf{S}=\left({\mathbf{S}}_{raw},{\mathbf{S}}_{sim}\right)$, can then be calculated as follows:

7 $$\mathbf{S}=\sum\nolimits_{k=1}^{K}{[{\mathbf{D}}^{\mathrm{^{\prime}}}\circ \mathbf{R}]}^{T},$$

where $\circ$ indicates the element-wise multiplication.

#### Iterative-optimizing detection of doublets

The iterative process for detecting doublets is illustrated in Fig. 1b. In each iteration, the doublets scores for all droplets, ${\mathbf{S}}_{raw}$, are calculated based on the KNN structure and the $\mathbf{L}$ updated by the previous iteration. Note that ${\mathbf{S}}_{raw}$ is composed of scores of singlets and undetected doublets, while ${\mathbf{S}}_{sim}$ are the scores of newly simulated doublets in each iteration. Among the raw droplets, most are singlets with a doublet score of zero, and doublets only account for a small portion of all the raw droplets (Fig. 8b). Therefore, the doublet scores of the droplets can be fitted as the “right side of a standard Gaussian distribution,” i.e., the number of droplets declines with the increase of doublet scores and no droplet has negative scores. We use the right side of a standard Gaussian to model ${\mathbf{S}}_{raw}$ to describe the doublet score distribution of raw droplets, and a Gaussian to model ${\mathbf{S}}_{sim}$. A doublet-detection threshold ${s}_{th}$ based on the two distributions is calculated as follows:

8 $${s}_{th}={o(p(\mathbf{S}}_{raw}), p({\mathbf{S}}_{sim}))+2std({\mathbf{S}}_{sim}),$$

where ${o(p(\mathbf{S}}_{raw}), p({\mathbf{S}}_{sim}))$ is the crossing point of the two density curves of ${\mathbf{S}}_{raw}$ and ${\mathbf{S}}_{sim}$, $std\left({\mathbf{S}}_{sim}\right)$ is the standard variance of ${\mathbf{S}}_{sim}$, and $2std$ indicates about the 95% confidence interval. The distribution plot is illustrated in Fig. 8b.

The droplets whose doublet scores are higher than ${s}_{th}$ are detected as doublets, denoted as $\mathbf{D}=\left({d}_{1}, {d}_{2}\dots {d}_{i}\right)$. These detected doublets no longer participate in the following clustering and simulating steps but participate in the KNN aggregation with their inferred doublet scores. Meanwhile, the unlabeled set, where the noisy doublets have been removed, is expected to obtain better clustering results, yielding higher-quality simulated heterotypic doublets. In each iteration, the doublet scores of the detected doublets will be scaled between 0.1 and 0.9, to distinguish them from the undetected droplets whose doublet scores are 0 and the simulated doublets whose scores are 1, and then their scaled doublet scores will be used to update $\mathbf{L}$, which is used as the reference in subsequent iterations.

The iteration process is supposed to stop when the detected doublets reach the number of the expected doublet rate or no more unlabeled droplet has a higher doublet score than ${s}_{th}$. However, the iteration process sometimes stops early if the expected doublet rate is too low or the doublets are easy to detect. To slightly prolong the iterations, we also adopt an annealing strategy at the ending criterion of the iterative process. Specifically, we set $p$ (initially is 1) to indicate the probability that the iteration continues. Only when the algorithm reaches the supposed ending criterion, the current iteration continues by probability $p$ and $p$ starts to decay along with the model iteration. The more iterations scIBD has processed, the more rapidly $p$ decays, i.e., the iteration process tends to continue when it stops early. Finally, upon the iteration stops, the doublets scores of droplets across all iterations will be averaged and then scaled between 0 and 1 to obtain the final scores. Providing the final doublet scores will also enhance the usability of scIBD, since users can then have the flexibility to decide the exact number of doublets to be detected based on their preference. The pseudocode of the iterative doublet detection process in scIBD is described in Additional file 3: Content S3.

### Baseline methods

We benchmarked the performance of scIBD with SnapATAC [15], ArchR [6], and AMULET [16]. SnapATAC integrates Scrublet [5], a model designed for scRNA-seq, to detect doublets in scCAS data. We performed SnapATAC for doublet-detection using the same count matrices input to scIBD. ArchR and AMULET can only be performed using BAM/fragment files, thereby we only benchmarked the performance of scIBD on real datasets and semi-synthetic datasets which have BAM/fragment files. All baseline methods were performed using their default parameters. Specifically, the parameter of expected doublet rate can influence the performance of the baseline methods, we thus set it as the ground-truth doublet rate in the benchmark dataset for all methods to pursue justified comparisons.

### Metrics for evaluation

We first evaluated the accuracy of doublet-detection results based on the area under receiver operator characteristic curve (AUROC) and the area under precision-recall curve (AUPRC). Precision, recall, and F1 scores are also used to show more explicit comparisons. In addition, considering that doublets can confound the downstream analyses, especially the clustering, we further performed the widely used Louvain algorithm [61] on the doublet-removed datasets and evaluated the clustering results by adjusted Rand index (ARI) and adjusted mutual information (AMI). The detailed descriptions of the evaluation metrics are given in Additional file 3: Content S4.

### Supplementary Information

**Additional file 1:** **Fig. S1.** The impact of homotypic and heterotypic doublets. We used the simulated data to illustrate the impact of homotypic and heterotypic doublets. We first used one replicate of forebrain from MF that contains eight annotated cell types, to generate the count matrix. The count matrix of single cell type was then used to separately generate corresponding fully-synthetic data by simATAC. Here, we generated the data of different cell types with equal size. The homotypic doublets are generated by picking two simulated singlets from the same cell type and then mixing their profiles. The heterotypic doublets are generated by picking two simulated singlets from different cell types and then mixing their profiles. As is clearly shown, the heterotypic doublets may confound the downstream analysis more seriously than the homotypic doublets. Therefore, we stressed the heterotypic doublet detection in this work. **Fig. S2.** The UMAP of the semi-synthetic benchmark datasets to understand the complexity. The TF-IDF transformation was firstly performed on the count matrices, PCA and UMAP were subsequently performed on the transferred matrices using the default parameters in EpiScanpy. **Fig. S3.** The illustration of the impact of doublets and the efficacy of doublet-removal by scIBD on the imbalanced fully-synthetic dataset where the sizes of different cell types are different. We first visualized the simulated singlets, which show great heterogeneity between different cell types as expected. After adding the simulated doublets, we can clearly observe that the doublets scatter between the cell types, resulting in spurious clustering results. The heatmap of doublet scores provided by scIBD is presented, where the droplets with high doublet scores are fairly consistent with the ground-truth doublets. To further illustrate the efficacy of scIBD in clustering, we removed the doublets called by scIBD. As clearly shown, the clusters reveal significant heterogeneity without the detected doublets, and the number of clusters obtained by default parameters is exactly equal to that of true cell types in the ground truth, showing the improvement of clustering by scIBD. Although some doublets failed to be detected, the remained doublets mostly lay at the edge of main cell types, and have negligible impact on the clustering results. **Fig. S4.** The receiver operating characteristic (ROC) curves and precision-recall (PR) curves of scIBD and the baseline methods on the benchmark datasets. **Fig. S5.** The performance evaluation on the semi-synthetic datasets where the doublets have different numbers of captured reads. a The AUROC (solid lines) and the AUPRC (dotted lines) show the trend of performance with the reads decrease of doublets on the rest five semi-synthetic datasets. b The critical difference diagrams (Wilcoxon signed-rank test with Holm’s alpha (5% two-sided) correction) of AUROC and AUPRC under different reads down-sampling rates over all the nine semi-synthetic datasets. The results show that scIBD achieves the best overall performance across all the semi-synthetic datasets. **Fig. S6.** Ablation experiments. a The iterative-optimizing process. The AUROC and AUPRC of each iteration are illustrated in the plots, where the bottom bar represents the metrics calculated based on the doublet scores from the current iteration, while the top bar represents the metrics calculated based on the mean scores across all iterations former till the current one. b The performance boosted by our specific simulation strategy. The performance comparison between our proposed strategy and the traditional random strategy for simulating doublets. **Fig. S7.** The sequencing depth statistics based on the HMC datasets. The sequencing depth of doublets is significantly higher than that of singlets. **Fig. S8.** The pipeline of constructing ground-truth doublets in the semi-synthetic datasets. We mixed all the reads of two selected cells to construct a ground-truth doublet based on the BAM files. The raw BAM file of singlets and the generated BAM file of ground-truth doublets are concatenated as a complete BAM file for the subsequent process. The cells that are selected to construct benchmark doublets are weighted by the known cell annotations. Specifically, we obtained the proportions of all cell types based on their known annotations; according to the cell type proportions we then probabilistically selected two types, from which one cell was respectively randomly selected to form a heterotypic doublet. This simulation pipeline was repeatedly performed to simulate all ground-truth heterotypic doublets. **Fig. S9.** The difference of clustering between Leiden and scIBD. Using ground-truth annotations as the reference, Leiden tends to split the predominant cell class into several subclasses, thus yielding low-quality simulated doublets. scIBD applies a specific clustering pipeline that can reserve the main cell type, especially in an extremely-imbalanced dataset.

**Additional file 2:** **Table S1.** The detailed performance metrics under the given doublet truncating rate of different methods based on their respective doublet scores.**Table S2.** Performance comparison on the Islets (Islet1 and Islet2) and PBMC datasets. **Table S3.** The number of overlapped differential accessible peaks (microglia vs. rest) between the doublets removed (RM) datasets by different methods and the ground truth (singlets). **Table S4.** Top 20 differential accessible peaks of the microglia cluster on the ground truth (GT), doublets retaining (RE), and doublets removal (RM) datasets. **Table S5.** The performance comparison between scIBD and the baseline methods that are designed for doublet-detection in scRNA-seq data. **Table S6.** The computational efficacy comparison of scIBD and the baseline methods. **Table S7.** Performance comparison on Islets datasets where the doublets are simulated with and without weighting criteria respectively.(DOCX 2433 KB)

**Additional file 3:** **Content S1.** Ablation experiments. **Content S2.** Computational efficacy comparison.**Content S3.** Pseudocode. **Content S4.** Metrics for evaluation.

**Additional file 4.** Review history.

## Data Availability

scIBD is implemented as Python packages, which is freely available under the MIT license on Github: https://github.com/Ying-Lab/scIBD [62] and Zenodo: 10.5281/zenodo.8207525 [63], with detailed documentation at https://scibd.readthedocs.io [64]. Human Mixed Cell lines (HMC) data were obtained from the study with GEO accession GSE162690 [6]. Mouse forebrain (MF) data were obtained from the study with GEO accession GSE100033 [19]. Mouse Cell Atlas (MCA) data were obtained from the study with GEO accession GSE111586 [20]. The Islets data were downloaded from the study with GEO accession GSE165212 [16]. The PBMC dataset along with the cell type annotations were obtained from https://github.com/gao-lab/GLUE/tree/master/data/download/10x-Multiome-Pbmc10k [21, 22]. The scRNA-seq data are downloaded from http://zenodo.org/record/4562782 [7]. The source code and pre-processing scripts are available under the MIT license on Github http://github.com/Ying-Lab/scIBD [62]. scIBD along with detailed documentation is freely accessible at http://scibd.readthedocs.io [64].

## References

[1] Stuart T, Satija R (2019). Integrative single-cell analysis. Nat Rev Genetics.

[2] Zilionis R, Nainys J, Veres A, Savova V, Zemmour D, Klein AM, Mazutis L (2017). Single-cell barcoding and sequencing using droplet microfluidics. Nat Protocols.

[3] Guo MT, Rotem A, Heyman JA, Weitz DA (2012). Droplet microfluidics for high-throughput biological assays. Lab Chip.

[4] Kang HM, Subramaniam M, Targ S, Nguyen M, Maliskova L, McCarthy E, Wan E, Wong S, Byrnes L, Lanata CM (2020). Multiplexed droplet single-cell RNA-sequencing using natural genetic variation (vol 36, pg 89, 2018). Nat Biotechnol.

[5] Wolock SL, Lopez R, Klein AM (2019). Scrublet: computational identification of cell doublets in single-cell transcriptomic data. Cell Syst.

[6] Granja JM, Corces MR, Pierce SE, Bagdatli ST, Choudhry H, Chang HWY, Greenleaf WJ (2021). ArchR is a scalable software package for integrative single-cell chromatin accessibility analysis (vol 53, pg 403, 2021). Nat Genet.

[7] Xi NM, Li JJ (2021). Benchmarking computational doublet-detection methods for single-cell RNA sequencing data. Cell Systems.

[8] Tang FC, Barbacioru C, Wang YZ, Nordman E, Lee C, Xu NL, Wang XH, Bodeau J, Tuch BB, Siddiqui A (2009). mRNA-Seq whole-transcriptome analysis of a single cell. Nat Methods.

[9] Potter SS (2018). Single-cell RNA sequencing for the study of development, physiology and disease. Nat Rev Nephrol.

[10] Stoeckius M, Zheng SW, Houck-Loomis B, Hao S, Yeung BZ, Mauck WM, Smibert P, Satija R (2018). Cell Hashing with barcoded antibodies enables multiplexing and doublet detection for single cell genomics. Genome Biol.

[11] Buenostro JD, Wu BJ, Litzenburger UM, Ruff D, Gonzales ML, Snyder MP, Chang HY, Greenleaf WJ (2015). Single-cell chromatin accessibility reveals principles of regulatory variation. Nature.

[12] Chen H, Lareau C, Andreani T, Vinyard ME, Garcia SP, Clement K, Andrade-Navarro MA, Buenrostro JD, Pinello L (2019). Assessment of computational methods for the analysis of single-cell ATAC-seq data. Genome Biol.

[13] Chen SQ, Yan GA, Zhang WY, Li JZ, Jiang R, Lin ZX (2021). RA3 is a reference-guided approach for epigenetic characterization of single cells. Nat Commun.

[14] Chen XY, Chen SQ, Song S, Gao ZJ, Hou L, Zhang XG, Lv HR, Jiang R (2022). Cell type annotation of single-cell chromatin accessibility data via supervised Bayesian embedding. Nat Machine Intell.

[15] Fang RX, Preissl S, Li Y, Hou XM, Lucero J, Wang XX, Motamedi A, Shiau AK, Zhou XZ, Xie FM (2021). Comprehensive analysis of single cell ATAC-seq data with SnapATAC. Nat Commun.

[16] Thibodeau A, Eroglu A, McGinnis CS, Lawlor N, Nehar-Belaid D, Kursawe R, Marches R, Conrad DN, Kuchel GA, Gartner ZJ (2021). AMULET: a novel read count-based method for effective multiplet detection from single nucleus ATAC-seq data. Genome Biol.

[17] Nettleton DF, Orriols-Puig A, Fornells A (2010). A study of the effect of different types of noise on the precision of supervised learning techniques. Artif Intell Rev.

[18] Danese A, Richter ML, Chaichoompu K, Fischer DS, Theis FJ, Colome-Tatche M (2021). EpiScanpy: integrated single-cell epigenomic analysis. Nat Commun.

[19] Preissl S, Fang RX, Huang H, Zhao Y, Raviram R, Gorkin DU, Zhang YX, Sos BC, Afzal V, Dickel DE (2018). Single-nucleus analysis of accessible chromatin in developing mouse forebrain reveals cell-type-specific transcriptional regulation (vol 21, pg 432, 2018). Nat Neurosci.

[20] Cusanovich DA, Hill AJ, Aghamirzaie D, Daza RM, Pliner HA, Berletch JB, Filippova GN, Huang XF, Christiansen L, DeWitt WS (2018). A single-cell atlas of in vivo mammalian chromatin accessibility. Cell.

[21] Cao ZJ, Gao G (2022). Multi-omics single-cell data integration and regulatory inference with graph-linked embedding. Nat Biotechnol.

[22] Genomics X (2020). PBMC from a healthy donor, single cell multiome ATAC gene expression demonstration data by Cell Ranger ARC 1.0.0.

[23] Navidi Z, Zhang L, Wang B (2021). simATAC: a single-cell ATAC-seq simulation framework. Genome Biol.

[24] Branco P, Torgo L, Ribeiro RP (2016). A survey of predictive modeling on imbalanced domains. ACM Comput Surv (CSUR).

[25] Fawaz HI, Forestier G, Weber J, Idoumghar L, Muller PA (2019). Deep learning for time series classification: a review. Data Mining Knowledge Discov.

[26] Stuart T, Srivastava A, Madad S, Lareau CA, Satija R (2021). Single-cell chromatin state analysis with Signac. Nat Methods.

[27] Chen S, Wang R, Long W, Jiang R (2023). ASTER: accurately estimating the number of cell types in single-cell chromatin accessibility data. Bioinformatics.

[28] Ginhoux F, Lim S, Hoeffel G, Low D, Huber T (2013). Origin and differentiation of microglia. Front Cell Neurosci.

[29] Gehrmann J, Matsumoto Y, Kreutzberg GW (1995). Microglia - intrinsic immuneffector cell of the brain. Brain Res Rev.

[30] Agarwala R, Barrett T, Beck J, Benson DA, Bollin C, Bolton E, Bourexis D, Brister JR, Bryant SH, Canese K (2018). Database resources of the National Center for Biotechnology Information. Nucleic Acids Res.

[31] Lee WH, Higuchi H, Ikeda S, Macke EL, Takimoto T, Pattnaik BR, Liu C, Chu LF, Siepka SM, Krentz KJ (2016). Mouse Tmem135 mutation reveals a mechanism involving mitochondrial dynamics that leads to age-dependent retinal pathologies. Elife.

[32] Lee WH, Bhute VJ, Higuchi H, Ikeda S, Palecek SP, Ikeda A (2020). Metabolic alterations caused by the mutation and overexpression of the Tmem135 gene. Exp Biol Med (Maywood).

[33] Kamphuis W, Kooijman L, Schetters S, Orre M, Hol EM (2016). Transcriptional profiling of CD11c-positive microglia accumulating around amyloid plaques in a mouse model for Alzheimer’s disease. Biochim Biophys Acta.

[34] Ayata P, Badimon A, Strasburger HJ, Duff MK, Montgomery SE, Loh YE, Ebert A, Pimenova AA, Ramirez BR, Chan AT (2018). Epigenetic regulation of brain region-specific microglia clearance activity. Nat Neurosci.

[35] Song X, Ma FL, Herrupu O (2019). Accumulation of cytoplasmic DNA due to ATM deficiency activates the microglial viral response system with neurotoxic consequences. J Neurosci.

[36] Wu ZB, Qiu C, Zhang AL, Cai L, Lin SJ, Yao Y, et al. Glioma-associated antigen HEATR1 induces functional cytotoxic T lymphocytes in patients with glioma. J Immunol Res. 2014;2014:131494. 10.1155/2014/131494. 10.1155/2014/131494 PMC4121097 25126583

[37] Schetters STT, Gomez-Nicola D, Garcia-Vallejo JJ, Van Kooyk Y (1905). Neuroinflammation: microglia and T cells get ready to tango. Front Immunol.

[38] Solleiro Villavicencio H, Rivas Arancibia S (2018). Effect of chronic oxidative stress on neuroinflammatory response mediated by CD4+T cells in neurodegenerative diseases. Front Cell Neurosci.

[39] Raudvere U, Kolberg L, Kuzmin I, Arak T, Adler P, Peterson H, Vilo J (2019). g:Profiler: a web server for functional enrichment analysis and conversions of gene lists (2019 update). Nucleic Acids Res.

[40] Tambuyzer BR, Ponsaerts P, Nouwen EJ (2009). Microglia: gatekeepers of central nervous system immunology. J Leukocyte Biol.

[41] Khayer N, Mirzaie M, Marashi S-A, Jalessi M (2020). Rps27a might act as a controller of microglia activation in triggering neurodegenerative diseases. Plos One.

[42] Wang H, Li YP, Ryder JW, Hole JT, Ebert PJ, Airey DC, Qian HR, Logsdon B, Fisher A, Ahmed Z (2018). Genome-wide RNAseq study of the molecular mechanisms underlying microglia activation in response to pathological tau perturbation in the rTg4510 tau transgenic animal model. Mol Neurodegener.

[43] Zhang L, Li YJ, Wu XY, Hong Z, Wei WS (2015). Micro RNA-181c negatively regulates the inflammatory response in oxygen-glucose-deprived microglia by targeting Toll-like receptor 4. J Neurochem.

[44] Ransohoff RM, Perry VH (2009). Microglial physiology: unique stimuli, specialized responses. Ann Rev Immunol.

[45] Arnoux I, Audinat E. Fractalkine signaling and microglia functions in the developing brain. Neural Plast. 2015;2015:689404. 10.1155/2015/689404. 10.1155/2015/689404 PMC4539507 26347402

[46] McGinnis CS, Patterson DM, Winkler J, Conrad DN, Hein MY, Srivastava V, Hu JL, Murrow LM, Weissman JS, Werb Z (2019). MULTI-seq: sample multiplexing for single-cell RNA sequencing using lipid-tagged indices. Nat Methods.

[47] Lun ATL, McCarthy DJ, Marioni JC. A step-by-step workflow for low-level analysis of single-cell RNA-seq data with Bioconductor [version 2; peer review: 3 approved, 2 approved with reservations]. F1000Res 2016;5:2122. 10.12688/f1000research.9501.2. 10.12688/f1000research.9501.1 PMC5112579 27909575

[48] Bais AS, Kostka D (2020). scds: computational annotation of doublets in single-cell RNA sequencing data. Bioinformatics.

[49] Bernstein NJ, Fong NL, Lam I, Roy MA, Hendrickson DG, Kelley DR (2020). Solo: Doublet identification in single-cell RNA-Seq via semi-supervised deep learning. Cell Syst.

[50] Gayoso A, Shor J: GitHub: DoubletDetection. Zenodo 2019.

[51] McGinnis CS, Murrow LM, Gartner ZJ (2019). DoubletFinder: doublet detection in single-cell RNA sequencing data using artificial nearest neighbors. Cell Syst.

[52] Chen SQ, Zhang BH, Chen XY, Zhang XG, Jiang R (2021). stPlus: a reference-based method for the accurate enhancement of spatial transcriptomics. Bioinformatics.

[53] Chen SQ, Liu Q, Cui XJ, Feng ZY, Li CQ, Wang XW, Zhang XG, Wang Y, Jiang R (2021). OpenAnnotate: a web server to annotate the chromatin accessibility of genomic regions. Nucleic Acids Res.

[54] Lareau CA, Ma S, Duarte FM, Buenrostro JD (2020). Inference and effects of barcode multiplets in droplet-based single-cell assays. Nat Commun.

[55] Satpathy AT, Granja JM, Yost KE, Qi Y, Meschi F, McDermott GP, Olsen BN, Mumbach MR, Pierce SE, Corces MR (2019). Massively parallel single-cell chromatin landscapes of human immune cell development and intratumoral T cell exhaustion. Nat Biotechnol.

[56] Zhang Y, Liu T, Meyer CA, Eeckhoute J, Johnson DS, Bernstein BE, Nussbaum C, Myers RM, Brown M, Li W, Liu XS (2008). Model-based analysis of ChIP-Seq (MACS). Genome Biol.

[57] Becht E, McInnes L, Healy J, Dutertre CA, Kwok IWH, Ng LG, Ginhoux F, Newell EW (2019). Dimensionality reduction for visualizing single-cell data using UMAP. Nat Biotechnol.

[58] McInnes L, Healy J, Melville J. UMAP: uniform manifold approximation and projection for dimension reduction. 2018. Preprint at arXiv 10.48550/arXiv.1802.03426.

[59] Ester M, Kriegel H-P, Sander J, Xu X (1996). A density-based algorithm for discovering clusters in large spatial databases with noise. kdd.

[60] Caliński T, Harabasz J (1974). A dendrite method for cluster analysis. Commun Stat-theory Methods.

[61] Blondel VD, Guillaume JL, Lambiotte R, Lefebvre E (2008). Fast unfolding of communities in large networks. J Stat Mech Theory Exp.

[62] Zhang W, Jiang R, Chen S, Wang Y. scIBD. Github. 2023. https://github.com/Ying-Lab/scIBD.

[63] Zhang W, Jiang R, Chen S, Wang Y. scIBD. Zenodo. 2023. 10.5281/zenodo.8207525.

[64] Zhang W, Jiang R, Chen S, Wang Y. scIBD. 2023. https://scibd.readthedocs.io.

