# Supplementary material for: scIBD: a self-supervised iterative-optimizing model for boosting the detection of heterotypic doublets in single-cell chromatin accessibility data
Source: Genome Biol. 2023 Oct 9;24:225. doi: 10.1186/s13059-023-03072-y (PMC10561408; doi:10.1186/s13059-023-03072-y)
Supplement: Supplementary file 1 — Additional file 1: Fig. S1. The impact of homotypic and heterotypic doublets. We used the simulated data to illustrate the impact of homotypic and heterotypic doublets. We first used one replicate of forebrain from MF that contains eight annotated cell types, to generate the count matrix. The count matrix of single cell type was then used to separately generate corresponding fully-synthetic data by simATAC. Here, we generated the data of different cell types with equal size. The homotypic doublets are generated by picking two simulated singlets from the same cell type and then mixing their profiles. The heterotypic doublets are generated by picking two simulated singlets from different cell types and then mixing their profiles. As is clearly shown, the heterotypic doublets may confound the downstream analysis more seriously than the homotypic doublets. Therefore, we stressed the heterotypic doublet detection in this work. Fig. S2. The UMAP of the semi-synthetic benchmark datasets to understand the complexity. The TF-IDF transformation was firstly performed on the count matrices, PCA and UMAP were subsequently performed on the transferred matrices using the default parameters in EpiScanpy. Fig. S3. The illustration of the impact of doublets and the efficacy of doublet-removal by scIBD on the imbalanced fully-synthetic dataset where the sizes of different cell types are different. We first visualized the simulated singlets, which show great heterogeneity between different cell types as expected. After adding the simulated doublets, we can clearly observe that the doublets scatter between the cell types, resulting in spurious clustering results. The heatmap of doublet scores provided by scIBD is presented, where the droplets with high doublet scores are fairly consistent with the ground-truth doublets. To further illustrate the efficacy of scIBD in clustering, we removed the doublets called by scIBD. As clearly shown, the clusters reveal significant heterogeneity witho [file 13059_2023_3072_MOESM1_ESM.docx]

**scIBD: a self-supervised iterative-optimizing model for boosting the detection of heterotypic doublets in single-cell chromatin accessibility data**

Additional file 1: Supplementary figures

Wenhao Zhang, Rui Jiang, Shengquan Chen^*^ and Ying Wang*


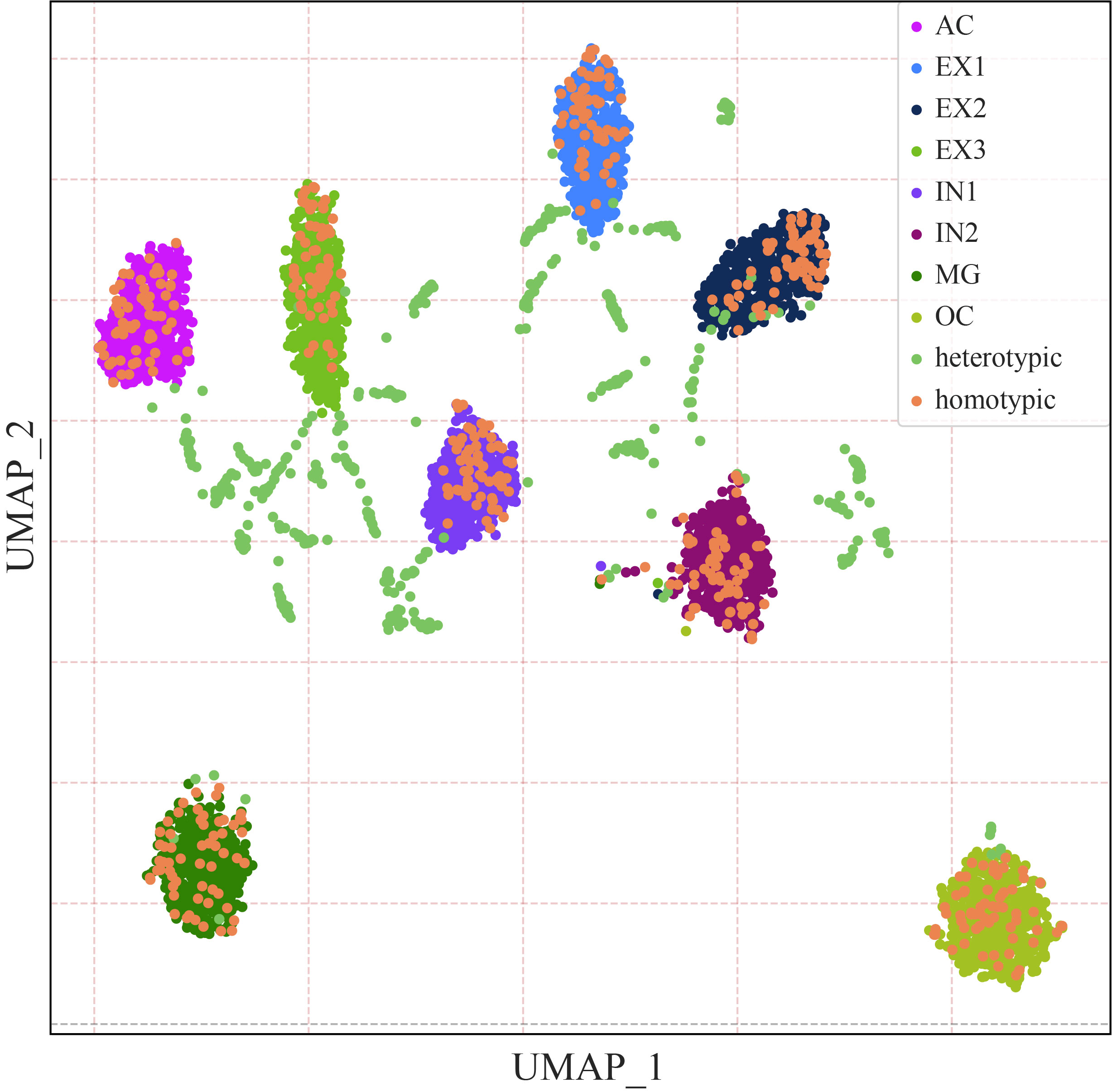


**Fig. S1** The impact of homotypic and heterotypic doublets. We used the simulated data to illustrate the impact of homotypic and heterotypic doublets. We first used one replicate of forebrain from MF that contains eight annotated cell types, to generate the count matrix. The count matrix of single cell type was then used to separately generate corresponding fully-synthetic data by simATAC. Here, we generated the data of different cell types with equal size. The homotypic doublets are generated by picking two simulated singlets from the same cell type and then mixing their profiles. The heterotypic doublets are generated by picking two simulated singlets from different cell types and then mixing their profiles. As is clearly shown, the heterotypic doublets may confound the downstream analysis more seriously than the homotypic doublets. Therefore, we stressed the heterotypic doublet detection in this work.


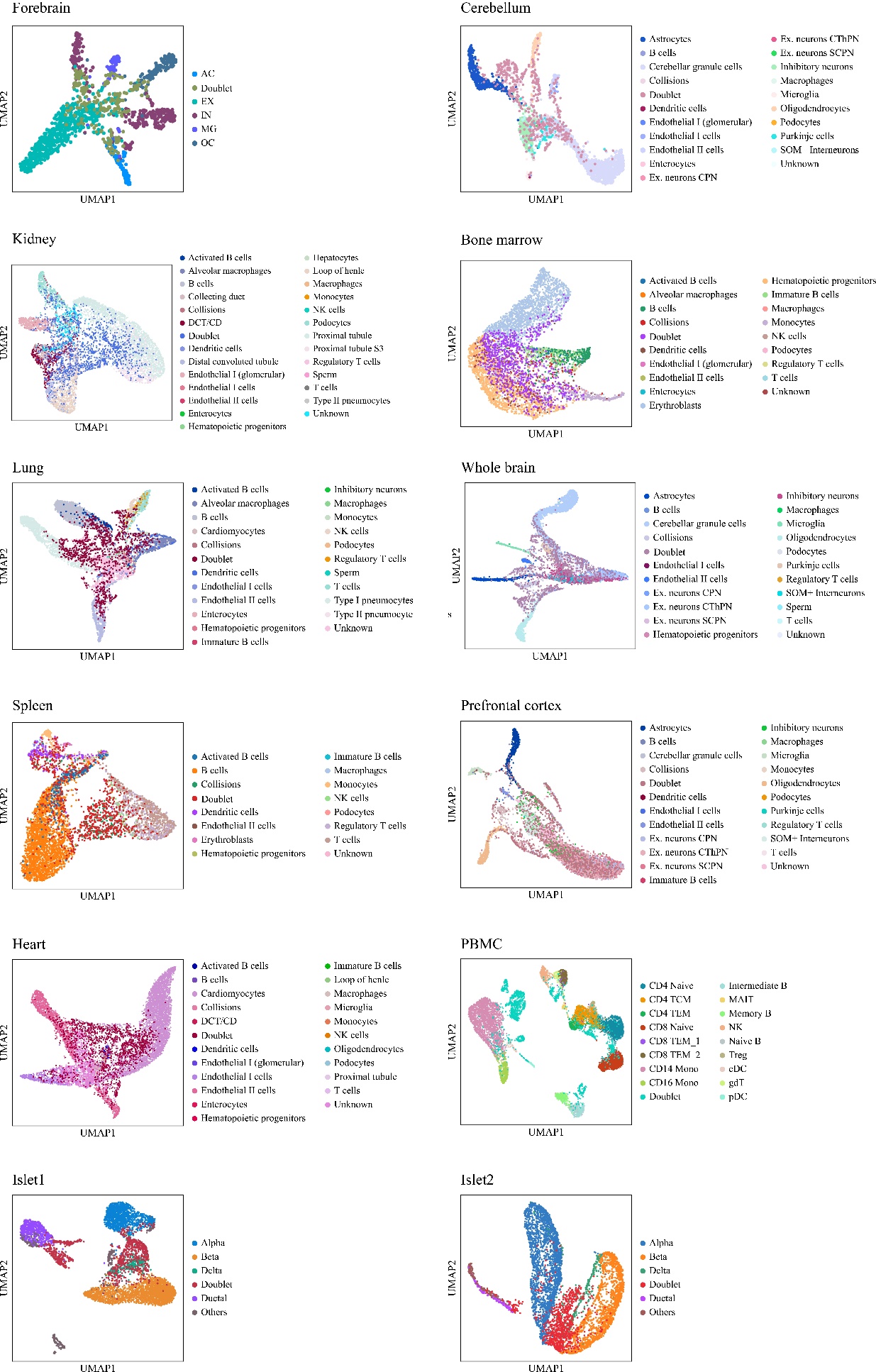
**Fig. S2** The UMAP of the semi-synthetic benchmark datasets to understand the complexity. The TF-IDF transformation was firstly performed on the count matrices, PCA and UMAP were subsequently performed on the transferred matrices using the default parameters in EpiScanpy.


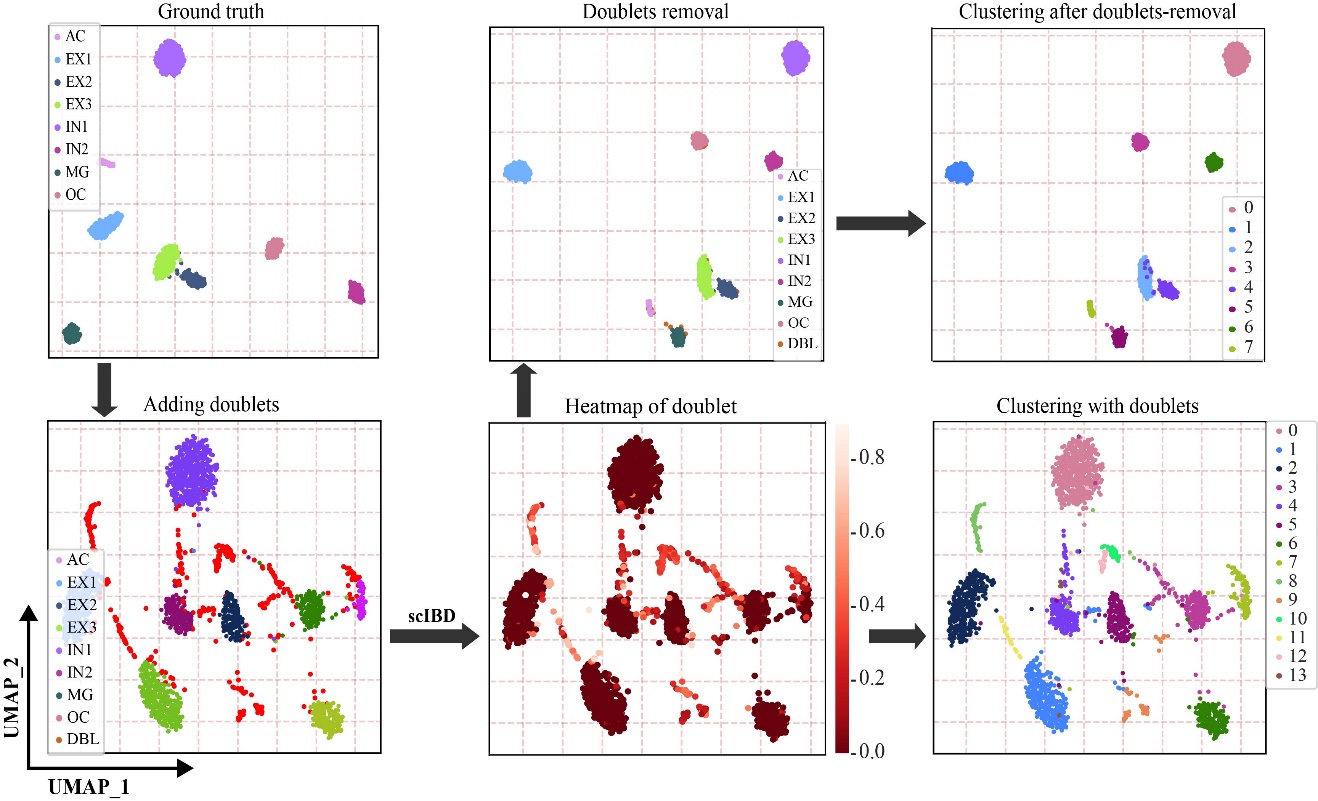
**Fig. S3** The illustration of the impact of doublets and the efficacy of doublet-removal by scIBD on the imbalanced fully-synthetic dataset where the sizes of different cell types are different. We first visualized the simulated singlets, which show great heterogeneity between different cell types as expected. After adding the simulated doublets, we can clearly observe that the doublets scatter between the cell types, resulting in spurious clustering results. The heatmap of doublet scores provided by scIBD is presented, where the droplets with high doublet scores are fairly consistent with the ground-truth doublets. To further illustrate the efficacy of scIBD in clustering, we removed the doublets called by scIBD. As clearly shown, the clusters reveal significant heterogeneity without the detected doublets, and the number of clusters obtained by default parameters is exactly equal to that of true cell types in the ground truth, showing the improvement of clustering by scIBD. Although some doublets failed to be detected, the remained doublets mostly lay at the edge of main cell types, and have negligible impact on the clustering results.


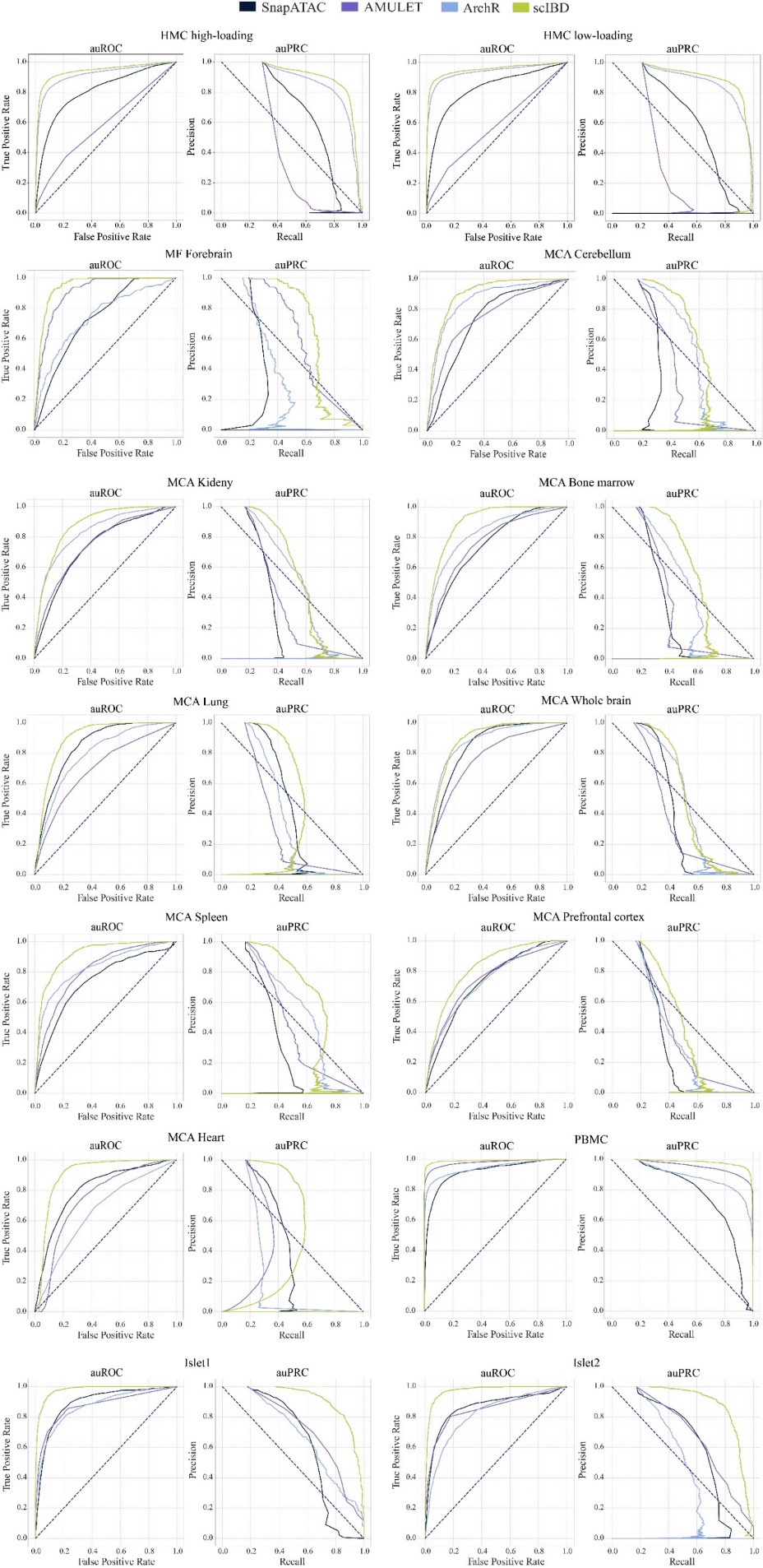
**Fig. S4** The receiver operating characteristic (ROC) curves and precision-recall (PR) curves of scIBD and the baseline methods on the benchmark datasets.


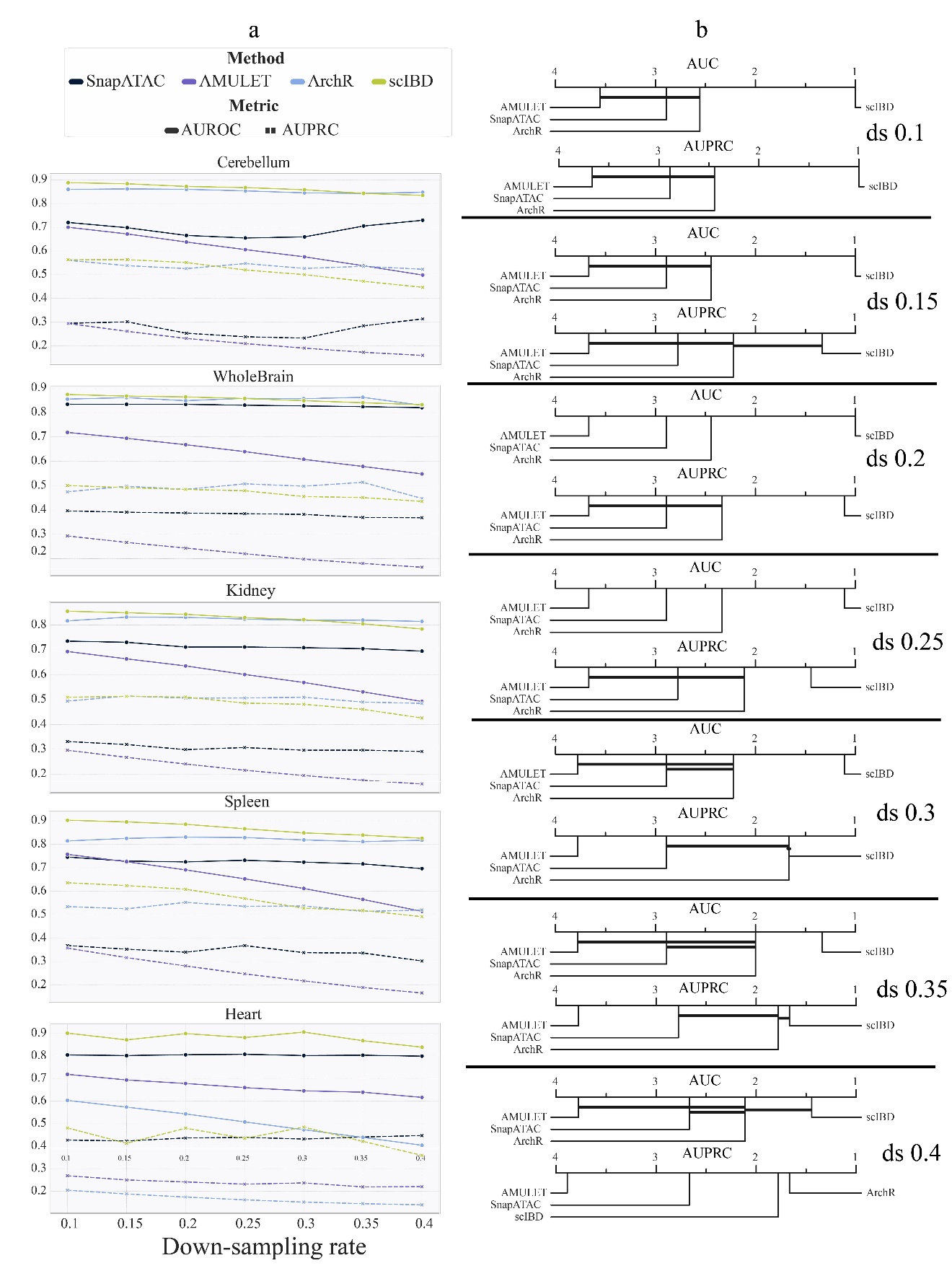
**Fig. S5** The performance evaluation on the semi-synthetic datasets where the doublets have different numbers of captured reads. **a** The AUROC (solid lines) and the AUPRC (dotted lines) show the trend of performance with the reads decrease of doublets on the rest five semi-synthetic datasets. **b** The critical difference diagrams (Wilcoxon signed-rank test with Holm’s alpha (5% two-sided) correction) of AUROC and AUPRC under different reads down-sampling rates over all the nine semi-synthetic datasets. The results show that scIBD achieves the best overall performance across all the semi-synthetic datasets.


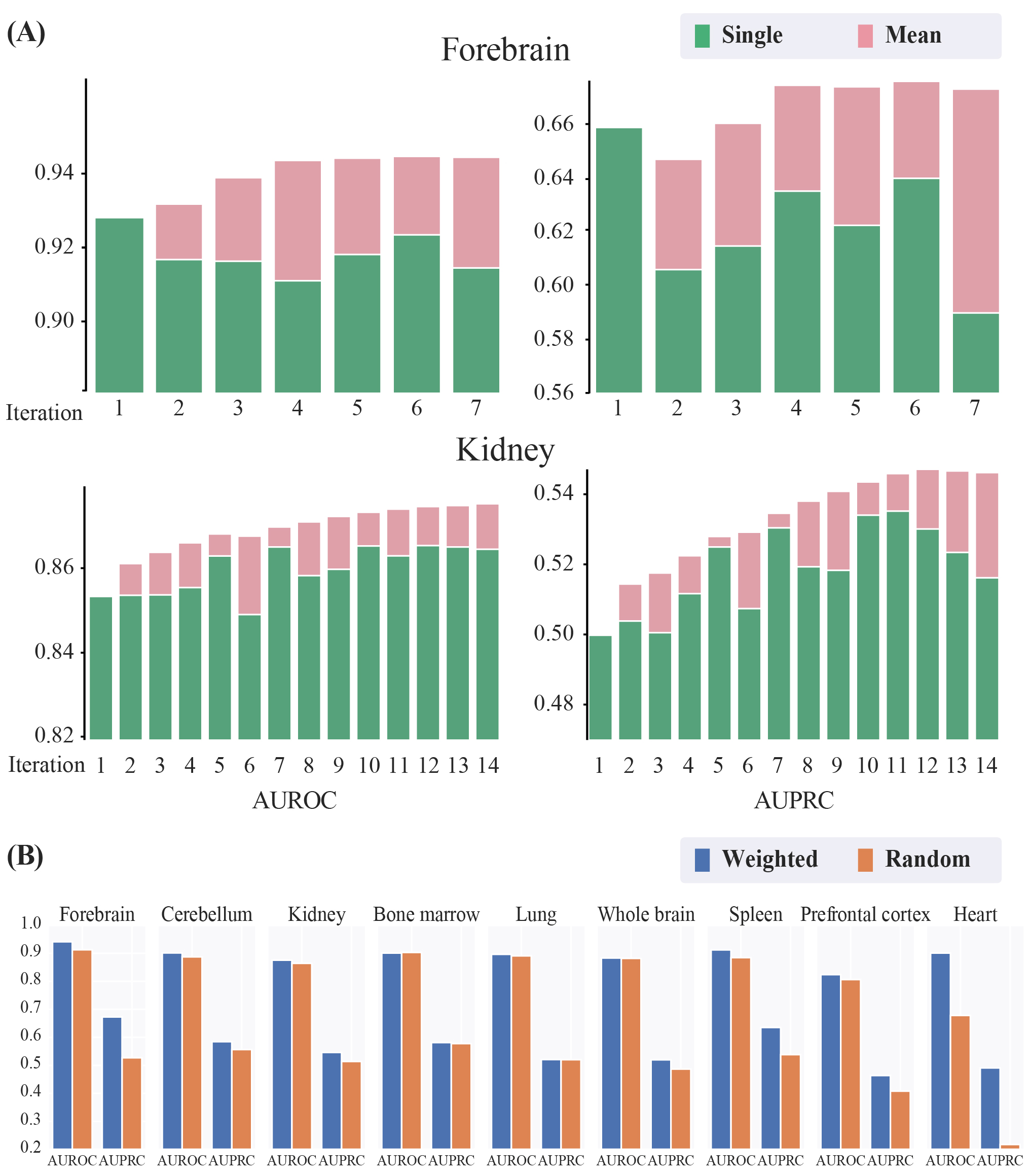
**Fig. S6** Ablation experiments. **a** The iterative-optimizing process. The AUROC and AUPRC of each iteration are illustrated in the plots, where the bottom bar represents the metrics calculated based on the doublet scores from the current iteration, while the top bar represents the metrics calculated based on the mean scores across all iterations former till the current one. **b** The performance boosted by our specific simulation strategy. The performance comparison between our proposed strategy and the traditional random strategy for simulating doublets.


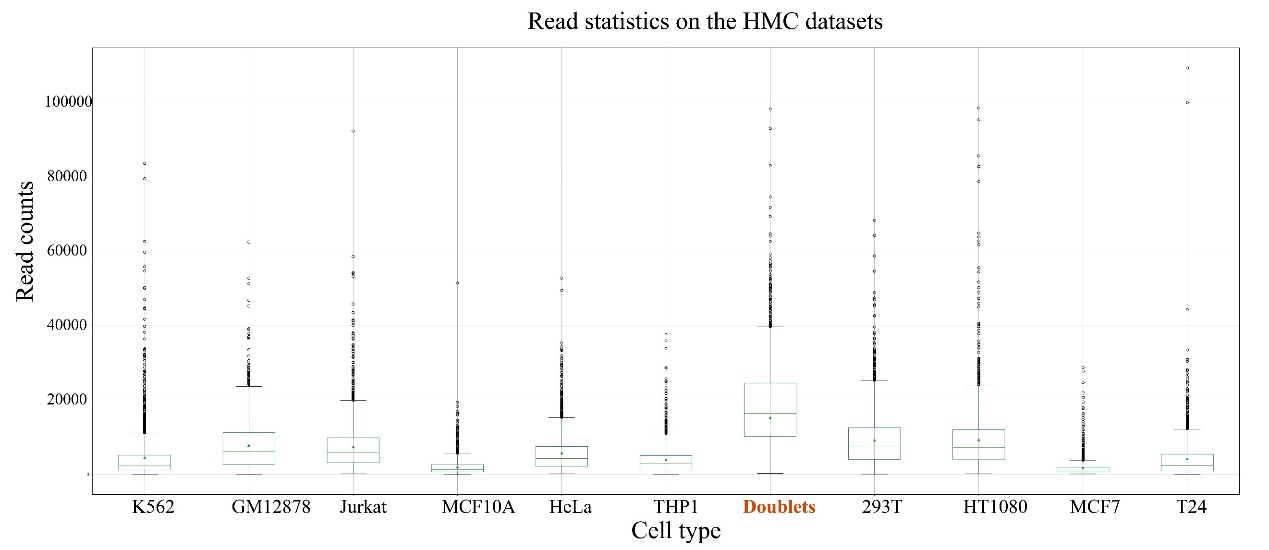
**Fig. S7** The sequencing depth statistics based on the HMC datasets. The sequencing depth of doublets is significantly higher than that of singlets.


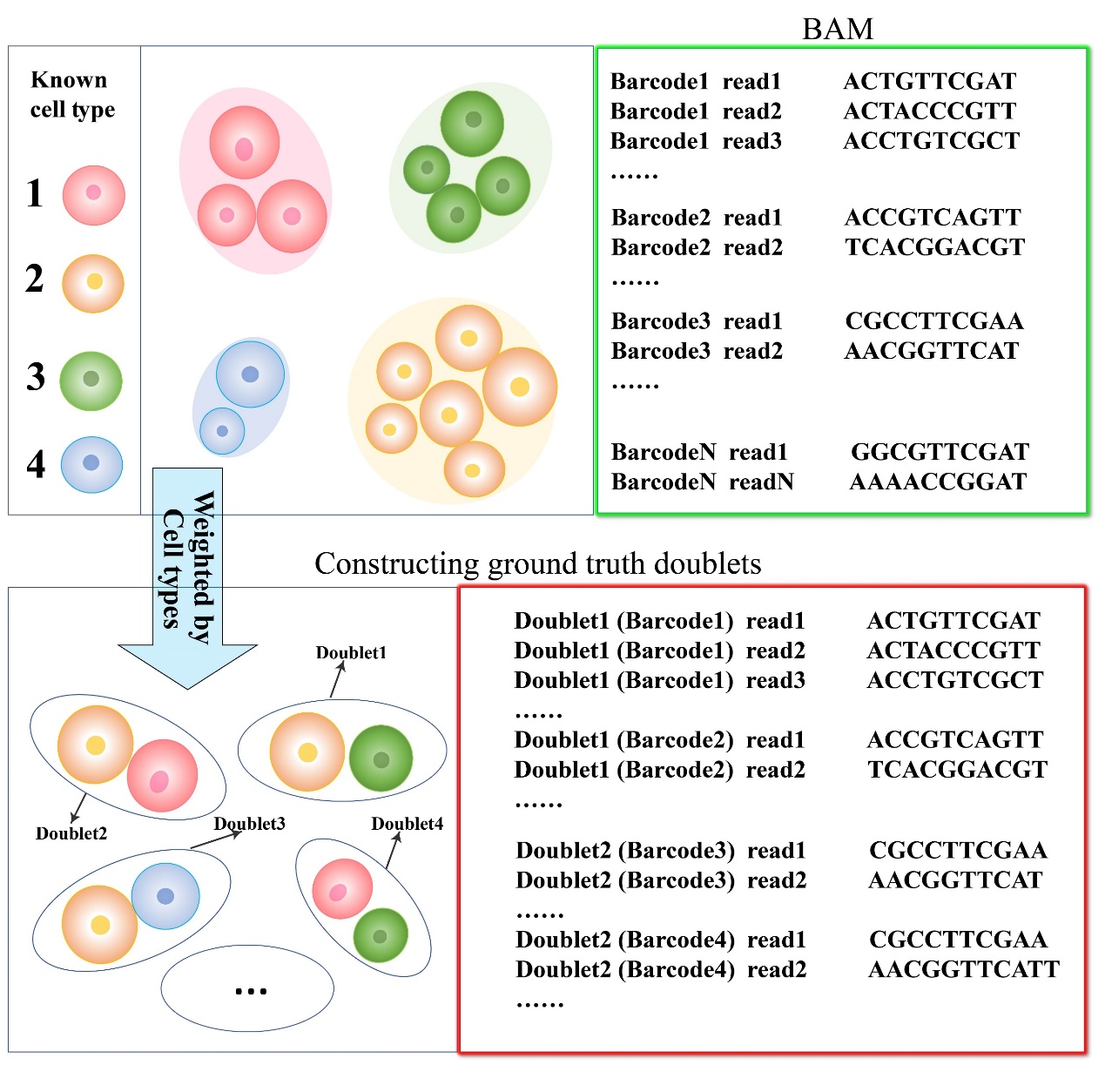
**Fig. S8** The pipeline of constructing ground-truth doublets in the semi-synthetic datasets. We mixed all the reads of two selected cells to construct a ground-truth doublet based on the BAM files. The raw BAM file of singlets and the generated BAM file of ground-truth doublets are concatenated as a complete BAM file for the subsequent process. The cells that are selected to construct benchmark doublets are weighted by the known cell annotations. Specifically, we obtained the proportions of all cell types based on their known annotations; according to the cell type proportions we then probabilistically selected two types, from which one cell was respectively randomly selected to form a heterotypic doublet. This simulation pipeline was repeatedly performed to simulate all ground-truth heterotypic doublets.


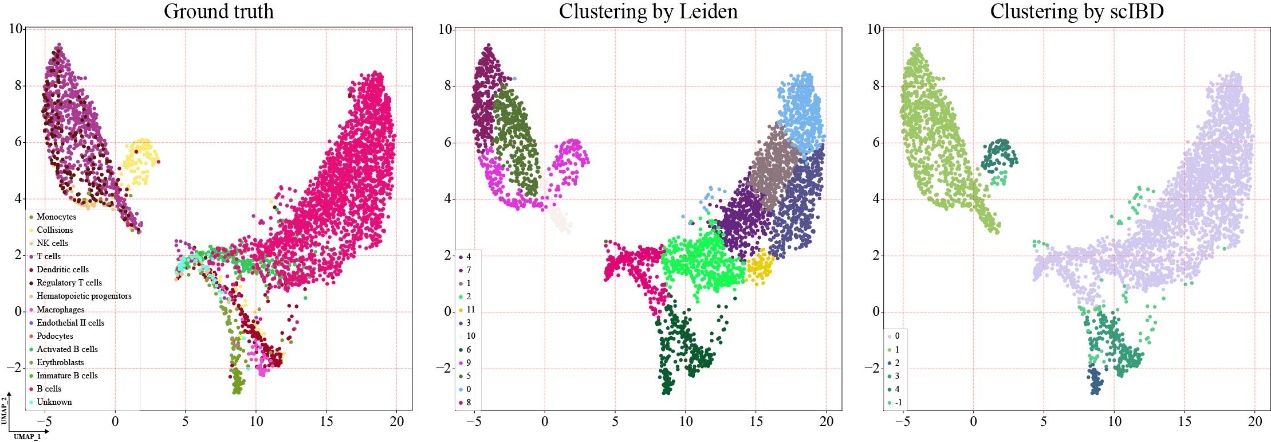
**Fig. S9** The difference of clustering between Leiden and scIBD. Using ground-truth annotations as the reference, Leiden tends to split the predominant cell class into several subclasses, thus yielding low-quality simulated doublets. scIBD applies a specific clustering pipeline that can reserve the main cell type, especially in an extremely-imbalanced dataset.
