# Supplementary material for: scIBD: a self-supervised iterative-optimizing model for boosting the detection of heterotypic doublets in single-cell chromatin accessibility data
Source: Genome Biol. 2023 Oct 9;24:225. doi: 10.1186/s13059-023-03072-y (PMC10561408; doi:10.1186/s13059-023-03072-y)
Supplement: Supplementary file 2 — Additional file 2: Table S1. The detailed performance metrics under the given doublet truncating rate of different methods based on their respective doublet scores.Table S2. Performance comparison on the Islets (Islet1 and Islet2) and PBMC datasets. Table S3. The number of overlapped differential accessible peaks (microglia vs. rest) between the doublets removed (RM) datasets by different methods and the ground truth (singlets). Table S4. Top 20 differential accessible peaks of the microglia cluster on the ground truth (GT), doublets retaining (RE), and doublets removal (RM) datasets. Table S5. The performance comparison between scIBD and the baseline methods that are designed for doublet-detection in scRNA-seq data. Table S6. The computational efficacy comparison of scIBD and the baseline methods. Table S7. Performance comparison on Islets datasets where the doublets are simulated with and without weighting criteria respectively.(DOCX 2433 KB) [file 13059_2023_3072_MOESM2_ESM.docx]

**scIBD: a self-supervised iterative-optimizing model for boosting the detection of heterotypic doublets in single-cell chromatin accessibility data**

Additional file 2: Supplementary tables

Wenhao Zhang, Rui Jiang, Shengquan Chen^*^ and Ying Wang*

**Table S1** **The detailed performance metrics under the given doublet truncating rate of different methods based on their respective doublet scores.**

| **Dataset** | **Calling rate** | **0.05** | | | **0.1** | | | **0.15** | | | **0.2** | | | **0.25** | | |
| --- | --- | --- | --- | --- | --- | --- | --- | --- | --- | --- | --- | --- | --- | --- | --- | --- |
|  |  | Pre | Rec | F1 | Pre | Rec | F1 | Pre | Rec | F1 | Pre | Rec | F1 | Pre | Rec | F1 |
| **Forebrain** | SnapATAC | 0.294 | 0.100 | 0.149 | 0.333 | 0.230 | 0.272 | 0.327 | 0.350 | 0.338 | 0.302 | 0.465 | 0.367 | 0.303 | 0.465 | 0.367 |
|  | AMULET | 0.648 | **0.295** | **0.405** | 0.616 | 0.425 | 0.503 | 0.569 | 0.575 | 0.572 | 0.522 | 0.710 | 0.602 | 0.488 | 0.790 | 0.603 |
|  | ArchR | 0.522 | 0.180 | 0.268 | 0.437 | 0.295 | 0.352 | 0.380 | 0.380 | 0.381 | 0.372 | 0.470 | 0.415 | 0.332 | 0.535 | 0.410 |
|  | scIBD | **0.672** | 0.225 | 0.337 | **0.679** | **0.455** | **0.545** | **0.660** | **0.640** | **0.650** | **0.622** | **0.830** | **0.711** | **0.561** | **0.905** | **0.712** |
| **Cerebellum** | SnapATAC | 0.242 | 0.068 | 0.106 | 0.288 | 0.143 | 0.191 | 0.333 | 0.259 | 0.292 | 0.333 | 0.380 | 0.355 | 0.333 | 0.390 | 0.358 |
|  | AMULET | 0.438 | 0.123 | 0.192 | 0.453 | 0.253 | 0.324 | 0.442 | 0.374 | 0.405 | 0.426 | 0.510 | 0.464 | 0.400 | 0.593 | 0.478 |
|  | ArchR | 0.618 | 0.185 | 0.285 | 0.601 | 0.355 | 0.447 | 0.588 | 0.525 | 0.555 | 0.547 | 0.651 | 0.595 | 0.499 | 0.735 | 0.594 |
|  | scIBD | **0.662** | **0.198** | **0.305** | **0.663** | **0.398** | **0.497** | **0.604** | **0.543** | **0.572** | **0.564** | **0.677** | **0.615** | **0.517** | **0.776** | **0.620** |
| **Kidney** | SnapATAC | 0.417 | 0.090 | 0.148 | 0.392 | 0.216 | 0.279 | 0.377 | 0.292 | 0.329 | 0.372 | 0.376 | 0.374 | 0.355 | 0.454 | 0.398 |
|  | AMULET | 0.518 | 0.148 | 0.230 | 0.459 | 0.260 | 0.332 | 0.423 | 0.367 | 0.393 | 0.391 | 0.433 | 0.411 | 0.376 | 0.484 | 0.424 |
|  | ArchR | **0.656** | 0.190 | **0.295** | 0.622 | 0.371 | 0.465 | 0.576 | 0.508 | 0.545 | 0.499 | 0.599 | 0.545 | 0.450 | 0.671 | 0.538 |
|  | scIBD | 0.636 | **0.191** | 0.293 | **0.625** | **0.375** | **0.469** | **0.567** | **0.510** | **0.547** | **0.514** | **0.617** | **0.561** | **0.476** | **0.714** | **0.571** |
| **Bone**  **marrow** | SnapATAC | 0.478 | 0.088 | 0.149 | 0.406 | 0.206 | 0.273 | 0.388 | 0.302 | 0.340 | 0.370 | 0.404 | 0.386 | 0.347 | 0.500 | 0.409 |
|  | AMULET | 0.409 | 0.119 | 0.184 | 0.418 | 0.237 | 0.302 | 0.423 | 0.379 | 0.400 | 0.391 | 0.430 | 0.410 | 0.378 | 0.532 | 0.442 |
|  | ArchR | 0.612 | 0.182 | 0.281 | 0.596 | 0.363 | 0.451 | 0.545 | 0.490 | 0.516 | 0.484 | 0.590 | 0.532 | 0.434 | 0.657 | 0.522 |
|  | scIBD | **0.668** | **0.200** | **0.308** | **0.634** | **0.380** | **0.475** | **0.606** | **0.545** | **0.573** | **0.550** | **0.659** | **0.599** | **0.505** | **0.756** | **0.605** |
| **Lung** | SnapATAC | 0.555 | 0.118 | 0.195 | 0.525 | 0.312 | 0.391 | 0.508 | 0.382 | 0.436 | 0.472 | 0.514 | 0.492 | 0.431 | 0.622 | 0.509 |
|  | AMULET | 0.433 | 0.130 | 0.200 | 0.403 | 0.229 | 0.292 | 0.365 | 0.326 | 0.345 | 0.349 | 0.387 | 0.367 | 0.334 | 0.466 | 0.389 |
|  | ArchR | 0.526 | 0.158 | 0.243 | 0.464 | 0.277 | 0.347 | 0.434 | 0.391 | 0.411 | 0.409 | 0.489 | 0.445 | 0.397 | 0.585 | 0.473 |
|  | scIBD | **0.528** | **0.158** | **0.243** | **0.580** | **0.348** | **0.435** | **0.581** | **0.522** | **0.550** | **0.548** | **0.658** | **0.598** | **0.509** | **0.764** | **0.611** |
| **Whole**  **brain** | SnapATAC | 0.504 | 0.118 | 0.191 | 0.452 | 0.243 | 0.316 | 0.436 | 0.414 | 0.425 | 0.425 | 0.498 | 0.458 | 0.396 | 0.625 | 0.485 |
|  | AMULET | 0.489 | 0.158 | 0.239 | 0.433 | 0.271 | 0.333 | 0.387 | 0.374 | 0.380 | 0.367 | 0.455 | 0.406 | 0.347 | 0.548 | 0.425 |
|  | ArchR | **0.608** | **0.212** | **0.314** | **0.555** | **0.388** | **0.457** | 0.503 | 0.435 | 0.424 | 0.481 | **0.673** | 0.561 | 0.434 | **0.759** | 0.553 |
|  | scIBD | 0.586 | 0.190 | 0.287 | 0.542 | 0.352 | 0.427 | **0.508** | **0.494** | **0.501** | **0.486** | 0.671 | **0.562** | **0.463** | 0.751 | **0.573** |
| **Spleen** | SnapATAC | 0.497 | 0.106 | 0.174 | 0.472 | 0.185 | 0.266 | 0.422 | 0.277 | 0.335 | 0.394 | 0.374 | 0.384 | 0.371 | 0.486 | 0.421 |
|  | AMULET | 0.577 | 0.190 | 0.286 | 0.534 | 0.318 | 0.399 | 0.491 | 0.419 | 0.452 | 0.441 | 0.506 | 0.472 | 0.408 | 0.611 | 0.489 |
|  | ArchR | **0.711** | **0.212** | **0.326** | 0.680 | 0.408 | 0.511 | 0.612 | 0.544 | 0.576 | 0.525 | 0.630 | 0.573 | 0.459 | 0.679 | 0.548 |
|  | scIBD | 0.680 | 0.204 | 0.314 | **0.743** | **0.445** | **0.557** | **0.697** | **0.627** | **0.660** | **0.599** | **0.718** | **0.653** | **0.534** | **0.801** | **0.641** |
| **Prefrontal**  **cortex** | SnapATAC | 0.430 | 0.095 | 0.155 | 0.393 | 0.202 | 0.267 | 0.374 | 0.277 | 0.318 | 0.357 | 0.357 | 0.357 | 0.339 | 0.445 | 0.385 |
|  | AMULET | 0.565 | 0.167 | 0.258 | 0.478 | 0.277 | 0.351 | 0.432 | 0.379 | 0.404 | 0.397 | 0.451 | 0.422 | 0.380 | 0.496 | 0.430 |
|  | ArchR | 0.586 | 0.180 | 0.270 | 0.491 | 0.297 | 0.370 | 0.414 | 0.378 | 0.396 | 0.374 | 0.458 | 0.412 | 0.342 | 0.516 | 0.412 |
|  | scIBD | **0.588** | **0.180** | **0.270** | **0.532** | **0.319** | **0.399** | **0.498** | **0.448** | **0.472** | **0.463** | **0.555** | **0.505** | **0.424** | **0.636** | **0.508** |
| **Heart** | SnapATAC | 0.486 | 0.102 | 0.183 | 0.499 | 0.244 | 0.327 | 0.483 | 0.370 | 0.419 | 0.456 | 0.515 | 0.484 | 0.417 | 0.618 | 0.498 |
|  | AMULET | 0.048 | 0.014 | 0.022 | 0.194 | 0.114 | 0.144 | 0.324 | 0.291 | 0.306 | 0.369 | 0.435 | 0.400 | 0.364 | 0.535 | 0.433 |
|  | ArchR | 0.270 | 0.074 | 0.116 | 0.280 | 0.164 | 0.207 | 0.293 | 0.240 | 0.264 | 0.294 | 0.291 | 0.292 | 0.288 | 0.354 | 0.318 |
|  | scIBD | **0.347** | **0.104** | **0.160** | **0.527** | **0.316** | **0.395** | **0.587** | **0.528** | **0.556** | **0.584** | **0.701** | **0.637** | **0.548** | **0.821** | **0.657** |
| **Islet1** | SnapATAC | 0.749 | 0.168 | 0.275 | 0.699 | 0.367 | 0.481 | 0.675 | 0.482 | 0.562 | 0.611 | 0.682 | 0.644 | 0.566 | 0.735 | 0.639 |
|  | AMULET | 0.878 | 0.244 | 0.382 | 0.844 | 0.371 | 0.516 | 0.747 | 0.570 | 0.647 | 0.638 | 0.701 | 0.668 | 0.638 | 0.701 | 0.668 |
|  | ArchR | 0.893 | 0.255 | 0.397 | 0.739 | 0.422 | 0.537 | 0.665 | 0.571 | 0.614 | 0.590 | 0.668 | 0.626 | 0.513 | 0.735 | 0.604 |
|  | scIBD | **0.988** | **0.282** | **0.439** | **0.949** | **0.543** | **0.691** | **0.886** | **0.761** | **0.818** | **0.767** | **0.878** | **0.819** | **0.662** | **0.947** | **0.779** |
| **Islet2** | SnapATAC | 0.755 | 0.119 | 0.205 | 0.730 | 0.353 | 0.476 | 0.692 | 0.485 | 0.571 | 0.614 | 0.664 | 0.638 | 0.565 | 0.733 | 0.638 |
|  | AMULET | 0.906 | 0.217 | 0.350 | 0.814 | 0.356 | 0.495 | 0.737 | 0.448 | 0.557 | 0.661 | 0.606 | 0.633 | 0.661 | 0.606 | 0.633 |
|  | ArchR | 0.602 | 0.173 | 0.268 | 0.595 | 0.343 | 0.435 | 0.537 | 0.465 | 0.498 | 0.497 | 0.567 | 0.529 | 0.452 | 0.648 | 0.532 |
|  | scIBD | **0.946** | **0.274** | **0.425** | **0.907** | **0.526** | **0.666** | **0.855** | **0.744** | **0.795** | **0.767** | **0.889** | **0.823** | **0.650** | **0.944** | **0.770** |
| **PBMC** | SnapATAC | 0.916 | 0.230 | 0.368 | 0.865 | 0.473 | 0.611 | 0.745 | 0.670 | 0.705 | 0.660 | 0.765 | 0.709 | 0.581 | 0.839 | 0.686 |
|  | AMULET | 1.000 | 0.282 | 0.440 | 1.000 | 0.596 | 0.747 | 0.964 | 0.853 | 0.905 | **0.813** | 0.922 | 0.864 | **0.732** | 0.939 | **0.822** |
|  | ArchR | 1.000 | 0.300 | 0.461 | 0.985 | 0.590 | 0.738 | 0.881 | 0.792 | 0.834 | 0.717 | 0.854 | 0.779 | 0.598 | 0.881 | 0.713 |
|  | scIBD | **1.000** | **0.300** | **0.461** | **0.996** | **0.597** | **0.747** | **0.985** | **0.886** | **0.933** | 0.809 | **0.971** | **0.883** | 0.657 | **0.985** | 0.788 |

**Table S2** Performance comparison on the Islets (Islet1 and Islet2) and PBMC datasets.

| **Datasets** | **Metrics** | **SnapATAC** | **AMULET** | **ArchR** | **scIBD** |
| --- | --- | --- | --- | --- | --- |
| Islet1 | AUROC | 0.891 | 0.872 | 0.869 | **0.978** |
|  | AUPRC | 0.613 | 0.675 | 0.680 | **0.913** |
| Islet2 | AUROC | 0.862 | 0.848 | 0.816 | **0.973** |
|  | AUPRC | 0.609 | 0.624 | 0.486 | **0.882** |
| PBMC | AUROC | 0.918 | 0.979 | 0.943 | **0.991** |
|  | AUPRC | 0.755 | 0.950 | 0.889 | **0.979** |

**Table S3** The number of overlapped differential accessible peaks (microglia vs. rest) between the doublets removed (RM) datasets by different methods and the ground truth (singlets).

| **Top N differential peaks** | **scIBD** | **ArchR** | **AMULET** | **SnapATAC** |
| --- | --- | --- | --- | --- |
| 20 | **10** | 8 | 10 | 10 |
| 50 | **32** | 25 | 25 | 31 |
| 100 | **70** | 60 | 64 | 59 |
| 200 | **137** | 130 | 134 | 132 |
| 500 | **365** | 328 | 337 | 324 |
| 1000 | **751** | 672 | 715 | 671 |

*Note:* The bold style represents the best performance.

**Table S4** Top 20 differential accessible peaks of the microglia cluster on the ground truth (GT), doublets retaining (RE), and doublets removal (RM) datasets.

| **GT (singlets)** | **RE (singlets+doublets)** | **RM (doubelts removed by scIBD)** | **Relevant genes** | |
| --- | --- | --- | --- | --- |
| chr18_61107751_61108750 | **chr18_61107751_61108750** | **chr18_61107751_61108750** |  |  |
| chrUn_JH584304_9533_10532 | **chr9_22814719_22815718** | **chrUn_JH584304_9533_10532** |  |  |
| chr12_8521412_8522411 | **chrUn_JH584304_9533_10532** | **chr9_22814719_22815718** |  |  |
| chr9_22814719_22815718 | chr11_26639796_26640795 | chr4_141952051_141953050 |  |  |
| chr12_103343139_103344138 | chr3_131642514_131643513 | *chr7_89270169_89271168 | *Tmem135* |  |
| chr7_145111135_145112134 | chr3_105036143_105037142 | **chr15_88490221_88491220** |  |  |
| chr8_115555063_115556062 | chr14_40936526_40937525 | *******chr10_126502065_126503064** | None |  |
| chr3_95673590_95674589 | **chr7_67445175_67446174** | ***chr8_115555063_115556062** | None |  |
| chr18_15194451_15195450 | chr17_78917257_78918256 | *******chr3_95673590_95674589** | *Gm52627* (lncrna) |  |
| chr2_144247010_144248009 | chr16_33921642_33922641 | *******chr12_103343139_103344138** | *Asb2* |  |
| chr7_66178824_66179823 | chr7_80078449_80079448 | *chr13_89383903_89384902 | None |  |
| chr15_88490221_88491220 | **chr15_88490221_88491220** | **chr9_107983128_107984127** |  |  |
| chr13_12412121_12413120 | chr9_107983128_107984127 | **chrUn_JH584304_26086_27085** |  |  |
| chr3_20026719_20027718 | **chr3_20026719_20027718** | chr16_33921642_33922641 |  |  |
| chr7_67445175_67446174 | chr4_141952051_141953050 | chr11_26639796_26640795 |  |  |
| chrUn_JH584304_26086_27085 | **chr7_145111135_145112134** | *chr18_5944528_5945527 | None |  |
| chr6_146221614_146222613 | chr6_116679090_116680089 | *chr5_68735031_68736030 | None |  |
| chr5_16469558_16470557 | **chr18_15194451_15195450** | *******chr13_12412121_12413120** | *Heatr1* |  |
| chr10_126502065_126503064 | **chrUn_JH584304_26086_27085** | chr3_131642514_131643513 |  |  |
| chr10_54117371_54118370 | **chr12_8521412_8522411** | *chr10_99459510_99460509 | *Gm35035* (lncrna) |  |

*Note:* * denotes the regions that are detected in RM but failed to be detected in RE. The bold style represents that the region is overlapped with GT.

**Table S5** The performance comparison between scIBD and the baseline methods that are designed for doublet-detection in scRNA-seq data.

|  | **hm-12k** | | **nuc-MULTI** | |
| --- | --- | --- | --- | --- |
|  | AUROC | AUPRC | AUROC | AUPRC |
| doubletCells [1] | 0.905 | 0.382 | 0.560 | 0.107 |
| Scrublet [2] | 0.992 | 0.932 | 0.732 | 0.356 |
| cxds [3] | **1.000** | **0.998** | 0.764 | 0.367 |
| bcds [3] | 0.968 | 0.594 | 0.763 | 0.355 |
| hybrid [3] | 0.995 | 0.952 | 0.772 | 0.383 |
| Solo [4] | **1.000** | 0.995 | 0.751 | 0.294 |
| DoubletDetection [5] | 0.979 | 0.810 | 0.770 | 0.422 |
| DoubletFinder [6] | 0.999 | 0.994 | 0.775 | 0.441 |
| scIBD | 0.998 | 0.995 | **0.790** | **0.487** |

*Note:* The results of the scRNA-seq-specific methods are referred from the review [7], the bold style represents the best performance.

**Table S6** The computational efficacy comparison of scIBD and the baseline methods

| **Datasets** | **Efficacy** | **scIBD** | **AMULET** | **ArchR** | **SnapATAC** |
| --- | --- | --- | --- | --- | --- |
| Forebrain  1298×226,759 | Running time (m) | 2.00 | 11.40 | 54.00 | 0.40 |
|  | Peak memory (GB) | 0.95 | 3.70 | 2.18 | 1.86 |
| Cerebellum  2733×221,657 | Running time (m) | 3.10 | 4.70 | 13.00 | 0.60 |
|  | Peak memory (GB) | 1.44 | 0.65 | 1.76 | 3.98 |
| Kidney  7717×190,690 | Running time (m) | 42.50 | 21.00 | 39.10 | 1.40 |
|  | Peak memory (GB) | 5.68 | 0.79 | 3.01 | 9.24 |
| Bone marrow  5244×254,413 | Running time (m) | 36.60 | 17.10 | 22.80 | 1.20 |
|  | Peak memory (GB) | 3.56 | 0.68 | 1.92 | 8.44 |
| Lung  6146×171,002 | Running time (m) | 19.60 | 12.90 | 24.60 | 1.40 |
|  | Peak memory (GB) | 3.61 | 0.65 | 2.19 | 6.69 |
| Whole brain  6494×189,933 | Running time (m) | 50.00 | 26.80 | 34.10 | 1.20 |
|  | Peak memory (GB) | 4.72 | 0.84 | 2.48 | 8.03 |
| Spleen  4824×157,363 | Running time (m) | 16.60 | 12.40 | 22.70 | 1.10 |
|  | Peak memory (GB) | 2.87 | 0.71 | 2.81 | 5.02 |
| Heart  9180×178,104 | Running time (m) | 36.20 | 23.40 | 36.00 | 1.90 |
|  | Peak memory (GB) | 7.40 | 0.87 | 2.60 | 10.20 |

**Table S7** Performance comparison on Islets datasets where the doublets are simulated with and without weighting criteria respectively.

| Simulation strategy | Datasets | Metrics | SnapATAC | AMULET | ArchR | scIBD |
| --- | --- | --- | --- | --- | --- | --- |
| Without weighting criterion | Islet1 | AUROC | 0.727 | 0.866 | 0.776 | **0.916** |
|  |  | AUPRC | 0.489 | 0.675 | 0.533 | **0.702** |
|  | Islet2 | AUROC | 0.687 | 0.851 | 0.791 | **0.928** |
|  |  | AUPRC | 0.404 | 0.616 | 0.478 | **0.642** |
| With  weighting criterion | Islet1 | AUROC | 0.891 | 0.872 | 0.869 | **0.978** |
|  |  | AUPRC | 0.613 | 0.675 | 0.680 | **0.913** |
|  | Islet2 | AUROC | 0.862 | 0.848 | 0.816 | **0.973** |
|  |  | AUPRC | 0.609 | 0.624 | 0.486 | **0.882** |

**References**

1. Lun A, McCarthy D, Marioni J: **A step-by-step workflow for low-level analysis of single-cell RNA-seq data with Bioconductor [version 2; peer review: 3 approved, 2 approved with reservations].** *F1000Research* 2016, **5**.

2. Wolock SL, Lopez R, Klein AM: **Scrublet: Computational Identification of Cell Doublets in Single-Cell Transcriptomic Data.** *Cell Systems* 2019, **8:**281-291.

3. Bais AS, Kostka D: **scds: computational annotation of doublets in single-cell RNA sequencing data.** *Bioinformatics* 2020, **36:**1150-1158.

4. Bernstein NJ, Fong NL, Lam I, Roy MA, Hendrickson DG, Kelley DR: **Solo: Doublet Identification in Single-Cell RNA-Seq via Semi-Supervised Deep Learning.** *Cell Systems* 2020, **11:**95-101.

5. Gayoso A, Shor J: **GitHub: DoubletDetection.** *Zenodo* 2019.

6. McGinnis CS, Murrow LM, Gartner ZJ: **DoubletFinder: Doublet Detection in Single-Cell RNA Sequencing Data Using Artificial Nearest Neighbors.** *Cell Systems* 2019, **8:**329-337.

7. Xi NM, Li JJ: **Benchmarking Computational Doublet-Detection Methods for Single-Cell RNA Sequencing Data.** *Cell Systems* 2021, **12:**176-194.
