# Supplementary material for: scIBD: a self-supervised iterative-optimizing model for boosting the detection of heterotypic doublets in single-cell chromatin accessibility data
Source: Genome Biol. 2023 Oct 9;24:225. doi: 10.1186/s13059-023-03072-y (PMC10561408; doi:10.1186/s13059-023-03072-y)
Supplement: Supplementary file 3 — Additional file 3: Content S1. Ablation experiments. Content S2. Computational efficacy comparison.Content S3. Pseudocode. Content S4. Metrics for evaluation. [file 13059_2023_3072_MOESM3_ESM.docx]

**scIBD: a self-supervised iterative-optimizing model for boosting the detection of heterotypic doublets in single-cell chromatin accessibility data**

Additional file 3: Supplementary contents

Wenhao Zhang, Rui Jiang, Shengquan Chen^*^ and Ying Wang*

**Content S1 Ablation experiments**

**S1.1 Contributions of the iterative process**

According to our proposed pipeline, the detected doublets in the former iterations no longer participate in the clustering and doublet simulation, so our method can focus on the remained doublets that are harder to detect, which makes the scores of the detected doublets in single iteration perform not very well. Therefore, we use the mean doublet scores across all iterations as the final doublet scores. As shown in Additional file 1: Fig. S4a, the bottom bars are the AUROC and AUPRC values which are calculated based on the doublet scores obtained in the current single iteration, while the top bars are the AUROC and AUPRC values calculated based on the mean doublet scores across all former iterations. The metrics using single iteration fluctuate with the iteration proceeds, while the AUROC and AUPRC using mean doublet scores are steadily improved by the iterative process and are always higher than those of single iteration.

**S1.2 Contributions of the simulation strategy**

We proposed a strategy to simulate artificial heterotypic doublets of high confidence, which are expected to improve the accuracy in finding the real heterotypic doublets. In classic simulation-based methods, doublets are created by complete-randomly picking two cells and mixing their profiles. We replace our specific simulation with a totally-random simulation, to validate its efficacy. As is shown in Additional file 1: Fig. S4b, the simulation strategy can improve the performance on most datasets, especially in AUPRC.

**Content S2 Computational efficacy comparison**

scIBD is an iterative-optimizing method to progressively detect the doublets. In each iteration, scIBD needs to implement the re-clustering, re-simulating, and re-detecting, thus it may consume more time on some datasets where the doublets are hard to detect. We have diligently optimized the speed of our algorithm by utilizing sparse-matrix operation in several steps of scIBD, including the TF-IDF transformation when clustering, the Jaccard distance calculation when detecting, to reduce the time taken in each iteration in the revised version of scIBD tool.

The latest version of our algorithm has achieved remarkable improvements in both running time and memory usage, surpassing the previous version by a large margin. For instance, on the Forebrain dataset, the running time has been slashed from about 10 minutes to a mere 2 minutes, and the peak memory usage has been reduced from about 3.00GB to a stunning 0.95GB.

To more comprehensively compare the computational efficacy of scIBD and the baseline methods, we selected several datasets with different data sizes and recorded the complete running time and the peak memory usage on a Linux server equipped with a 24-cores Intel(R) Xeon(R) CPU E5-2620 v2 @ 2.10GHz. All methods were executed with their default settings except for the expected doublet calling rate (set as the ground-truth doublet rate).

As shown in Additional file 2: Table S6, regarding running time, SnapATAC (Scrublet) emerges as the fastest method, while the other three methods, scIBD, ArchR and AMULET, exhibit longer running time and demonstrate varying advantages on different datasets. Regarding memory usage, AMULET acts as the best method for that it only computes the statistical results of the sequencing reads based on BAM/fragment files, while the other three methods need the converted count matrices to detect the doublets. No matter in terms of running time or memory usage, scIBD generally achieves the second-best efficacy across the datasets.

Although SnapATAC (Scrublet) achieves the best efficacy performance on running time, it shows unsatisfactory memory usage, approximately double or triple that of scIBD. Moreover, it also achieves fluctuating performance across the benchmark datasets, which may be due to it was initially proposed for scRNA-seq data. Compared with AMULET which shows the best memory usage efficacy, scIBD achieves the comparable time taken, only lagging about one to two minutes on most datasets. AMULET also achieves a relatively good performance in detecting doublets on some datasets with high sequencing depth. However, for the nature that it detects doublets based on the statistical results of the reads, it shows sensitivity to the sequencing depth, yielding unstable doublet-detection performance on the lower-sequenced datasets.

In conclusion, although scIBD and the baseline methods have their unique advantages in terms of running time and memory usage, scIBD achieves the best-balanced performance on both computational efficacy and doublet detection efficacy. Users can select the most suitable method based on their specific requirements and constraints.

| **Pseudocode: The iterative process of doublet detection in scIBD** |
| --- |
| 1: **Initialization**: the KNN graphing strategy, the size of the raw droplet set ${\mathbf{X} = \{x}_{1},x_{2}, \ldots, x_{N}\}$ , the reference doublet score vector $\mathbf{L}\in\mathbb{R}^{N\times1}$ of the droplets in$\mathbf{X}$where the values are initially 0, the expected doublet ratio $p$, the simulating ratio of doublets $q$, the detected doublets set $\mathbf{D}\boldsymbol{=}\left\{ \boldsymbol{\emptyset} \right\}$. |
| 2: **while \|**$\mathbf{D\vert}< p * N$ **do** |
| 3: $\mathbf{X}_{sim}=\left\{ \boldsymbol{\emptyset} \right\}$ |
| 4: Perform clustering on the set where the droplets are unlabeled ${\mathbf{X}\boldsymbol{-D} = \{x}_{1},x_{2}, \ldots, x_{N-\vert\mathbf{D}\vert}\}$, get$C$clusters. |
| 5: **for** $( i =1:int( 0.7*q * (N-\vert\mathbf{D}\vert)))$ **do** |
| 6: Randomly pick two clusters $c_{1} , c_{2} C$ weighted by the cluster proportions. |
| 7: Randomly pick one droplet within the selected cluster, respectively, $x_{1} \mathbf{(X-D)}_{c_{1}}, x_{2} \mathbf{(X-D)}_{c_{2}}$. |
| 8: Take the union of count matrices of the droplets, ${sim}_{i}= x_{1} \vert x_{2}$. |
| $\mathbf{X}_{sim}= \mathbf{X}_{sim}\boldsymbol{+}{sim}_{i}$ |
| 9: **end for** |
| 10: **for** $( j =1:int( 0.3*q * (N-\vert\mathbf{D}\vert)))$ **do** |
| 11: Randomly pick two droplets among all droplets, $x_{1},x_{2} \mathbf{X-D}$. |
| 12: Take the count matrix union of the droplets, ${sim}_{j}= x_{1} \vert x_{2}$. |
| 13: $\mathbf{X}_{sim}= \mathbf{X}_{sim}\boldsymbol{+}{sim}_{j}$ |
| 14: **end for** |
| 15: Construct KNN graph and calculate doublet score $\mathbf{S}=\left( \mathbf{S}_{\mathbf{X-D}},\mathbf{S}_{\mathbf{D}},\mathbf{S}_{sim} \right)$. |
| 16: $s_{th}= {o(p(\mathbf{S}}_{\mathbf{X-D}}), p(\mathbf{S}_{sim}))+2std (\mathbf{S}_{sim})$ |
| 17: **if** $\max\left( \mathbf{S}_{\mathbf{X-D}} \right)<s_{th}$ **then** |
| 18: **do break** |
| 19:  **else**: |
| 20: $\mathbf{D}_{iter}=\left\{ x_{i} \right\}$and $\mathbf{S}_{iter}=\left\{ s_{i} \right\}\mathbf{if}s_{i}>s_{th}$. |
| 21: Scale $\mathbf{S}_{iter}$ between 0.1 and 0.9 as $\mathbf{S'}_{iter}$. |
| 22: Update the reference doublet score vector $\mathbf{L}$using$\mathbf{S'}_{iter}$. |
| 23: $\mathbf{D = D +D}_{iter}$ |
| 24: **end if** |
| 25: **end while** |
| 26: $\mathbf{S}_{final}=Mean\left( \mathbf{S}_{\mathbf{X-D}},\mathbf{S}_{\mathbf{D}} \right)$across all iterations. |

**Content S3 Pseudocode**

**Content S4 Metrics for evaluation**

We calculated the silhouette coefficient to quantify the heterogeneity of the datasets. Specifically, following the standard pipeline introduced in EpiScanpy [1] we performed TF-IDF on the count matrices, PCA and UMAP were subsequently performed on the transferred matrices using the default parameters. Finally, the obtained UMAP embeddings and the true cell type annotations $\mathbf{Y}$ are used to calculate the silhouette coefficient, which is to measure how well each data point is assigned to its cluster, based on the distance to other points in the same cluster and the nearest cluster. Here the cluster is the cell type annotation and the distance are calculated based on the embeddings. The silhouette coefficient ranges from -1 to 1, where a high value means that the dataset shows great compactness and heterogeneity, a low value means the dataset has less heterogeneity. The silhouette coefficient can be calculated as follows:

$silhouette coefficient (i)= \frac{b\left( i \right)-a(i)}{\max(a\left( i \right),b(i))}$ (1)

where $a\left( i \right)$ is the mean distance between $\mathrm{droplet}_{i}$ and all droplets in the same cell type and $b\left( i \right)$ is the mean distance between $\mathrm{droplet}_{i}$ and all droplets in the nearest cell type. The silhouette coefficient for the entire dataset is the mean of $silhouette coefficient (i)$ over all droplets.

In terms of evaluation metrics, we firstly evaluated the accuracy of results based on Area Under the Receiver Operating Characteristic (AUROC) and Area Under Precision-Recall Curve (AUPRC). Precision, Recall, F1 are also used to show more explicit comparisons in some evaluation experiments.

$Precision=\frac{\mathrm{TP}}{TP+FP}$ (2)

$Recall=\frac{\mathrm{TP}}{TP+FN}$ (3)

$F1=\frac{2*precision*recall}{precision+recall}$ (4)

In addition, considering that doublets can confound the downstream analyses, especially the clustering, we further performed Louvain on the doublet-removed datasets to evaluate scIBD and the baseline methods. The clustering results were evaluated based on Adjusted Rand Index (ARI) and Adjusted Mutual Information (AMI). The detailed descriptions of the evaluation metrics are given as follows:

Let *N* denotes the total number of cells, *T* denotes the true labels of cells, *P* denotes the clustering assignments. Rand index (RI) represents the probability that the predicted clusters and the real cell type labels will agree on a randomly chosen pair of cells.

$\mathrm{RI}=\frac{A+B}{\frac{1}{2}*N*\left( N-1 \right)}$, (5)

where *A* denotes the number of cells whose cell labels are totally consistent in *T* and *P*, and *B* denotes the number of cells whose cell labels are inconsistent in *T* and *P*, $\frac{1}{2}*N*\left( N-1 \right)$ is the total number of probable pairs between true labels and cluster labels.

ARI is an adjusted version of RI, where it adjusts for the expected agreement by chance, as follows:

$ARI=\frac{RI-E\left( \mathrm{RI} \right)}{\mathrm{MAX}\left( \mathrm{RI} \right)-E\left( \mathrm{RI} \right)}$, (6)

where $E\left( * \right)$ is the expectation.

AMI is based on mutual information (MI), which assesses the similarity between the cluster labels and the true cell type labels. Similar to ARI, AMI adjusts MI by considering the expected value under random clustering, which is calculated as follows:

$\mathrm{AMI}=\frac{MI-E\left( \mathrm{MI} \right)}{\mathrm{MEAN}\left( H\left( P \right),H\left( T \right) \right)-E\left( \mathrm{MI} \right)}$, (7)

where $H\left( * \right)$ denotes the entropy function, $E\left( * \right)$ is the expectations.

**References**

1. Danese A, Richter ML, Chaichoompu K, Fischer DS, Theis FJ, Colome-Tatche M: **EpiScanpy: integrated single-cell epigenomic analysis.** *Nature Communications* 2021, **12:**1-8.
