# Supplementary material for: scIBD: a self-supervised iterative-optimizing model for boosting the detection of heterotypic doublets in single-cell chromatin accessibility data
Source: Genome Biol. 2023 Oct 9;24:225. doi: 10.1186/s13059-023-03072-y (PMC10561408; doi:10.1186/s13059-023-03072-y)
Supplement: Supplementary file 4 — Additional file 4. Review history. [file 13059_2023_3072_MOESM4_ESM.docx]

Review History

**First round of review**

**Reviewer 1**

**Were you able to assess all statistics in the manuscript, including the appropriateness of statistical tests used?**

Yes: I did not find any inappropriateness in the statistical tests.

**Were you able to directly test the methods?**

No.

**Comments to author:**

In this work, the authors develop scIBD to detect doublets in single-cell chromatin accessibility (scCAS) data. While the problem of doublets has been better studied for scRNA-Seq data, limited previous work has been done that focuses on single-cell chromatin accessibility data. This could be a nice addition to the literature after polishing. scIBD is an iterative algorithm, where the doublet scores are updated in each iteration. One important component in scIBD is the step to generate simulated doublets, which guides the training process. The performance of scIBD is evaluated on synthetic data, semi-synthetic data and one real HMC dataset. The followings are my comments:

1. About the ground-truth of the real scCAS HMC data analysis. The result from Demuxlet is used as the ground-truth for evaluation of detecting doublets. Demuxlet is a method for doublet detection in scRNA-Seq data. More discussion should be provided for the rationale of using this as the ground-truth for evaluation.

2. The authors focus on AUROC and AUPRC as the criterion for evaluation. In practice, we usually choose a reasonable cutoff to call the doublets. It would be great if the authors can provide more metrics for evaluation. For example, x-axis as the total number of called doublets, y-axis the number of true doublets among them. This will give the audience more insight on how the methods are performing.

3. The simulation of high-confident artificial heterotypic doublets. This seems to me an important first step in starting the iterative algorithm of scIBD. The heterotypic doublets are simulated by aggregating droplets from two different cell clusters. The cluster labels are obtained from DBSCAN. How is the number of clusters chosen? Because for real datasets, we do not know the true number of cell types, how rigorous is scIBD when different numbers of cell clusters are specified?

4. On page 22, line 513. What is "left-truncated Gaussian" exactly? Truncated at where? Does the point that it is truncated affect the performance of the method?

5. It would be great to add the computation and memory usage of scIBD, and compare with other methods.

Minor

6. The authors call the iterative algorithm in scIBD as "iterative-boosting". However, it seems to me that the iterative procedure is in nature very different from the boosting algorithm that I know (e.g. Adaboost), although superficially the doublet scores (the weight for each observation) is updated in each iteration. In addition, boosting algorithms are all iterative, so "iterative-boosting" is a little bizarre.

**Reviewer 2**

**Were you able to assess all statistics in the manuscript, including the appropriateness of statistical tests used?**

No.

**Were you able to directly test the methods?**

No.

**Comments to author:**

Zhang et al introduce scIBD, which improves upon existing simulation-based doublet detection methods by generating artificial doublets from cells of distinct clusters and introducing iterative boosting to thoroughly identify doublets through multiple rounds of doublet detection. The authors show that scIBD outperforms existing methods, especially at low sequencing depths up to ~15k median reads per cell. Alhough the authors extensively evaluate their method and show favorable performance, the results remain unconvincing due to details of how doublets for evaluation are generated, read depth, and lack of details provided on the datasets being evaluated. In addition the novelty hinges on two ideas which could readily be adapted to existing simulation-based methods. Overall, there are critical issues that must be addressed in the performance evaluation to convincingly show that scIBD is a suitable improvement over existing methods.

Major Comments:

1) The authors should provide a clear view of each of the datasets being evaluated. Markers of cell types should be shown so that readers can understand the complexity of the dataset being evaluated for doublet detection and how similar cell types may be to one another. It is difficult to discern these details otherwise.

2) Supplementary table S1 shows AMULET performing better (0.405 F1) than scIBD (0.337 F1) for doublet detection rate 0.05. It is not the true that: "no matter which doublet rate was used for detection, scIBD outperformed the baseline methods on Precision, Recall, and F1." (lines 261-263).

3) How well does scIBD perform when different cell populations have similar epigenetic profiles? It would be helpful to test scIBD on such datasets, for example, the available datasets from AMULET (Thibodeau & Eroglu et al. 2021): human pancreatic islets and human peripheral blood mononuclear cells (PBMCs).

4) The authors should make it clear that the doublets detected by Demuxlet represent those that are between donors and that doublets within the same donor cannot be detected by Demuxlet.

5) In the caption of Figure S6, it is mentioned: "The cells that are selected to construct benchmark doublets are weighted by the known cell annotations, namely, the larger size the cell type holds, the more likely it may generate a heterotypic doublet". More details should be provided for this construction of heterotypic doublets in the semi-synthetic evaluations in methods. How similar is this doublet selection for evaluation to the artificial doublet creation criteria used by scIBD to detect doublets? It would be helpful to understand the performances when this weighting criteria is not used for generating doublets for evaluation.

6) In the current schema for semi-synthetic evaluations, artificial doublets are generated to provide a ground truth set of doublets. However, this only allows for a proper estimate of recall. The ground truth for singlets may still contain doublets. The authors should discuss the caveats of this approach when evaluating performances. Some additional analyses are needed to understand precision. For example, evaluating singlet cells to establish high confidence singlets.

7) Results are demonstrated for up to ~15k median reads per cell (Mouse Forebrain dataset). It would be helpful to demonstrate scIBD's performance for more deeply sequenced data to understand how well scIBD scales.

8) There is a bug when trying to use the tool when it tries to pass 'min_samples' to DBSCAN in the GetStrategy method. The min_samples passed ends up being a floating point number instead of an integer. "The 'min_samples' parameter of DBSCAN must be an int in the range [1, inf). Got 31.67 instead."

Minor:

1) Precision and recall should be reported for the fully synthetic data evaluation and other areas where these calculations are missing.

2) It would be helpful to understand the runtime performance of scIBD in comparison with other methods.

3) It would be helpful for the authors to briefly discuss the typical doublet rate observed in scCAS data (e.g., what's described in Lareau et al. 2020, reference 51) to help readers understand performances observed in Table S1.

**Authors Response**

**Point-by-point responses to the reviewers’ comments:**

Reviewer #1:

In this work, the authors develop scIBD to detect doublets in single-cell chromatin accessibility (scCAS) data. While the problem of doublets has been better studied for scRNA-Seq data, limited previous work has been done that focuses on single-cell chromatin accessibility data. This could be a nice addition to the literature after polishing. scIBD is an iterative algorithm, where the doublet scores are updated in each iteration. One important component in scIBD is the step to generate simulated doublets, which guides the training process. The performance of scIBD is evaluated on synthetic data, semi-synthetic data and one real HMC dataset.

*Response: We thank the referee for the comments and suggestions on our works. The suggestions and advice were constructive and significantly improved the quality of the paper. We have addressed all comments, as described in the point-to-point response below.*

The followings are my comments:

1. About the ground-truth of the real scCAS HMC data analysis. The result from Demuxlet is used as the ground-truth for evaluation of detecting doublets. Demuxlet is a method for doublet detection in scRNA-Seq data. More discussion should be provided for the rationale of using this as the ground-truth for evaluation.

*Response: Thank you for your valuable suggestion. We give the discussions in Lines 181 to 191 of the revised manuscript Line.*

*The human mixed cell lines (HMC) datasets were generated based on the mixture of ten different cancer cell lines [1], where the doublets were annotated by Demuxlet [2]. Demuxlet is a general doublet-detection method based on the genotype variations, which have been applied to both single-cell RNA seq (scRNA-seq) data [2] and single-cell chromatin accessibility (scCAS) data [1]. By identifying genotype variations, Demuxlet can detect those doublets formed from different donors/samples with distinct genotype variations. Therefore, in HMC datasets, the doublets detected by Demuxlet can be considered as the heterotypic doublets from different cell lines (donors). Similarly, we also aim to stress the detection of the heterotypic doublets in scCAS data, thus it is reliable to use the HMC datasets where the ground-truth doublets were annotated by Demuxlet, as one kind of the benchmark datasets.*

*To further benchmark the performance of scIBD, more evaluation experiments were performed in the following four sections.*

*(1) The evaluations on the semi-synthetic datasets where the ground-truth doublets are manually constructed, to benchmark the performance on the complex datasets in more realistic scenes (Section 2.4 of the revised manuscript);*

*(2) The doublets-reads-down-sampling experiments to benchmark the performance of scIBD when detecting doublets with different numbers of captured reads (Section 2.5 of the revised manuscript);*

*(3) The additionally-carried benchmarking experiments on golden-standard datasets where the doublets were firstly removed to produce high-confident singlets and the ground-truth doublets were subsequently simulated (Section 2.6 of the revised manuscript);*

*(4) The complete reads-down-sampling experiments to benchmark scIBD of handling data with different sequencing depths (Section 2.6 of the revised manuscript);*

*(5) The downstream biological analyses to demonstrate the realistic function of scIBD (Section 2.7 of the revised manuscript).*

*We hope that the referee can agree that the comprehensive benchmarking on the more extensive datasets has demonstrated the superiority of scIBD.*

2. The authors focus on AUROC and AUPRC as the criterion for evaluation. In practice, we usually choose a reasonable cutoff to call the doublets. It would be great if the authors can provide more metrics for evaluation. For example, x-axis as the total number of called doublets, y-axis the number of true doublets among them. This will give the audience more insight on how the methods are performing.

*Response: We appreciate the insightful suggestion. We add the analysis according to the comment. The added descriptions are present in Lines 237 to 253 of the revised manuscript, and the detailed metrics are shown in Table S1 and Figure S4 of the revised supplementary materials.*

*The cutoff parameter, i.e., the expected doublet calling rate, is set as 0.1 by default in scIBD. The users can also manually set this parameter on demand. Here, to provide a comprehensive description of the benchmarking performance of scIBD and the baseline methods, apart from the AUROC and AUPRC, we additionally incorporated the detailed metrics, including Precision, Recall and F1. Considering the doublet rate is unknown in reality, we enumerated the cutoff, i.e., the doublet calling rate, from 0.05, which is the approximate doublet rate reported by Lareau et al. [3] and also the minimum simulation rate in semi-synthetic datasets, to 0.25, which is the maximum simulation rate in semi-synthetic datasets, at an interval of 0.05, and calculated the metrics on the semi-synthetic datasets to demonstrate the performance of the methods in detail. The results are presented in Table S1 of the revised supplementary materials, the corresponding area under receiver operating characteristic curve (auROC) and area under precision-recall curve (auPRC) plots are illustrated in Figure S4 of the revised supplementary materials.*

**Table S1.** The detailed performance metrics under different doublet calling rates of different methods based on their respective doublet scores.

| **Dataset** | **Calling rate** | **0.05** | | | **0.1** | | | **0.15** | | | **0.2** | | | **0.25** | | |
| --- | --- | --- | --- | --- | --- | --- | --- | --- | --- | --- | --- | --- | --- | --- | --- | --- |
|  |  | Pre | Rec | F1 | Pre | Rec | F1 | Pre | Rec | F1 | Pre | Rec | F1 | Pre | Rec | F1 |
| **Forebrain** | SnapATAC | 0.294 | 0.100 | 0.149 | 0.333 | 0.230 | 0.272 | 0.327 | 0.350 | 0.338 | 0.302 | 0.465 | 0.367 | 0.303 | 0.465 | 0.367 |
|  | AMULET | 0.648 | **0.295** | **0.405** | 0.616 | 0.425 | 0.503 | 0.569 | 0.575 | 0.572 | 0.522 | 0.710 | 0.602 | 0.488 | 0.790 | 0.603 |
|  | ArchR | 0.522 | 0.180 | 0.268 | 0.437 | 0.295 | 0.352 | 0.380 | 0.380 | 0.381 | 0.372 | 0.470 | 0.415 | 0.332 | 0.535 | 0.410 |
|  | scIBD | **0.672** | 0.225 | 0.337 | **0.679** | **0.455** | **0.545** | **0.660** | **0.640** | **0.650** | **0.622** | **0.830** | **0.711** | **0.561** | **0.905** | **0.712** |
| **Cerebellum** | SnapATAC | 0.242 | 0.068 | 0.106 | 0.288 | 0.143 | 0.191 | 0.333 | 0.259 | 0.292 | 0.333 | 0.380 | 0.355 | 0.333 | 0.390 | 0.358 |
|  | AMULET | 0.438 | 0.123 | 0.192 | 0.453 | 0.253 | 0.324 | 0.442 | 0.374 | 0.405 | 0.426 | 0.510 | 0.464 | 0.400 | 0.593 | 0.478 |
|  | ArchR | 0.618 | 0.185 | 0.285 | 0.601 | 0.355 | 0.447 | 0.588 | 0.525 | 0.555 | 0.547 | 0.651 | 0.595 | 0.499 | 0.735 | 0.594 |
|  | scIBD | **0.662** | **0.198** | **0.305** | **0.663** | **0.398** | **0.497** | **0.604** | **0.543** | **0.572** | **0.564** | **0.677** | **0.615** | **0.517** | **0.776** | **0.620** |
| **Kidney** | SnapATAC | 0.417 | 0.090 | 0.148 | 0.392 | 0.216 | 0.279 | 0.377 | 0.292 | 0.329 | 0.372 | 0.376 | 0.374 | 0.355 | 0.454 | 0.398 |
|  | AMULET | 0.518 | 0.148 | 0.230 | 0.459 | 0.260 | 0.332 | 0.423 | 0.367 | 0.393 | 0.391 | 0.433 | 0.411 | 0.376 | 0.484 | 0.424 |
|  | ArchR | **0.656** | 0.190 | **0.295** | 0.622 | 0.371 | 0.465 | 0.576 | 0.508 | 0.545 | 0.499 | 0.599 | 0.545 | 0.450 | 0.671 | 0.538 |
|  | scIBD | 0.636 | **0.191** | 0.293 | **0.625** | **0.375** | **0.469** | **0.567** | **0.510** | **0.547** | **0.514** | **0.617** | **0.561** | **0.476** | **0.714** | **0.571** |
| **Bone**  **marrow** | SnapATAC | 0.478 | 0.088 | 0.149 | 0.406 | 0.206 | 0.273 | 0.388 | 0.302 | 0.340 | 0.370 | 0.404 | 0.386 | 0.347 | 0.500 | 0.409 |
|  | AMULET | 0.409 | 0.119 | 0.184 | 0.418 | 0.237 | 0.302 | 0.423 | 0.379 | 0.400 | 0.391 | 0.430 | 0.410 | 0.378 | 0.532 | 0.442 |
|  | ArchR | 0.612 | 0.182 | 0.281 | 0.596 | 0.363 | 0.451 | 0.545 | 0.490 | 0.516 | 0.484 | 0.590 | 0.532 | 0.434 | 0.657 | 0.522 |
|  | scIBD | **0.668** | **0.200** | **0.308** | **0.634** | **0.380** | **0.475** | **0.606** | **0.545** | **0.573** | **0.550** | **0.659** | **0.599** | **0.505** | **0.756** | **0.605** |
| **Lung** | SnapATAC | 0.555 | 0.118 | 0.195 | 0.525 | 0.312 | 0.391 | 0.508 | 0.382 | 0.436 | 0.472 | 0.514 | 0.492 | 0.431 | 0.622 | 0.509 |
|  | AMULET | 0.433 | 0.130 | 0.200 | 0.403 | 0.229 | 0.292 | 0.365 | 0.326 | 0.345 | 0.349 | 0.387 | 0.367 | 0.334 | 0.466 | 0.389 |
|  | ArchR | 0.526 | 0.158 | 0.243 | 0.464 | 0.277 | 0.347 | 0.434 | 0.391 | 0.411 | 0.409 | 0.489 | 0.445 | 0.397 | 0.585 | 0.473 |
|  | scIBD | **0.528** | **0.158** | **0.243** | **0.580** | **0.348** | **0.435** | **0.581** | **0.522** | **0.550** | **0.548** | **0.658** | **0.598** | **0.509** | **0.764** | **0.611** |
| **Whole**  **brain** | SnapATAC | 0.504 | 0.118 | 0.191 | 0.452 | 0.243 | 0.316 | 0.436 | 0.414 | 0.425 | 0.425 | 0.498 | 0.458 | 0.396 | 0.625 | 0.485 |
|  | AMULET | 0.489 | 0.158 | 0.239 | 0.433 | 0.271 | 0.333 | 0.387 | 0.374 | 0.380 | 0.367 | 0.455 | 0.406 | 0.347 | 0.548 | 0.425 |
|  | ArchR | **0.608** | **0.212** | **0.314** | **0.555** | **0.388** | **0.457** | 0.503 | 0.435 | 0.424 | 0.481 | **0.673** | 0.561 | 0.434 | **0.759** | 0.553 |
|  | scIBD | 0.586 | 0.190 | 0.287 | 0.542 | 0.352 | 0.427 | **0.508** | **0.494** | **0.501** | **0.486** | 0.671 | **0.562** | **0.463** | 0.751 | **0.573** |
| **Spleen** | SnapATAC | 0.497 | 0.106 | 0.174 | 0.472 | 0.185 | 0.266 | 0.422 | 0.277 | 0.335 | 0.394 | 0.374 | 0.384 | 0.371 | 0.486 | 0.421 |
|  | AMULET | 0.577 | 0.190 | 0.286 | 0.534 | 0.318 | 0.399 | 0.491 | 0.419 | 0.452 | 0.441 | 0.506 | 0.472 | 0.408 | 0.611 | 0.489 |
|  | ArchR | **0.711** | **0.212** | **0.326** | 0.680 | 0.408 | 0.511 | 0.612 | 0.544 | 0.576 | 0.525 | 0.630 | 0.573 | 0.459 | 0.679 | 0.548 |
|  | scIBD | 0.680 | 0.204 | 0.314 | **0.743** | **0.445** | **0.557** | **0.697** | **0.627** | **0.660** | **0.599** | **0.718** | **0.653** | **0.534** | **0.801** | **0.641** |
| **Prefrontal**  **cortex** | SnapATAC | 0.430 | 0.095 | 0.155 | 0.393 | 0.202 | 0.267 | 0.374 | 0.277 | 0.318 | 0.357 | 0.357 | 0.357 | 0.339 | 0.445 | 0.385 |
|  | AMULET | 0.565 | 0.167 | 0.258 | 0.478 | 0.277 | 0.351 | 0.432 | 0.379 | 0.404 | 0.397 | 0.451 | 0.422 | 0.380 | 0.496 | 0.430 |
|  | ArchR | 0.586 | 0.180 | 0.270 | 0.491 | 0.297 | 0.370 | 0.414 | 0.378 | 0.396 | 0.374 | 0.458 | 0.412 | 0.342 | 0.516 | 0.412 |
|  | scIBD | **0.588** | **0.180** | **0.270** | **0.532** | **0.319** | **0.399** | **0.498** | **0.448** | **0.472** | **0.463** | **0.555** | **0.505** | **0.424** | **0.636** | **0.508** |
| **Heart** | SnapATAC | 0.486 | 0.102 | 0.183 | 0.499 | 0.244 | 0.327 | 0.483 | 0.370 | 0.419 | 0.456 | 0.515 | 0.484 | 0.417 | 0.618 | 0.498 |
|  | AMULET | 0.048 | 0.014 | 0.022 | 0.194 | 0.114 | 0.144 | 0.324 | 0.291 | 0.306 | 0.369 | 0.435 | 0.400 | 0.364 | 0.535 | 0.433 |
|  | ArchR | 0.270 | 0.074 | 0.116 | 0.280 | 0.164 | 0.207 | 0.293 | 0.240 | 0.264 | 0.294 | 0.291 | 0.292 | 0.288 | 0.354 | 0.318 |
|  | scIBD | **0.347** | **0.104** | **0.160** | **0.527** | **0.316** | **0.395** | **0.587** | **0.528** | **0.556** | **0.584** | **0.701** | **0.637** | **0.548** | **0.821** | **0.657** |
| **Islet1** | SnapATAC | 0.749 | 0.168 | 0.275 | 0.699 | 0.367 | 0.481 | 0.675 | 0.482 | 0.562 | 0.611 | 0.682 | 0.644 | 0.566 | 0.735 | 0.639 |
|  | AMULET | 0.878 | 0.244 | 0.382 | 0.844 | 0.371 | 0.516 | 0.747 | 0.570 | 0.647 | 0.638 | 0.701 | 0.668 | 0.638 | 0.701 | 0.668 |
|  | ArchR | 0.893 | 0.255 | 0.397 | 0.739 | 0.422 | 0.537 | 0.665 | 0.571 | 0.614 | 0.590 | 0.668 | 0.626 | 0.513 | 0.735 | 0.604 |
|  | scIBD | **0.988** | **0.282** | **0.439** | **0.949** | **0.543** | **0.691** | **0.886** | **0.761** | **0.818** | **0.767** | **0.878** | **0.819** | **0.662** | **0.947** | **0.779** |
| **Islet2** | SnapATAC | 0.755 | 0.119 | 0.205 | 0.730 | 0.353 | 0.476 | 0.692 | 0.485 | 0.571 | 0.614 | 0.664 | 0.638 | 0.565 | 0.733 | 0.638 |
|  | AMULET | 0.906 | 0.217 | 0.350 | 0.814 | 0.356 | 0.495 | 0.737 | 0.448 | 0.557 | 0.661 | 0.606 | 0.633 | 0.661 | 0.606 | 0.633 |
|  | ArchR | 0.602 | 0.173 | 0.268 | 0.595 | 0.343 | 0.435 | 0.537 | 0.465 | 0.498 | 0.497 | 0.567 | 0.529 | 0.452 | 0.648 | 0.532 |
|  | scIBD | **0.946** | **0.274** | **0.425** | **0.907** | **0.526** | **0.666** | **0.855** | **0.744** | **0.795** | **0.767** | **0.889** | **0.823** | **0.650** | **0.944** | **0.770** |
| **PBMC** | SnapATAC | 0.916 | 0.230 | 0.368 | 0.865 | 0.473 | 0.611 | 0.745 | 0.670 | 0.705 | 0.660 | 0.765 | 0.709 | 0.581 | 0.839 | 0.686 |
|  | AMULET | 1.000 | 0.282 | 0.440 | 1.000 | 0.596 | 0.747 | 0.964 | 0.853 | 0.905 | **0.813** | 0.922 | 0.864 | **0.732** | 0.939 | **0.822** |
|  | ArchR | 1.000 | 0.300 | 0.461 | 0.985 | 0.590 | 0.738 | 0.881 | 0.792 | 0.834 | 0.717 | 0.854 | 0.779 | 0.598 | 0.881 | 0.713 |
|  | scIBD | **1.000** | **0.300** | **0.461** | **0.996** | **0.597** | **0.747** | **0.985** | **0.886** | **0.933** | 0.809 | **0.971** | **0.883** | 0.657 | **0.985** | 0.788 |

*As clearly shown in Table S1, scIBD outperforms the baseline methods in terms of Precision, Recall, and F1 at most doublet calling rates across different datasets. In particular, compared with the baseline methods, scIBD can achieve higher Recall with the increase of calling rate, showcasing its superiority in removing more doublets when the real doublet rate is unknown. SnapATAC (Scrublet) shows fluctuant performance across different datasets, which may be due to it is scRNA-seq data originated, while the rest three methods show relatively robust performance, among which scIBD achieves overall the best and robust performance. Of note, the expected doublet calling rate is set as 0.1 by default in the tool of scIBD. The results also demonstrate that scIBD can achieve the best performance in terms of all metrics under the default expected calling rate of 0.1.*

***Figure S4.*** *The area under receiver operating characteristic curve (auROC) and area under precision-recall curves (auPRC) of scIBD and the baseline methods on the benchmark datasets.*

3. The simulation of high-confident artificial heterotypic doublets. This seems to me an important first step in starting the iterative algorithm of scIBD. The heterotypic doublets are simulated by aggregating droplets from two different cell clusters. The cluster labels are obtained from DBSCAN. How is the number of clusters chosen? Because for real datasets, we do not know the true number of cell types, how rigorous is scIBD when different numbers of cell clusters are specified?

*Response: Thank you for your valuable comment. We added the related detailed descriptions of the methods in Lines 563 to 571 of the revised manuscript.*

*DBSCAN is a density-based clustering algorithm that operates the assumption that clusters are dense regions in space separated by regions of lower density. It assigns the data points that are “densely grouped” with the same cluster label. One of the advantages of DBSCAN is that it does not require a fixed number of clusters as an input parameter. Instead, it involves two hyper-parameters “eps” and “min_samples”, to control the clustering results. A larger value of “eps” means that more points will be considered as neighbors, which can yield larger and fewer clusters. A larger “min_samples” means that a point needs more neighbors to be a core point, yielding fewer and denser clusters.
 In our work, we aim to detect the heterotypic doublets formed from different cell types, thus we need to simulate more high-confident heterotypic doublets by implementing clustering to tell the heterogeneity. As shown in Figure S9 of the revised supplementary materials, to preserve the predominant cell type clusters in an imbalanced sample, we applied a trade-off clustering method that uses the DBSCAN based on the UMAP embedding in our specifically-designed pseudo-dropleting-simulation strategy. In this strategy, the parameter “eps” is set as 0.1 and the parameter “min_samples” is self-adaptively set as 0.5% of the number of input droplets. The parameter settings were consistently used in all benchmarking experiments, and the results have demonstrated the robustness and reliability of our pipeline and the settings.*

***Figure S9.*** *The difference of clustering between Leiden and scIBD. Using ground-truth annotations as the reference, Leiden tends to split the predominant cell class into several subclasses, thus yielding low-quality simulated doublets. scIBD applies a specific clustering pipeline that can preserve the predominant cell type, especially in an extremely-imbalanced dataset.*

*Here, we also show the effect of the parameter “eps” and “min_samples” on the final performance of scIBD on the additional benchmark Islet1dataset, as shown in Table R1 as follows. We used the grid search to discover the effect of the parameters, we fixed the min_samples as 0.5% of the number of input droplets, and enumerated the eps from 0.05 to 0.3 at an interval of 0.05, then we fixed the eps as 0.1 and enumerated the min_samples from 0.1% to 0.9% at an interval of 0.2% of the number of the input droplets. When the parameter “eps” exceeds 0.2, the performance of scIBD remains unchanged for only one cluster is detected. Similarly, when the “min_samples” is too large, only one cluster is detected. Finally, we chose the parameter combination of “eps” and “min_samples” as 0.1 and 0.5% of the number of input droplets.*

**Table R1.** The effect of the clustering parameters on the performance of scIBD.

| (eps, min_samples) | AUROC | AUPRC |
| --- | --- | --- |
| (0.05, 0.5%) | 0.95 | 0.79 |
| **(0.1, 0.5%)** | **0.98** | **0.91** |
| (0.15, 0.5%) | 0.94 | 0.82 |
| (0.2, 0.5%) | 0.93 | 0.75 |
| (0.25, 0.5%) | 0.93 | 0.75 |
| (0.3, 0.5%) | 0.93 | 0.75 |
| (0.1, 0.1%) | 0.97 | 0.88 |
| (0.1, 0.3%) | 0.97 | 0.85 |
| (0.1, 0.7%) | 0.96 | 0.90 |
| (0.1, 0.9%) | 0.96 | 0.88 |

4. On page 22, line 513. What is "left-truncated Gaussian" exactly? Truncated at where? Does the point that it is truncated affect the performance of the method?

*Response: Thank you for your insightful comment. We deeply apologize for making such ambiguous descriptions, which makes it hard to understand. We have changed “the left-truncated Gaussian” to “the right side of a standard Gaussian” and re-illustrated the descriptions in Lines 631 to 637 of the revised manuscript.*

*We employ Gaussian distribution to model the doublet scores of different parts of droplets during the iterations. In each iteration, the doublets scores for all unlabeled droplets, S_raw, are calculated based on the results in former iterations and the newly-simulated doublets. Note that S_raw is composed of scores of singlets and undetected doublets, and S_sim are the scores of newly-simulated doublets in each iteration. As shown in Figure 7b (the grey histograms are the distribution of S_raw) of the revised manuscript, among the raw droplets, most droplets are singlets with a doublet score of zero, and doublets only account for a small portion of all the droplets. Therefore, the doublet scores of the droplets can be fitted as the “right side of a standard Gaussian distribution”, i.e., the number of droplets declines with the increase of doublet scores and none droplet has negative scores. We use the right side of a standard Gaussian to model to describe the doublet score distribution of unlabeled droplets, and a Gaussian to model . We have clarified the method in Lines 626 to 630 of the revised manuscript in this revision.*

***Figure 8b.*** *The distribution plot of the doublet scores during the iteration process. We separately show the doublet score distributions of three parts, the detected doublets in former iterations, the simulated doublets in each iteration and the unlabeled droplets in raw sets. In each iteration, we aim at separating doublets from the unlabeled droplets. The doublet scores of the unlabeled droplets are modeled by the right side of a standard Gaussian for most droplets are singlets with zero scores (grey). The scores of the simulated doublets (yellow) are used as the reference to obtain the threshold to determine the doublets in the unlabeled droplets.*

5. It would be great to add the computation and memory usage of scIBD, and compare with other methods.

Response: We appreciate the referee for this constructive suggestion. We have implemented the experiments and presented the results and discussion in Lines 443 to 445 of the revised manuscript, Content S2 and Table S6 of the revised supplementary materials.

scIBD is an iterative-optimizing method to progressively detect the doublets. In each iteration, scIBD needs to implement the re-clustering, re-simulating and re-detecting, thus it may consume more time on some datasets where the doublets are hard to detect. We have diligently optimized the speed of our algorithm by utilizing sparse-matrix operation in several steps of scIBD, including the TF-IDF transformation when clustering, the Jaccard distance calculation when detecting, to reduce the time taken in each iteration in the revised version of scIBD tool.

The latest version of our algorithm has achieved remarkable improvements in both running time and memory usage, surpassing the previous version by a large margin. For instance, on the Forebrain dataset, the running time has been slashed from about 10 minutes to a mere 2 minutes, and the peak memory usage has been reduced from about 3.00GB to a stunning 0.95GB.

To more comprehensively compare the computational efficacy of scIBD and the baseline methods, we selected several datasets with different data sizes and recorded the complete running time and the peak memory usage on a Linux server equipped with a 24-cores Intel(R) Xeon(R) CPU E5-2620 v2 @ 2.10GHz. All methods were executed with their default settings except for the expected doublet calling rate (set as the ground-truth doublet rate).

As shown in Table S6 of the revised supplementary, regarding running time, SnapATAC (Scrublet) emerges as the fastest method, while the other three methods, scIBD, ArchR and AMULET, exhibit longer running time and demonstrate varying advantages on different datasets. Regarding memory usage, AMULET acts as the best method for that it only computes the statistical results of the sequencing reads based on BAM/fragment files, while the other three methods need the converted count matrices to detect the doublets. No matter in terms of running time or memory usage, scIBD basically achieves the second-best efficacy across the datasets.

**Table S6.** The computational efficacy comparison of scIBD and the baseline methods.

| **Datasets** | **Efficacy** | **scIBD** | **AMULET** | **ArchR** | **SnapATAC** |
| --- | --- | --- | --- | --- | --- |
| Forebrain  1298´226,759 | Running time (m) | 2.00 | 11.40 | 54.00 | 0.40 |
|  | Peak memory (GB) | 0.95 | 3.70 | 2.18 | 1.86 |
| Cerebellum  2733´221,657 | Running time (m) | 3.10 | 4.70 | 13.00 | 0.60 |
|  | Peak memory (GB) | 1.44 | 0.65 | 1.76 | 3.98 |
| Kidney  7717´190,690 | Running time (m) | 42.50 | 21.00 | 39.10 | 1.40 |
|  | Peak memory (GB) | 5.68 | 0.79 | 3.01 | 9.24 |
| Bone marrow  5244´254,413 | Running time (m) | 36.60 | 17.10 | 22.80 | 1.20 |
|  | Peak memory (GB) | 3.56 | 0.68 | 1.92 | 8.44 |
| Lung  6146´171,002 | Running time (m) | 19.60 | 12.90 | 24.60 | 1.40 |
|  | Peak memory (GB) | 3.61 | 0.65 | 2.19 | 6.69 |
| Whole brain  6494´189,933 | Running time (m) | 50.00 | 26.80 | 34.10 | 1.20 |
|  | Peak memory (GB) | 4.72 | 0.84 | 2.48 | 8.03 |
| Spleen  4824´157,363 | Running time (m) | 16.60 | 12.40 | 22.70 | 1.10 |
|  | Peak memory (GB) | 2.87 | 0.71 | 2.81 | 5.02 |
| Heart  9180´178,104 | Running time (m) | 36.20 | 23.40 | 36.00 | 1.90 |
|  | Peak memory (GB) | 7.40 | 0.87 | 2.60 | 10.20 |

*Although SnapATAC (Scrublet) achieves the best efficacy performance on running time, it shows unsatisfactory memory usage, approximately double or triple that of scIBD. Moreover, it also achieves fluctuating performance across the benchmark datasets, which may be due to it was initially proposed for scRNA-seq data. Compared with AMULET which shows the best memory usage efficacy, scIBD achieves the comparable time taken, only lagging about one to two minutes on most datasets.*

*AMULET also achieves a relatively good performance in detecting doublets on some datasets with high sequencing depth. However, for the nature that it detects doublets based on the statistical results of the reads, it shows sensitivity to the sequencing depth, yielding unstable doublet-detection performance on the lower-sequenced datasets.*

*In conclusion, although scIBD and the baseline methods have their unique advantages in terms of running time and memory usage, scIBD achieves the best-balanced performance on both computational efficacy and doublet detection efficacy. Users can select the most suitable method based on their specific requirements and constraints.*

Minor

6. The authors call the iterative algorithm in scIBD as "iterative-boosting". However, it seems to me that the iterative procedure is in nature very different from the boosting algorithm that I know (e.g. Adaboost), although superficially the doublet scores (the weight for each observation) is updated in each iteration. In addition, boosting algorithms are all iterative, so "iterative-boosting" is a little bizarre.

*Response: Thank you for providing the thoughtful comment. We agree that the description “iterative-boosting” of scIBD can easily cause ambiguity with the traditional boosting algorithms. To avoid ambiguity, we deeply reflect on the process of our method and re-conceptualize our title as “iterative-optimizing”, the manuscript is finally entitled “A self-supervised iterative-optimizing model for boosting the detection of heterotypic doublets in single-cell chromatin accessibility data” in this revision.*

*We herein used the ‘iterative-boosting’ to stress that scIBD is an algorithm that can progressively learn the features of doublets and then find the more accurate doublets during the iterations. In each iteration, the doublets with high-confidence are detected, and the remaining droplets can produce more accurate clustering results and thereby simulate more high-confident doublets without the noisy doublets in the next iteration. Through the iterations, the potential doublets can be progressively detected with high precision. The procedure is in nature very different from the traditional algorithms.*

*To avoid ambiguity, we deeply reflect on the process of our method and re-conceptualize our title as “iterative-optimizing”. Iterative optimization is a process of refining and improving a solution through iterative steps. It involves continuously revising and optimizing a given solution by making small adjustments or changes in each iteration. This iterative approach allows for gradual improvement and refinement of the solution until an optimal or near-optimal solution is reached, which is in nature similar to the iterative process in scIBD. Therefore, we think “iterative-optimizing” can describe the essential process of scIBD. The manuscript is finally entitled “A self-supervised iterative-optimizing model for boosting the detection of heterotypic doublets in single-cell chromatin accessibility data”, which effectively conveys the key aspects of the scIBD algorithm, highlighting its iterative and optimizing nature, while also emphasizing its application in detecting heterotypic doublets in single-cell chromatin accessibility data.*

*We appreciate the referee again for the valuable comments and suggestions. We hope the additional experiments and the results in the revised manuscript can more comprehensively demonstrate the superiority of scIBD.*

**Reviewer 2**

**Were you able to assess all statistics in the manuscript, including the appropriateness of statistical tests used?**

No.

**Were you able to directly test the methods?**

No.

**Comments to author:**

Zhang et al introduce scIBD, which improves upon existing simulation-based doublet detection methods by generating artificial doublets from cells of distinct clusters and introducing iterative boosting to thoroughly identify doublets through multiple rounds of doublet detection. The authors show that scIBD outperforms existing methods, especially at low sequencing depths up to ~15k median reads per cell. Alhough the authors extensively evaluate their method and show favorable performance, the results remain unconvincing due to details of how doublets for evaluation are generated, read depth, and lack of details provided on the datasets being evaluated. In addition the novelty hinges on two ideas which could readily be adapted to existing simulation-based methods. Overall, there are critical issues that must be addressed in the performance evaluation to convincingly show that scIBD is a suitable improvement over existing methods.

*Response: We appreciate the referee for the constructive and valuable comments and suggestions, which have significantly improved our manuscript. We have addressed all comments, as described in the point-to-point response below.*

Major Comments:

1) The authors should provide a clear view of each of the datasets being evaluated. Markers of cell types should be shown so that readers can understand the complexity of the dataset being evaluated for doublet detection and how similar cell types may be to one another. It is difficult to discern these details otherwise.

*Response: We appreciate the referee for this insightful comment. We are sorry that the current descriptions of the benchmark datasets are not comprehensible enough, and we present the corresponding details and descriptions in Lines 127 to 133 of the revised manuscript, as well as in Table 1 and Figure S2 of the revised supplementary materials.*

*We added more descriptions for benchmark datasets, including the cell type number, the total valid read pairs, the average valid read pairs per droplet/nuclei and the median valid read pairs per droplet/nuclei, as shown in Table 1 of the revised manuscript. The fully-synthetic datasets were directly simulated in the count matrices of the Forebrain dataset, thus the descriptions of the read statistics in raw sequencing files are not available. Of note, all the droplets used in our benchmark datasets are those with cell type annotations that are publicly reported, which means that a portion of droplets with low quality has been removed from the raw sets. Therefore, the mean/median reads per droplet are normally higher than those reported originally.*

*Importantly, all the benchmark datasets are public, as well as their cell type annotations. To understand the complexity of the datasets, we visualized the UMAP based on the count matrices following the standard pipeline introduced in EpiScanpy [4], as shown in Figure S2 of the revised supplementary materials. In addition, we calculated the silhouette coefficient to quantify the heterogeneity of the datasets. Specifically, we performed TF-IDF on the count matrices, PCA and UMAP were subsequently performed on the transferred matrices using the default parameters. Finally, the obtained UMAP embeddings and the true cell type annotations are used to calculate the silhouette coefficient, which is to measure how well each data point is assigned to its cluster, based on the distance to other points in the same cluster and the nearest cluster. Here the cluster is the cell type annotation and the distance are calculated based on the embeddings. The silhouette coefficient ranges from -1 to 1, where a high value means that the dataset shows great compactness and diversity (more heterogeneous), and a low value means the dataset is less heterogeneous. The silhouette coefficient can be calculated as follows:*

*where is the mean distance between and all droplets in the same cell type and is the mean distance between and all droplets in the nearest cell type. The silhouette coefficient for the entire dataset is the mean of over all droplets.*

*The datasets from different data sources have distinct characteristics. The human mixed cell lines (HMC) datasets have low sequencing depth (about 5k median read pairs per droplet) but very distinct cell types. The Mouse Forebrain (MF) datasets have the sequencing depth up to 25k median read pairs per droplet and also have relatively less complexity. The mouse cell atlas (MCA) datasets that of similar sequencing depth with MF, have the most complex cell heterogeneity, as demonstrated by the UMAP plots and the low silhouette coefficient values. The additionally collected Islets datasets in this revision have about 15k median read pairs and median heterogeneity, while the additionally collected PBMC dataset has the deepest sequencing depth (up to 32k median read pairs per droplet).*

*We are sorry that the cell type markers may be not the focus of this work and are beyond our scope, but we strongly recommend obtaining the annotation information according to CellMarker database [5].*

***Figure S2.*** *The UMAP of the semi-synthetic benchmark datasets. The TF-IDF transformation was firstly performed on the count matrices, PCA and UMAP were subsequently performed on the transferred matrices using the default parameters in EpiScanpy.*

2) Supplementary table S1 shows AMULET performing better (0.405 F1) than scIBD (0.337 F1) for doublet detection rate 0.05. It is not the true that: "no matter which doublet rate was used for detection, scIBD outperformed the baseline methods on Precision, Recall, and F1." (lines 261-263).

*Response: Thank you for your valuable comment. We deeply apologize for making the wrong conclusion. We have checked the results again and added the corresponding descriptions and discussions in Lines 340 to 350 of the revised manuscript.*

*In the last version of Table S1 in supplementary materials, the results were performed on the semi-synthetic Forebrain dataset listed in Table 1, which was simulated with the 20% ground-truth heterotypic doublet rate (based on the number of singlets). To discover the detailed performance of scIBD and the baseline methods, apart from the current metrics AUROC and AUPRC, we calculated Precision, Recall and F1 under a specific cutoff, i.e., the doublet calling rate. Considering the doublet rate is unknown in reality, we enumerated the it from 0.05 to 0.25 at an interval of 0.05, and calculated the metrics on the semi-synthetic Forebrain dataset.*

*When calling doublets at the rate of 5%, we ranked the doublet scores given by the methods and obtained the 5% quantile threshold to select droplets with scores higher than the threshold as doublets. However, the doublet scores, obtained by AMULET, assigned over 8% droplets the score of one (definitely identified as doublets) for the Forebrain dataset was deeply sequenced (about 25k median read pairs per droplet). Therefore, when we used the aforementioned 5% quantile threshold which was one, more droplets (8%) were detected as doublets using AMULET, yielding its higher performance at this specific calling rate. When the calling rate increases to 0.1, scIBD surpasses AMULET in terms of all metrics. The results also demonstrate the superiority of AMULET to detect a conservative rate of doublets when the real doublet rate remains unknown, while scIBD shows its superiority when aiming at calling more doublets.*

*To give a deeper insight into understanding the performance of scIBD and the baseline methods, the detailed performance metrics on other semi-synthetic datasets are listed in Table S1 of the revised supplementary materials. The results demonstrate that scIBD outperforms the baseline methods in terms of Precision, Recall and F1 at most doublet calling rates across different datasets.*

3) How well does scIBD perform when different cell populations have similar epigenetic profiles? It would be helpful to test scIBD on such datasets, for example, the available datasets from AMULET (Thibodeau & Eroglu et al. 2021): human pancreatic islets and human peripheral blood mononuclear cells (PBMCs).

*Response: We appreciate the referee for the constructive comments. We additionally performed the benchmarking experiments for the newly-collected datasets with similar epigenetic profiles, the results and the discussion are present in Lines 277 to 323 of the revised manuscript, as well as Figure 6 of the revised manuscript and Table S2 of the revised supplementary materials.*

*In our last version of the manuscript, we have benchmarked scIBD on the datasets with low heterogeneity. As shown in Table 1 and Figure S2 of the revised manuscript, for example, the MCA datasets have low silhouette coefficient values, and the low heterogeneity illustrated in the UMAP plots. The benchmarking results have demonstrated the superiority of scIBD on these datasets. Except for the low heterogeneity, the MCA datasets have different data sizes, dimensions, sparsity, sequencing depth, and doublet rates, and scIBD shows robust excellent performance across these various datasets.*

*Following the referee’s suggestion, we tried to download the human pancreatic islets and human peripheral blood mononuclear cells datasets from AMULET (Thibodeau & Eroglu et al. 2021). The human pancreatic islets were downloaded successfully and the results are shown in Figure 6 of the revised manuscript and Table S2 of the revised supplementary materials. However, the two datasets from human peripheral blood mononuclear cells (PBMC) datasets are only available on dbGAP [6] for restricted protocols, which requires a high-level authorized account of National Institutes of Health (NIH) to access the privileged data. Because our affiliation, Xiamen University has not applied for the authorized account, we cannot get access to the two PBMC datasets.*

*Therefore, we additionally referred to other public databases and publications to find a suitable single-cell chromatin accessible sequencing (scCAS) PBMC dataset with cell type annotations and high sequencing depth. Finally, we successfully found another public scCAS PBMC dataset on 10xGenomics, whose cell types are annotated by GLUE [7]. The additional benchmark datasets are referred to as Islet1, Islet2 and PBMC, as shown in Table 1 of the revised manuscript.*

*We performed more strict quality control to establish high-confidence singlets in the additional benchmark datasets, the Islets and the PBMC datasets. Specifically, on the Islet datasets (Islet1 and Islet2), the doublet annotations by ArchR and AMULET have been reported by Thibodeau et al. [8] and we directly removed the reported doublets. On the PBMC dataset, we performed AMULET with the default parameters, and performed AcrhR with an expected doublet rate of 10% (default is 5%) in advance to remove more possible doublets. The remaining droplets were used as high-confidence singlets to establish golden-standard benchmark datasets, and the ground-truth doublets were constructed following the pipeline in Section 5.1.2 of the revised manuscript. Briefly, we simulated the ground-truth doublets weighted by proportions of known cell type annotations, to recover the real generation of heterotypic doublets when sequencing.*

*As shown in Figure 6 of the revised manuscript and Table S2 of the revised supplementary materials, scIBD significantly outperforms the baseline methods on the additional golden-standard benchmark datasets. On the Islet1 dataset, scIBD achieves the AUROC 0.978, which is 10% higher than that (0.891) of the second-best method (SnapATAC), and achieves the AUPRC 0.913, which is 34% higher than that (0.680) of the second-best method (ArchR). On the Islet2 dataset, scIBD achieves the AUROC 0.973, which is 13% higher than that (0.862) of the second-best method (SnapATAC), and achieves the AUPRC 0.882, which is 41% higher than that (0.624) of the second-best method (AMULET). On the PBMC dataset, which has deeper sequencing depth (about 30k median valid read pairs per droplet, 15k of Islets), AMULET performs much better than on the Islets datasets and significantly outperforms SnapATAC and ArchR, but scIBD still outperforms AMULET both on AUROC and AUPRC.*

*After all, scIBD has demonstrated its outperformance on various datasets with low heterogeneity in the former version of the manuscript. In the revised manuscript, the results on the additionally-collected three datasets (Islet1, Islet2 and PBMC), further demonstrate the superiority of scIBD on the datasets where different cell populations have similar epigenetic profiles.*

4) The authors should make it clear that the doublets detected by Demuxlet represent those that are between donors and that doublets within the same donor cannot be detected by Demuxlet.

*Response: Thank you for your important suggestion. We deeply agree that the doublets annotated by Demuxlet only represent the inter-donor doublets, and we apologize for missing the corresponding descriptions. We give the discussions in Lines 178 to 189 of the revised manuscript.*

*The human mixed cell lines (HMC) datasets were generated based on the mixture of ten different cancer cell lines [1], where the doublets were annotated by Demuxlet. Demuxlet is a general doublet-detection method based on the genotype variations from bulk sequencing [2]. By identifying genotype variations, Demuxlet can basically detect those doublets formed from different donors/samples with distinct genotype variations. Therefore, in HMC datasets, the doublets detected by Demuxlet can be considered as the heterotypic doublets from different cell lines (donors). Similarly, we also aim to stress the detection of the heterotypic doublets in scCAS data, thus it is reasonable to use the HMC datasets where the ground-truth doublets were annotated by Demuxlet, as one kind of the benchmark datasets.*

*To further benchmark the performance of scIBD, more evaluation experiments were performed in the following four sections.*

*(1) The evaluations on the semi-synthetic datasets where the ground-truth doublets are manually constructed, to benchmark the performance on the complex datasets in more realistic scenes (Section 2.4 of the revised manuscript);*

*(2) The doublets-reads-down-sampling experiments to benchmark the performance of scIBD when detecting doublets with different numbers of captured reads (Section 2.5 of the revised manuscript);*

*(3) the additionally-carried benchmarking experiments on the golden-standard datasets where the doublets were firstly removed to produce high-confident singlets and the doublets were subsequently simulated (Section 2.6 of the revised manuscript);*

*(4) The complete reads-down-sampling experiments to benchmark scIBD of handling data with different sequencing depths (Section 2.6 of the revised manuscript);*

*(5) The downstream biological analyses to demonstrate the realistic function of scIBD (Section 2.7 of the revised manuscript).*

*We hope that the referee can agree with that the comprehensive benchmarking on the more extensive datasets has demonstrated the superiority of scIBD.*

5) In the caption of Figure S6, it is mentioned: "The cells that are selected to construct benchmark doublets are weighted by the known cell annotations, namely, the larger size the cell type holds, the more likely it may generate a heterotypic doublet". More details should be provided for this construction of heterotypic doublets in the semi-synthetic evaluations in methods. How similar is this doublet selection for evaluation to the artificial doublet creation criteria used by scIBD to detect doublets? It would be helpful to understand the performances when this weighting criteria is not used for generating doublets for evaluation.

*Response: We appreciate the constructive comment. We described the pipeline of simulating ground-truth doublets more concretely in Lines 523 to 529 of the revised manuscript and the captions of Figure S8 in the revised supplementary materials. We also additionally constructed two datasets where the ground-truth doublets were simulated without the weighting criterion, to investigate the performance changes of the methods on such datasets. The results and discussions are present in Lines 448 to 463 of the revised manuscript and Table S7 of the revised manuscript.*

*First of all, we aim to stress the heterotypic doublets which significantly disturb the downstream analyses. However, the totally-random sampling strategy, namely, randomly selected two droplets to form a ground-truth doublet, may result in a bunch of homotypic doublets, which deviates from our essential purpose. Also, weighted by the known cell type sizes, is the strategy analogous to the intrinsic formation when sequencing as described in AMULET [8] that ‘The likelihood of a cell type to form multiplets is associated with its frequency within the tissue’. Therefore, we adopted the weighting criteria to generate ground-truth doublets for comprehensive benchmarking on semi-synthetic datasets. Specifically, we obtained the proportions of all cell types based on their annotations; according to the cell type proportions we then probabilistically selected two cell types, from which one cell was respectively randomly selected to form a doublet. This simulation pipeline was repeatedly performed to simulate all ground-truth doublets. We believe that this simulation pipeline can construct the high-confidence heterotypic doublets as the real-sequencing scenario. Under this assumption, the pseudo-dropleting-simulation strategy is accordingly used to simulate high-confidence heterotypic doublets in scIBD, which tries to recover the generation of heterotypic doublets in real sequencing.*

*To investigate the performance of different methods on the datasets without the weighting criteria, we additionally constructed two semi-synthetic datasets based on the two Islet datasets where the doublets were simulated by randomly picking two cell types, namely, the weighting strategy was discarded. Except for the weighted simulation, all other simulation steps remained the same as the Islet datasets in Table 1.*

*As shown in Table S7 of the revised supplementary materials, we can observe that the performance of the simulation-based methods (scIBD, SnapATAC and AcrhR) all decrease on the datasets where the ground-truth doublets were simulated without the weighting criteria. Specifically, SnapATAC achieves the AUROC from 0.89 to 0.73 on islet1, from 0.86 to 0.69 on islet2, and achieves the AUPRC from 0.61 to 0.49 on islet1, from 0.61 to 0.40 on islet2; ArchR achieves the AUROC from 0.87 to 0.78 on islet1, from 0.82 to 0.79 on islet2, and achieves the AUPRC from 0.68 to 0.53 on islet1, from 0.49 to 0.48 on islet2; scIBD achieves the AUROC from 0.98 to 0.92 on islet1, from 0.97 to 0.93 on islet2, and achieves the AUPRC from 0.91 to 0.70 on islet1, from 0.88 to 0.64 on islet2. The drastic decrease in performance of the simulation-based methods may be due to the bias between the simulated doublets and ground-truth doublets. However, the performance of AMULET, which is read-based, achieves nearly identical results on the datasets for that it only performs statistical analyses on the overlapped reads, regardless of the different cell type origins, also suggesting its robustness in detecting the homotypic doublets. Although scIBD also achieves decreased performance on the unweighted-datasets, it still outperforms the baseline methods.*

6) In the current schema for semi-synthetic evaluations, artificial doublets are generated to provide a ground truth set of doublets. However, this only allows for a proper estimate of recall. The ground truth for singlets may still contain doublets. The authors should discuss the caveats of this approach when evaluating performances. Some additional analyses are needed to understand precision. For example, evaluating singlet cells to establish high confidence singlets.

*Response: Thank you for the insightful comment. We deeply agree that in current benchmark datasets, the singlets may still contain doublets, which may yield biased Precision. Therefore, following the suggestion of the referee, we applied stricter quality control processes to establish high-confidence singlets on the additionally-collected datasets, Islet1, Islet2 and PBMC. the results and the discussion are present in Lines 277 to 323 and Figure 6 of the revised manuscript, as well as the Table S2 of the revised supplementary materials.*

***Figure 6a.*** *The performance comparison on the golden-standard benchmark datasets*

*Specifically, we first downloaded the raw data of the Islets datasets (Islet1 and Islet2) and PBMC dataset, as well as the cell type annotations from AMULET [8] and GLUE [7]. On the Islet datasets (Islet1 and Islet2), the doublet annotations by ArchR and AMULET have been reported by Thibodeau et al. [8] and we directly removed the reported doublets. On the PBMC dataset, we performed AMULET with the default parameters, and performed AcrhR with an expected doublet rate of 10% (default is 5%) in advance to remove more possible doublets. We believe the remaining droplets can be considered as high confidence singlets. Based on the remaining singlets, the ground-truth doublets were constructed according to the simulation pipeline as described in Section 5.1.2 of the revised manuscript. Briefly, we simulated the ground-truth doublets weighted by proportions of known cell type annotations, to recover the real generation of heterotypic doublets when sequencing.*

*As shown in Figure 6a of the revised manuscript and Table S2 of the revised supplementary materials, scIBD significantly outperforms the baseline methods on such amended golden-standard datasets. In the revised manuscript, we additionally calculated more metrics, including Precision, Recall, and F1, to more comprehensively describe the performance. As shown in Table S1 and Figure S2 of the revised supplementary materials, scIBD basically outperforms the baseline methods in terms of all metrics on the additional benchmark datasets, Islet1, Islet2 and PBMC.*

7) Results are demonstrated for up to ~15k median reads per cell (Mouse Forebrain dataset). It would be helpful to demonstrate scIBD's performance for more deeply sequenced data to understand how well scIBD scales.

*Response: We appreciate the referee for the valuable comment. We give the related results and discussions to evaluate the effect of sequencing depth on the performance in Lines 277 to 323 and Figure 6 of the revised manuscript, as well as Table S7 of the revised supplementary materials.*

*We additionally list the sequencing depth statistical results (Total valid read pairs & valid read pairs per droplet) for all benchmark datasets, as shown in the revised manuscript Table 1. Of note, all the droplets used in our benchmark datasets are those with cell type annotations that are publicly reported, which means that a portion of droplets with low quality has been removed from the raw data. Therefore, the mean/median reads per droplet are normally higher than those reported originally. For example, when only using the annotated droplets in Mouse Forebrain (MF) dataset, the median valid read pairs have increased up to about 25k per droplet. The datasets form MCA have similar sequencing depth, some of which have higher mean valid read pairs per droplet, such as the Bone marrow and the Prefrontal cortex datasets, having the sequencing depth up to 30k read pairs per droplet. The benchmarking results in the last version of the manuscript have demonstrated that scIBD shows outperformance on these datasets with high sequencing depth.*

*Following the suggestion of the referee, to demonstrate the performance of scIBD on the more deeply sequenced data, we further evaluated the results on another deep-sequenced scCAS PBMC data from 10xGenomics, which has about 30k median valid read pairs per nuclei/droplet after quality control. The cell type annotations are derived from GLUE [7]. We used a stricter quality control strategy to establish singlets and then constructed the datasets with higher quality. Briefly, we filtered out the high-confidence doublets using AMULET and ArchR to reserve the high-confidence singlets, based on which ground-truth doublets were simulated to benchmark the methods. As shown in Figure 6a and Table S2 of the revised manuscript, scIBD outperforms the baseline methods. Particularly, AMULET performs better on the deeper-sequenced data compared to Islet datasets which has lower sequencing depth.*

*We further conducted the down-sampling experiments on the Islet1 and the PBMC datasets, to investigate the relation between the performance and the sequencing depth. On the Islet1 dataset that has lower sequencing depth (about 15k valid read pairs per droplet), we down-sampled the reads of all droplets using the rate ranging from 0.1 to 0.4 with an interval of 0.1; on the PBMC dataset that has deeper sequencing depth (about 30k valid read pairs per droplet), we down-sampled the reads more drastically from 0.1 to 0.5 with an interval of 0.2. As is shown in the revised manuscript Figure 6b, scIBD achieves the best performance no matter on the aspect of AUROC and AUPRC on the datasets with different sequencing depths. On the Islet1 dataset, scIBD outperforms the secondary method by about 10% on the mean of AUROC and even about 35% on the mean of AUPRC. On the PBMC dataset, AMULET achieves the second-best performance for the high sequencing depth. However, its performance decreases drastically when the down-sampling rate increases up to 0.5 (the sequencing depth has decreased to 15k median read pairs per droplet). After all, the down-sampling experiments demonstrate that AMULET achieves better performance on the deeper-sequenced data. However, the simulation-based methods, show the relatively-high robustness when handling data with different sequencing depths, among which scIBD is the overall best.*

**Figure 6b. The performance evaluation on the down-sampled Islet1 and PBMC datasets.** The reads of Islet1 dataset are down-sampled with a ratio ranging from 0.1 to 0.4 with an interval of 0.1; and the reads of PBMC dataset are down-sampled with the ratio ranging from 0.1 to 0.5 with an interval of 0.2. The AUROC (solid lines) and the AUPRC (dotted lines) show the trend of performance with the decrease in sequencing depth.

8) There is a bug when trying to use the tool when it tries to pass 'min_samples' to DBSCAN in the GetStrategy method. The min_samples passed ends up being a floating point number instead of an integer. "The 'min_samples' parameter of DBSCAN must be an int in the range [1, inf). Got 31.67 instead."

*Response: Thank you for pointing out the code bug. We deeply apologize for the mistake. We have resolved the bug in the tool by transferring the value from float to int. The ‘min_samples’ parameter in DBSCAN represents the minimum size of the clusters, which helps us to control the clustering results. In our clustering pipeline, it is set as 0.5% of the number of input droplets. We have tested the scIBD tool on different devices to ensure its usability.*

Minor:

1) Precision and recall should be reported for the fully synthetic data evaluation and other areas where these calculations are missing.

*Response: Thank you for your constructive suggestion. All the datasets are comprehensively evaluated, and the corresponding descriptions are present in Lines 239 to 255 of the revised manuscript. The detailed metrics for semi-synthetic datasets are shown in Table S1 of the revised supplementary materials*

*In the fully-synthetic datasets, we also call the doublets using the ground-truth doublet rate. On the balanced dataset, the Precision is 0.99 and the Recall is 0.99, on the imbalance dataset, the Precision is 0.97 and the Recall is 0.97. On the semi-synthetic datasets, we have incorporated the detailed metrics, including Precision, Recall and F1, to provide a comprehensive description of the performance of scIBD and the baseline methods. As shown in Table S1 of the revised supplementary materials, scIBD outperforms the baseline methods in terms of Precision, Recall and F1 at most scenarios.*

2) It would be helpful to understand the runtime performance of scIBD in comparison with other methods.

*Response: We appreciate the referee for this insightful comment. We have implemented the experiments and present the results and discussion in Lines 445 to 447 of the revised manuscript, and Content S2 and Table S6 of the revised manuscript.*

*scIBD is an iterative-optimizing method to progressively detect the doublets. In each iteration, scIBD need to implement the re-clustering, re-simulating and re-detecting, thus it may consume more time on some datasets where the doublets are hard to detect. We have diligently optimized the speed of our algorithm by utilizing sparse-matrix operation in several steps of scIBD, including the TF-IDF transformation when clustering, the Jaccard distance calculation when detecting, to reduce the time taken in each iteration in the revised version of scIBD tool.*

*The latest version of our algorithm has achieved remarkable improvements in both running time and memory usage, surpassing the previous version by a large margin. For instance, on the Forebrain dataset, the running time has been slashed from about 10 minutes to a mere 2 minutes, and the peak memory usage has been reduced from about 3.00GB to a stunning 0.95GB.*

*To more comprehensively compare the computational efficacy of scIBD and the baseline methods, we selected several datasets with different data sizes and recorded the complete running time and the peak memory usage on a Linux server equipped with a 24-cores Intel(R) Xeon(R) CPU E5-2620 v2 @ 2.10GHz. All methods were executed with their default settings except for the expected doublet calling rate (set as the ground-truth doublet rate).*

*As shown in Table S6 of the revised supplementary, regarding running time, SnapATAC (Scrublet) emerges as the fastest method, while the other three methods, scIBD, ArchR and AMULET, exhibit longer running time and demonstrate varying advantages on different datasets. Regarding memory usage, AMULET acts as the best method for that it only computes the statistical results of the sequencing reads based on BAM/fragment files, while the other three methods need the converted count matrices to detect the doublets. No matter in terms of running time or memory usage, scIBD generally achieves the second-best efficacy across the datasets.*

*Although SnapATAC (Scrublet) achieves the best efficacy performance on running time, it shows unsatisfactory memory usage, being approximately double or triple that of scIBD. Moreover, it also achieves fluctuating performance across the benchmark datasets, which may due to it was initially proposed for scRNA-seq data. Compared with AMULET that shows the best memory usage efficacy, scIBD achieves the comparable time taken, only lagging about one to two minutes on most datasets. AMULET also achieves the relatively good performance in detecting doublets on some datasets with high sequencing depth. However, for the nature that it detects doublets based on the statistical results of the reads, it shows sensitivity to the sequencing depth, yielding unstable doublet-detection performance on the lower-sequenced datasets.*

*In conclusion, although scIBD and the baseline methods have their unique advantages in terms of running time and memory usage, scIBD achieves the best-balanced performance on both computational efficacy and doublet detection efficacy. Users can select the most suitable method based on their specific requirements and constraints.*

3) It would be helpful for the authors to briefly discuss the typical doublet rate observed in scCAS data (e.g., what's described in Lareau et al. 2020, reference 51) to help readers understand performances observed in Table S1.

*Response: Thank you for your valuable suggestion. We give the discussions in Lines 340 to 350 of the revised manuscript.*

*According to Lareau et al. [3], another type of doublets, namely, barcode doublet, is introduced, which can also confound the single-cell analyses. It is a more complicated issue compared with the doublets introduced in this work. Lareau et al. reported that approximately 4% of droplets from the 10x platform may contain multiple beads and approximately 5% of beads may contain detectable levels of multiple oligonucleotide barcodes. The typical barcode doublet rate of “barcode doublet” is an analogy to that of “regular doublet” reported by Xi et al. [9]. However, we also found that when the number of sequenced cells increases, the rate of doublets can accordingly increase, especially after quality control where the low-quality cells were removed while the doublets always escaped from normal quality control. This is also supported by the results from the HMC datasets, the low-loading dataset has about 20% doublet rate (total of 12,793 droplets) while the high-loading one has about 30% doublet rate (total of 20,663 droplets) based on the annotation information by Demuxlet.*

*We simulated the doublets for semi-synthetic datasets with a value of 20% based on the number of singlets. In Table S1 of the last version of supplementary materials, the results were from the experiments performed on the semi-synthetic Forebrain dataset with the doublet simulation rate of 20% (based on the singlets, resulting in approximately 16% doublet rate of full droplets). Specifically, when calling doublets at the rate of 5%, we ranked droplets based on the doublet scores given by the methods and obtained the 5% quantile threshold (cutoff). Any droplet with a score above the cutoff was considered a doublet. However, the doublet scores (the probabilistic values) obtained by AMULET, gave over 8% of droplets the score of one (definitely identified as doublets). Therefore, more droplets (8%) are detected as doublets at the 5% calling rate using AMULET, yielding its higher Recall in this specific scenario. However, it also demonstrated the superiority of AMULET when calling a conservative rate of doublets when the doublet rate is unknown.*

*We appreciate the referee again for the valuable and constructive comments and suggestions. We hope the additional experiments and the results in the revised manuscript can more comprehensively demonstrate the superiority of scIBD.*

***References***

*1.	Granja JM, Corces MR, Pierce SE, Bagdatli ST, Choudhry H, Chang HWY, Greenleaf WJ:* ***ArchR is a scalable software package for integrative single-cell chromatin accessibility analysis (vol 53, pg 403, 2021).*** *Nature Genetics 2021,* ***53:****935-935.*

*2.	Kang HM, Subramaniam M, Targ S, Nguyen M, Maliskova L, McCarthy E, Wan E, Wong S, Byrnes L, Lanata CM, et al:* ***Multiplexed droplet single-cell RNA-sequencing using natural genetic variation (vol 36, pg 89, 2018).*** *Nature Biotechnology 2020,* ***38:****1356-1356.*

*3.	Lareau CA, Ma S, Duarte FM, Buenrostro JD:* ***Inference and effects of barcode multiplets in droplet-based single-cell assays.*** *Nature Communications 2020,* ***11:****1-19.*

*4.	Danese A, Richter ML, Chaichoompu K, Fischer DS, Theis FJ, Colomé-Tatché M:* ***EpiScanpy: integrated single-cell epigenomic analysis.*** *Nature Communications 2021,* ***12:****5228.*

*5.	Zhang X, Lan Y, Xu J, Quan F, Zhao E, Deng C, Luo T, Xu L, Liao G, Yan M:* ***CellMarker: a manually curated resource of cell markers in human and mouse.*** *Nucleic acids research 2019,* ***47:****D721-D728.*

*6.	Mailman MD, Feolo M, Jin Y, Kimura M, Tryka K, Bagoutdinov R, Hao L, Kiang A, Paschall J, Phan L:* ***The NCBI dbGaP database of genotypes and phenotypes.*** *Nature genetics 2007,* ***39:****1181-1186.*

*7.	Cao ZJ, Gao G:* ***Multi-omics single-cell data integration and regulatory inference with graph-linked embedding.*** *Nat Biotechnol 2022,* ***40:****1458-1466.*

*8.	Thibodeau A, Eroglu A, McGinnis CS, Lawlor N, Nehar-Belaid D, Kursawe R, Marches R, Conrad DN, Kuchel GA, Gartner ZJ, et al:* ***AMULET: a novel read count-based method for effective multiplet detection from single nucleus ATAC-seq data.*** *Genome Biology 2021,* ***22:****1-19.*

*9.	Xi NM, Li JJ:* ***Benchmarking Computational Doublet-Detection Methods for Single-Cell RNA Sequencing Data.*** *Cell Systems 2021,* ***12:****176-194.*

**Second round of review**

**Reviewer 1**

All my previous comments has been reasonably addressed. I do not have any further comments.

**Reviewer 2**

The latest revision is a significant improvement over the initial submission, and the majority of comments have been successfully addressed. The new Table 1 is much appreciated as it helps to understand the strengths and weaknesses of all algorithms across the benchmarks. The inclusion of islet and PBMC data establishes the robustness of the method across a variety of datasets with respect to read depth and cell type similarity. Furthermore, comparisons using the unweighted simulation scheme are extremely helpful to demonstrate that the performance of scIBD is not simply the result of bias introduced in the selection strategy.

It is also much appreciated that the bug previously reported has been fixed. Testing scIBD was a success overall. The test also incorporated 306 artificial doublets selected from the data itself, 144 of which passed filters using ArchR. I was pleased to find that 123/144 (85%) of these artificial doublets were detected by scIBD, while ArchR could only identify 101/144 (70%). This further convinced me that scIBD is an improvement over existing methods.

Below are my remaining (minor) comments :

1. I do not believe that the term “gold standard” should be used for the islet and PBMC dataset. Identifying high purity singlets using AMULET and ArchR raises questions on whether some doublets were missed, and I’m not convinced that this is the best available benchmark. In my opinion, it would be better to refrain from using this term as it may suggest that future analyses must use these datasets in this way, which I don’t believe is true as there are additional available ways of improving this benchmark (i.e., determining purity by looking at accessibility profiles of known cell type markers)

2. Please include more clarity on whether the unweighted simulation analysis included or excluded the doublets detected by AMULET and ArchR.

Suggestion: Figure 5 may be better placed in supplementary with the addition of new figures.

**Authors Response**

**Point-by-point responses to the reviewers’ comments:**

Reviewer #2:

The latest revision is a significant improvement over the initial submission, and the majority of comments have been successfully addressed. The new Table 1 is much appreciated as it helps to understand the strengths and weaknesses of all algorithms across the benchmarks. The inclusion of islet and PBMC data establishes the robustness of the method across a variety of datasets with respect to read depth and cell type similarity. Furthermore, comparisons using the unweighted simulation scheme are extremely helpful to demonstrate that the performance of scIBD is not simply the result of bias introduced in the selection strategy.

It is also much appreciated that the bug previously reported has been fixed. Testing scIBD was a success overall. The test also incorporated 306 artificial doublets selected from the data itself, 144 of which passed filters using ArchR. I was pleased to find that 123/144 (85%) of these artificial doublets were detected by scIBD, while ArchR could only identify 101/144 (70%). This further convinced me that scIBD is an improvement over existing methods.

*Response: We appreciate the referee for the constructive comments. We are also grateful for the referee to comprehensively evaluate scIBD by practically testing the scIBD tool on additional datasets. The suggestions and advice were constructive and significantly improved the quality of the paper. We have addressed all comments, as described in the point-to-point response below.*

I do not believe that the term “gold standard” should be used for the islet and PBMC dataset. Identifying high purity singlets using AMULET and ArchR raises questions on whether some doublets were missed, and I’m not convinced that this is the best available benchmark. In my opinion, it would be better to refrain from using this term as it may suggest that future analyses must use these datasets in this way, which I don’t believe is true as there are additional available ways of improving this benchmark (i.e., determining purity by looking at accessibility profiles of known cell type markers).

*Response: Thank you for your valuable comment. We deeply agree that the term ‘golden-standard’ that describes the constructed benchmark datasets is too absolute and many additional methods can be applied to improve the benchmark. We deeply apologize for our inconsideration of making the descriptions. Following this suggestion, we changed the term ‘golden-standard datasets’ to ‘rigorously-quality-controlled datasets’, to reveal that we constructed the benchmark datasets where the singlets were established after removing the potential doublets using ArchR and AMULET. The corresponding modifications are presented in the revised manuscript (Section Results, Subsection Benchmarking on the rigorously-quality-controlled semi-synthetic datasets).*

Please include more clarity on whether the unweighted simulation analysis included or excluded the doublets detected by AMULET and ArchR.

*Response: We appreciate the insightful suggestion. We apologize for missing the detailed descriptions. We have added the corresponding additional descriptions in our revised manuscript (paragraph two in Section Discussion).*

*Specifically, except for the weighted simulation strategy, all other steps in constructing the unweighted datasets remain the same as the benchmark Islets datasets. The same potential doublets in raw data were removed in advance using ArchR and AMULET to establish high-quality singlets.*

Figure 5 may be better placed in supplementary with the addition of new figures.

*Response: Thank you for your constructive suggestion. However, we cannot precisely understand the suggestion you provided. Figure5 illustrates the performance of scIBD on the datasets where the ground-truth doublets were constructed with different numbers of captured reads, while the addition of new figures (Fig. 6) illustrates the performance of scIBD on the additional benchmark datasets (Islets and PBMC). The reads-down-sampling experiments are distinct experiments to demonstrate different aspects of the robustness of scIBD. Therefore, we think that the two figures can not be placed together and the current figure depositions may be suitable for understanding the performance of scIBD. If you have further suggestions, can you provide us with more details about the figure deposition?*
